# Supplementary material for: Estimating the gap between demand and supply of medical appointments by physicians for hypertension care: a pooled analysis in 191 countries
Source: BMJ Open. 2022 Apr 3;12(4):e059933. doi: 10.1136/bmjopen-2021-059933 (PMC8981295; doi:10.1136/bmjopen-2021-059933)
Supplement: Supplementary data [file bmjopen-2021-059933supp001.pdf]

SUPPLEMENTARY MATERIAL

Estimating the gap between demand and supply of medical appointments by physicians for hypertension care: A pooled analysis in 191 countries

Corresponding Author

Rodrigo M Carrillo-Larco, MD

Department of Epidemiology and Biostatistics, School of Public Health, Imperial College London, London, UK.

r.carrillo-larco@imperial.ac.uk

EXPANDED METHODS 3

Data sources 3

Data management 4

New variables 4

References 6

EXPANDED METHODS

All analysis codes are available as Supplementary Materials.

Data sources

We combined multiple data sources.

- Hypertension[1, 2] data from the Non-Communicable Disease Risk Factor Collaboration (NCD-RisC). These data included prevalence of hypertension, and amongst people with hypertension, these data also provided the proportion of diagnosed (i.e., awareness), treated and controlled. These data are for all countries and for all years until 2019. All these hypertension estimates are sex-stratified and for the age group 30-79 years.
- The number of physicians[3] was retrieved from the World Health Organization (WHO) observatory. These data are for all countries, though the latest available data may be different by country (i.e., not all countries have data for the same years). The most recent data was for the year 2019 (not all countries have data until 2019).

Year	Number of countries with data	Proportion of all data
2004	1	0.005
2008	1	0.005
2009	1	0.005
2010	1	0.005
2011	1	0.005
2012	3	0.016
2013	3	0.016
2014	16	0.084
2015	14	0.073
2016	17	0.089
2017	32	0.168
2018	60	0.314
2019	41	0.215

- Population[4] data (number of inhabitants per country) was retrieved from the Institute for Health Metrics and Evaluation (IHME). We extracted the number of men and women in the age group 30-79 for all years until 2019.
- The World Bank classification[5] was used to label countries according to income (low, middle and high income) and world region. We only used the latest available data.

Data management

We downloaded each data source. In the dataset for number of physicians we only kept the most recent year per country. The hypertension data and the number of physicians data were merged matching by year and country; in other words, both data sources belonged to the same year. These data were then merged with population data matching by year, country and sex (because hypertension data were sex-stratified). Matching by country, we then merged with the World Bank data; the World Bank data would not necessarily be synchronic with the other data. Overall, hypertension, number of physicians and population data was synchronic (belonged to the same year).

New variables

The sex-specific prevalence of hypertension was multiplied by the number of men and women in each country, to compute the absolute number of men and women with hypertension. These sex-specific absolute numbers of people with hypertension were then multiplied by the proportion of diagnosed, treated and controlled; this, to compute the absolute number of people with diagnosed, treated and controlled hypertension. Because all these estimates were sex-specific, we summed them within each country to have one estimate per country (i.e., overall by country rather than sex-stratified). For diagnosis, treatment and control rates, we followed a similar procedure to move from sex-stratified estimates to one-only per country.

We computed the number of medical appointments needed by people with hypertension, hereafter referred to as *demand*. We multiplied the absolute number of people with hypertension by [1…6…12]. If people with hypertension would need 1 medical appointment per year, then there would be as many medical appointment as people with hypertension (multiplication by 1). On the other hand, if people with hypertension would need 12 medical appointments per year, then there

would be as many medical appointments as 12 times the number of people with hypertension (multiplication by 12). The same logic would apply for all natural numbers between 1 and 12.

We computed the number of medical appointments available for people with hypertension, hereafter referred to as *offer*. For this, we multiplied the number of physicians by 5,000; this factor was conceived assuming that a physician could see 25 patients per day and work 200 days per year (25 x 200 = 5,000). The product between the number of physicians and 5,000 was then multiplied by 0.10, because we assumed that 10% of their time was dedicated to hypertension care.

We computed the gap between the *offer* and *demand* (*offer* - *demand*), as a simple subtraction between the number of medical appointments available (*offer*) and the number of medical appointments needed by people with hypertension (*demand*). We did this subtraction 12 times, for each of the potential number of medical appointments per year (1 through 12, as described in the second paragraph of this section above).

A negative gap would mean that the *demand* exceeded the *offer*. In other words, the healthcare system would not have enough physicians to meet the needs of medical appointments per year for all patients with hypertension.

Carrillo-Larco RM, *et al. BMJ Open* 2022; 12:e059933. doi: 10.1136/bmjopen-2021-059933

References

1. Non-Communicable Diseases Risk Factor Collaboration (NCD-RisC). [cited 2021 Sept 23]. Available from: https://ncdrisc.org/.

2. NCD Risk Factor Collaboration (NCD-RisC). Worldwide trends in hypertension prevalence and progress in treatment and control from 1990 to 2019: a pooled analysis of 1201 population-representative studies with 104 million participants. *Lancet (London, England)* 2021; **398**(10304): 957-80.

3. The Global Health Observatory - Medical doctors (number). [cited 2021 Sep 23]. Available from: https://www.who.int/data/gho/data/indicators/indicator-details/GHO/medical-doctors-(number).

4. Global Burden of Disease Study 2019 (GBD 2019) Population Estimates 1950-2019. [cited 2021 Sep 23]. Available from: http://ghdx.healthdata.org/record/ihme-data/gbd-2019-population-estimates-1950-2019.

5. World Bank Country and Leading Groups. [cited 2021 Sep 23]. Available from: https://datahelpdesk.worldbank.org/knowledgebase/articles/906519-world-bank-country-and-lending-groups.
