# Supplementary material for: Estimating the gap between demand and supply of medical appointments by physicians for hypertension care: a pooled analysis in 191 countries
Source: BMJ Open. 2022 Apr 3;12(4):e059933. doi: 10.1136/bmjopen-2021-059933 (PMC8981295; doi:10.1136/bmjopen-2021-059933)
Supplement: Author's manuscript [file bmjopen-2021-059933.draft_revisions.pdf]

BMJ Open

BMJ Open is committed to open peer review. As part of this commitment we make the peer review history of every article we publish publicly available.

When an article is published we post the peer reviewers' comments and the authors' responses online. We also post the versions of the paper that were used during peer review. These are the versions that the peer review comments apply to.

The versions of the paper that follow are the versions that were submitted during the peer review process. They are not the versions of record or the final published versions. They should not be cited or distributed as the published version of this manuscript.

BMJ Open is an open access journal and the full, final, typeset and author-corrected version of record of the manuscript is available on our site with no access controls, subscription charges or pay-per-view fees (http://bmjopen.bmj.com).

If you have any questions on BMJ Open's open peer review process please email info.bmjopen@bmj.com

BMJ Open

Gap between demand and offer of medical appointments for hypertension care: A global analysis

Journal:	*BMJ Open*
Manuscript ID	bmjopen-2021-059933
Article Type:	Original research
Date Submitted by the Author:	07-Dec-2021
Complete List of Authors:	Carrillo Larco, Rodrigo M. ; Imperial College London,<br>Guzman-Vilca, Wilmer Cristobal; CRONICAS Centre of Excellence in Chronic Diseases<br>Neupane, Dinesh; Johns Hopkins University
Keywords:	Epidemiology < TROPICAL MEDICINE, Public health < INFECTIOUS DISEASES, Hypertension < CARDIOLOGY

SCHOLARONE™
Manuscripts

I, the Submitting Author has the right to grant and does grant on behalf of all authors of the Work (as defined in the below author licence), an exclusive licence and/or a non-exclusive licence for contributions from authors who are: i) UK Crown employees; ii) where BMJ has agreed a CC-BY licence shall apply, and/or iii) in accordance with the terms applicable for US Federal Government officers or employees acting as part of their official duties; on a worldwide, perpetual, irrevocable, royalty-free basis to BMJ Publishing Group Ltd ("BMJ") its licensees and where the relevant Journal is co-owned by BMJ to the co-owners of the Journal, to publish the Work in this journal and any other BMJ products and to exploit all rights, as set out in our licence.

The Submitting Author accepts and understands that any supply made under these terms is made by BMJ to the Submitting Author unless you are acting as an employee on behalf of your employer or a postgraduate student of an affiliated institution which is paying any applicable article publishing charge ("APC") for Open Access articles. Where the Submitting Author wishes to make the Work available on an Open Access basis (and intends to pay the relevant APC), the terms of reuse of such Open Access shall be governed by a Creative Commons licence – details of these licences and which Creative Commons licence will apply to this Work are set out in our licence referred to above.

Other than as permitted in any relevant BMJ Author's Self Archiving Policies, I confirm this Work has not been accepted for publication elsewhere, is not being considered for publication elsewhere and does not duplicate material already published. I confirm all authors consent to publication of this Work and authorise the granting of this licence.

For peer review only

Gap between demand and offer of medical appointments for hypertension care: A global analysis

Rodrigo M. Carrillo-Larco[1,2]

Wilmer Cristobal Guzman-Vilca[2,3,4]

Dinesh Neupane[5]

1. Department of Epidemiology and Biostatistics, School of Public Health, Imperial College London, London, UK.
2. CRONICAS Centre of Excellence in Chronic Diseases, Universidad Peruana Cayetano Heredia, Lima, Peru.
3. School of Medicine "Alberto Hurtado", Universidad Peruana Cayetano Heredia, Lima, Peru.
4. Sociedad Científica de Estudiantes de Medicina Cayetano Heredia (SOCEMCH), Universidad Peruana Cayetano Heredia, Lima, Peru.
5. Department of Epidemiology, Welch Center for Prevention, Epidemiology and Clinical Research, Johns Hopkins University, Baltimore, MD, USA.

Corresponding Author

Rodrigo M Carrillo-Larco, MD

Department of Epidemiology and Biostatistics, School of Public Health, Imperial College London, London, UK.

rcaarrill@ic.ac.uk

For peer review only

ABSTRACT

Introduction: With a growing number of people with hypertension, the limited number of physicians could not to provide treatment to all patients. We quantified the gap between medical appointments available and needed for hypertension care, overall and in relation to hypertension treatment cascade metrics.

Methods: Ecological descriptive analysis. We combined country-year-specific data on hypertension prevalence, awareness, treatment and control (from NCD-RisC) and number of physicians (from WHO). We estimated from 1 to 12 medical appointments per year for hypertensive patients. We assumed that physicians could see 25 patients per day, work 200 days per year, and dedicate 10% of their time to hypertension care.

Results: We studied 191 countries. Forty-one countries would not have enough physicians to provide at least 1 medical appointment per year to all the population with hypertension; these countries were low/lower-middle income and in Sub-Saharan Africa or East Asia and Pacific. Regardless of the world region, ≥50% of countries would not have enough physicians to provide ≥8 medical appointments to their population with hypertension. Countries where the demand exceeded the offer of medical appointments for hypertension care had worse hypertension diagnosis, treatment and control rates than countries where the demand did not exceed the offer. There were positive correlations between the physician density and hypertension diagnosis (r=0.70, p<0.001), treatment (r=0.70, p<0.001) and control (r=0.59, p<0.001).

Conclusions: Where physicians are the only healthcare professionals allowed to prescribe antihypertensive medications, particularly in low- and middle-income countries, the healthcare system may struggle to deliver antihypertensive treatment to hypertensive patients.

Key words: non-communicable diseases; population health; health metrics.

For peer review only - http://bmjopen.bmj.com/site/about/guidelines.xhtml

Strengths and limitations

- We built on previous research which proposed a solid and reasonable methodology to quantify the gap between the *offer* and *demand* of medical appointments for hypertension care at the country level.
- All of our results are not for the same year. Potential country-specific users of our results are advised to understand our estimates taking into consideration the data year.
- Because of hypertension data availability, we restricted our analysis to the age group 30-79 years.
- Third, we used the number of physicians as reported by the World Health Organization, and assumed each physician sees 25 patients per day and works 200 days per year. We are not aware of empirical data to support this (or other) scenario.
- Fourth, country-level estimates often hide within country differences.

For peer review only

INTRODUCTION

Globally, high blood pressure is the leading risk factor for cardiovascular morbidity and mortality.[1] Even though there are effective treatments for hypertension,[2] these do not always reach the patients leading to low hypertension treatment and control rates.[3, 4] Reasons for poor access to antihypertensive treatment could include lack of medicines, inability to afford treatment, and not having health insurance. However, where universal health coverage is available, the obstacle to provide treatment for patients with hypertension could be the shortage of physicians,[5] particularly where physicians are the only health professionals authorized to prescribe medicines for hypertension. Due to the lifelong nature of antihypertensive treatment, people with hypertension will need multiple medical appointments per year whereby physicians will titrate and refill their medicines. While the number of people with hypertension grows faster than the number of physicians, and hypertension diagnosis rates increases,[4] countries will struggle to provide the medical appointments needed for patients with hypertension; that is, there will be more medical appointments needed than available for hypertension care.

The gap between the number of medical appointments needed and available for people with hypertension has been quantified showing that several countries do not have enough physicians for three, six or 12 medical appointments per year for people with hypertension.[6] Nonetheless, previous estimates were up to 2015 and the authors did not study the gap in relation to hypertension care cascade metrics: diagnosis, treatment and control. Using more recent data will provide countries with up-to-date evidence to inform their policies, interventions, and targets. Also, the correlations between the gap and hypertension care cascade metrics will inform if countries with larger gaps also have worse hypertension treatment and control rates.

Consequently, elaborating on previous evidence and overcoming the above described limitations,[6] we quantified the gap between the number of medical appointments needed by people with hypertension and the number of medical appointments available for hypertension care based on the latest available data.

METHODS

This is an ecological descriptive analysis. We combined data from different sources to quantify the hypertension gap regarding *demand* of medical appointments by people with hypertension

For peer review only - http://bmjopen.bmj.com/site/about/guidelines.xhtml

and the availability of physicians to provide medical appointments for hypertension care (i.e., *offer*). In addition, we presented the gap estimates in relation to hypertension treatment cascade metrics: diagnosed, treated and controlled. Further details about the data sources and analysis are shown in Supplementary Materials; analysis code is available as Supplementary Materials.

Data sources

The prevalence of hypertension in each country amongst people aged 30-79 years was retrieved from the Non-Communicable Disease Risk Factor Collaboration (NCD-RisC);[4, 7] the data are publically available online.[7] From this data source we also extracted the proportion diagnosed, treated and controlled amongst people with hypertension (i.e., hypertension care cascade).[4] The NCD-RisC estimates are sex-stratified. In here, we reported one estimate per country (i.e., men and women combined). For this, we multiplied the sex-specific prevalence by the number of men and women, and summed these products to have the country prevalence.

The number of physicians in each country was retrieved from the World Health Organization.[8] Population data (number of people per country) were retrieved from the Institute for Health Metrics and Evaluation (IHME).[9] The world region and income group for each country was retrieved from the World Bank.[10]

The hypertension data (source: NCD-RisC[4, 7]) is available for all years until 2019, whereas the number of physicians for each country is available at different years (i.e., some countries have more recent data than others). We combined the number of physicians with the year-corresponding hypertension data (e.g., number of physician data in 2017 with hypertension data in 2017); population data was for the same year. We only used the most recent World Bank region and income classification (regardless of the physician or hypertension data year).[10]

Variables

In here, *demand* refers to the number of medical appointments needed by people with hypertension. As in a previous paper,[6] we assumed three scenarios: three, six and 12 medical appointments by year. We multiplied the absolute number of people with hypertension times three, six and 12 to compute the number of medical appointments needed. In addition to these three scenarios, we considered one through 12 medical appointments per year; that is, if people with hypertension would need from one to 12 medical appointments per year.

For peer review only - http://bmjopen.bmj.com/site/about/guidelines.xhtml

In here, *offer* refers to the response of the health system to the appointments needed (*demand*) by people with hypertension. We assumed that a physician could see 25 patients per day and he/she works 200 days per year (25 x 200 = 5,000); we multiplied the number of physicians times 5,000 to compute the number of medical appointments available. This product was further multiplied by 0.10 because we assumed that 10% of a physician's time is dedicated to hypertension care.[6]

In here, *the gap* refers to the difference between the *offer* and the *demand*. That is, we subtracted the absolute number of medical appointments needed (*demand*) from the absolute number of medical appointments available (*offer*), for the three scenarios (three, six and 12 medical appointments) and from one through 12 medical appointments per year. These subtractions were further divided by 1,000,000 so that the *gap* is reported per 1,000,000 medical appointments. A negative gap suggests that the *demand* exceeded the *offer*; that is, there were more medical appointments needed than available for hypertension care.[6]

Statistical analysis

Results are presented as counts (absolute numbers), as proportions or percentages, as well as medians. Heatmaps and line plots are used to show at what number of medical appointments needed the gap turned negative. We used world maps to show countries with a negative gap according to the three scenarios (three, six and 12 medical appointments). We used boxplots to depict the mean of the hypertension care cascade metrics (diagnosed, treated and controlled) by the three scenarios amongst countries with positive and negative gaps. We used scatter plots to correlate the physician density (absolute number of physicians per 1,000 people) and the hypertension care cascade metrics; these plots had the Pearson correlation coefficients.

Ethics

We used data available in the public domain and at the country level. The data did not contain any personal identifiers. We did not seek approval by an ethics committee. The opinions herein expressed are those of the authors alone. The funder had no role in the study design, analysis, results interpretation or conclusions. All authors approved the submitted version.

For peer review only - http://bmjopen.bmj.com/site/about/guidelines.xhtml

Patient and Public Involvement

It was not appropriate or possible to involve patients or the public in the design, or conduct, or reporting, or dissemination plans of our research

RESULTS

Data characteristics

There were 191 countries: 30 in East Asia and Pacific, 49 in Europe and Central Asia, 33 in Latin America and the Caribbean, 20 in Middle East and North Africa, 2 in North America, 8 in South Asia and 47 in Sub-Saharan Africa; of note, two countries were not classified into any of these groups: Cook Islands and Nauru. Fifty-eight countries were high income, 54 were upper-middle-income, 49 were lower-middle income, and 28 were low income; of note, two countries (Cook Islands and Nauru) were not classified into any of these groups. Data regarding the number of physicians were available from 2004 to 2019, with 85% available since 2015.

Underlying variables

The prevalence of hypertension ranged from 20% to 56%; the median across all countries was 37%; the median by region was 42% in Europe and Central Asia, 40% in Latin America and the Caribbean, 36% in South Asia, 35% in East Asia and Pacific, 34% in Sub-Saharan Africa, 33% in Middle East and North Africa, and 32% in North America.

The density of physicians (per 1,000 people) ranged from 0.07 to 13.77 (median = 3.26); the median physician density (per 1,000 people) according to the region was 0.36 in Sub-Saharan Africa, 1.75 in East Asia and Pacific, 1.99 in South Asia, 3.67 in Middle East and North Africa, 3.84 in Latin America and the Caribbean, 4.34 in North America, and 6.09 in Europe and Central Asia.

Gap: available (*offer*) medical appointments minus demand

Out of 191 countries in the analysis, 41 countries would have a negative gap to provide one medical appointment per year for people with hypertension (Figure 1); that is, in 41 countries, and under the assumptions of our analysis, the healthcare system would not have enough physicians for all patients with hypertension to have at least one medical appointment per year.

For peer review only - http://bmjopen.bmj.com/site/about/guidelines.xhtml

For peer review only

According to their income, these 41 countries were either low income (21 countries) or lower-middle income (19 countries); one country (Botswana) was upper-middle income. According to their location, these 41 countries were in Sub-Saharan Africa (35 countries; largest gap in DR Congo), East Asia and Pacific (4 countries; largest gap in Papua New Guinea), Middle East and North Africa (1 country; Djibouti) and Latin America and the Caribbean (1 country; Haiti).

The number of countries with a negative gap (more *demand* than *offer*) increased with the number of medical appointments per year (Figure 2). Africa had the largest proportion of countries with a negative gap regardless of the number of medical appointments. Regardless of the world region, ≥50% of the countries could not offer eight or more medical appointments per year to the population with hypertension.

Gap: available (*offer*) medical appointments minus demand in fixed scenarios

For a fixed number of three medical appointments (i.e., every four months) per year, 80 (42%) countries would have a negative gap (more *demand* than *offer*); 24 of these countries were low income whereas two were high income (Figure 3). For a fixed number of six medical appointments (i.e., twice per year) per year, 132 (69%) countries would have a negative gap; 27 of these countries were low income whilst 23 were high income. For a fixed number of 12 medical appointments (i.e., monthly) per year, 179 (94%) countries would have a negative gap; 28 of these countries were low income while 50 were high income.

Gap: available medical appointments minus demand in fixed scenarios and in relation to hypertension diagnosis, treatment, and control

After stratifying the number of visits according to the three scenarios (three, six and 12 medical appointments per year), those countries with a negative gap (more *demand* than *offer*) had worse hypertension treatment cascade metrics than those countries without a negative gap (Figure 4). For example, across countries with always a negative gap, the median prevalence of diagnosed hypertension was 40% versus a median prevalence of 63% across countries without a negative gap; the corresponding estimates for hypertension treatment were 23% versus 52%, and for hypertension control were 9% versus 23%. That is, in countries where the *demand* exceeded the *offer*, the hypertension treatment cascade metrics were worse (on average) than in countries

For peer review only - http://bmjopen.bmj.com/site/about/guidelines.xhtml

where the *demand* did not exceed the *offer* of medical appointments for hypertension care per year.

Correlation between hypertension diagnosis, treatment and control with physician density

For all three hypertension care cascade metrics, there was a positive correlation with the physician density (Figure 5). The magnitude of the correlation was stronger for hypertension diagnosis and treatment than for hypertension control. Low income countries were most often found in the bottom left quadrant, with worse hypertension care cascade metrics and lower physician density. Conversely, high income countries were often found in the upper right quadrant, with better hypertension care cascade metrics and higher physician density.

DISCUSSION

Leveraging on high-quality and official data sources[7, 9, 10] and building on previous research,[6] we documented that 41 countries would not have enough physicians to provide at least one medical appointment per year to their population with hypertension; these countries were either low or lower-middle income and located in Sub-Saharan Africa or in East Asia and Pacific. Regardless of the world region, more than half of all countries would not have enough physicians to provide eight or more medical appointments to their population with hypertension per year. On average, countries where the *demand* exceeded the *offer* of medical appointments for people with hypertension also had worse hypertension care cascade metrics than those countries where the *demand* did not exceed the *offer*. There were positive correlations between the density of physicians and hypertension diagnosis, treatment and control rates.

We followed the methodology developed by the authors of the only global work on this subject.[6] Despite minor changes in our methodology (e.g., updated data sources) which led to different numeric results, we reached similar conclusions:[6] many low- and middle-income countries would struggle to provide medical appointments per year to their population with hypertension. This does not mean that the more medical appoints for hypertension care the better; in other words, quantity does not necessarily reflect quality. We advocate for innovative approaches whereby people with hypertension receive the best evidence-based care available, so that they receive pharmacological and non-pharmacological care to delay or avoid complications.

For peer review only - http://bmjopen.bmj.com/site/about/guidelines.xhtml

Vedanthan *et al.* reported a positive association between physician density and hypertension treatment rates in the unadjusted model only;[5] potential explanations for the lack of statistical significance in adjusted model include data collation year and not having estimates for some countries where there could have been a large gap. We computed correlation coefficients showing a positive correlation between physician density and hypertension treatment; arguably, this shows a similar result to their unadjusted estimates.[5] However, we advanced the literature by showing the correlation between physician density and hypertension diagnosis and control.

We built on previous research which proposed a solid and reasonable methodology to quantify the gap between the *offer* and *demand* of medical appointments for hypertension care at the country level.[6] This work substantially advanced our previous publication by using the most recent hypertension data which is comparable across all countries;[4] by focusing on hypertension (rather than raised blood pressure[6]), which accounts for aware and unaware cases; and by studying the gap between *offer* and *demand* in relation to hypertension care cascade metrics.

Notwithstanding, there are limitations we need to acknowledge. First, all of our results are not for the same year. Potential country-specific users of our results are advised to understand our estimates taking into consideration the data year (Supplementary Table 1). Health organizations should also make efforts to secure consistent, comparable and recent data about the number of physicians in all countries and when possible, stratified by medical speciality. Second, because of hypertension data availability, we restricted our analysis to the age group 30-79 years.[4] Perhaps, older people may require more medical appointments because of multimorbidity and polypharmacy. If so, our results show a conservative scenario and alert that the gap between *offer* and *demand* of medical appointments for hypertension care could be larger if we had included the entire adult population. Third, we used the number of physicians as reported by the World Health Organization, and assumed each physician sees 25 patients per day and works 200 days per year.[6] Although conservative and possible, we are not aware of empirical data to support this (or other) scenario. Any other scenario, either to replace our assumption or as a sensitivity analysis, would also be an educated assumption because of the lack of empirical evidence to inform the scenarios. International health organizations, through surveys or country consultations, could collect data on the actual load of the healthcare workforce and how much time is allocated to key tasks (e.g., non-communicable diseases care). Fourth, country-level estimates often hide

For peer review only - http://bmjopen.bmj.com/site/about/guidelines.xhtml

within country differences. Our results should be interpreted in light of this limitation, and we encourage researchers and health officials to conduct similar analysis at the sub-national level to tailor local policies and interventions. Fifth, the correlations between the gap and the hypertension care cascade metrics were at the country level. This ecological analysis should be cautiously interpreted and need further verification with individual-level data. The most salient limitation of our work is that we assumed only physicians prescribe treatment for hypertension yet other healthcare personnel may participate in this task. Unfortunately, there are no global data to make the distinction on whether all hypertension care is provided by physicians or other personnel. This work alerts of a large gap to meet the needs of people with hypertension, though the gap may be smaller if other healthcare personnel provide treatment for people with hypertension.

The fact that 41 countries would not have enough physicians to provide one medical appointment per year to their population with hypertension is alarming; similarly, having three medical appoints per year (i.e., one every four months) was also unfeasible in many countries.

Task shifting strategies[11, 12] could offer a potential solution for countries where the *demand* exceeded the *offer* of medical appointments for hypertension care. This could include training nurses and other non-physician health professionals,[13-16] as well as community health workers or champions of the community,[16] to monitor blood pressure and to dispense antihypertensive treatment. Physicians could oversee these strategies and focus on difficult cases, such as patients with resistant hypertension or when treatment requires careful tailoring. Having antihypertensive treatment refilled in the local pharmacy could also help.[16, 17] Future research should quantify the impact of task shifting strategies to improve care for people with hypertension.

Countries could adopt prescription strategies that meet their resources. For example, if a country could only have four medical appointments per year for people with hypertension, they could provide medicines for three months. Patients would have enough pills until the next appointment. This would require careful consideration by the physician to identify patients who would not need frequent medical appointments, according to the patient's overall health profile.

There is strong evidence showing the value of non-physician health professionals to improve hypertension care.[11, 12, 16, 17] Selecting the best examples which could be implemented following rigorous research methods and evaluations deserves prompt attention.

For peer review only - http://bmjopen.bmj.com/site/about/guidelines.xhtml

The COVID-19 pandemic would impact our results and the associated implications. First, the latest physician data was for 2019. Unfortunately, many physicians have recently lost their lives to COVID-19,[18] potentially widening the herein documented gaps especially in low- and middle-income countries. Second, COVID-19 disrupted health services because they stopped their regular activities to take care of COVID-19 patients; lockdowns also disrupted regular health services.[19] Therefore, people with chronic diseases including hypertension had their usual care interrupted. This could have led to treatment discontinuation and fewer patients meeting blood pressure targets. As health services resume their regular activities, patients with chronic conditions may need more than usual care to catch up with their treatments. This would translate into more frequent medical appointments widening the gap herein documented.

Brain drain of physicians could also play a relevant role.[20] Physicians from low- and middle-income countries, where we found the largest gaps, tend to migrate to high-income countries. If this continues, the herein document gaps in low- and middle-income countries would increase.

Both of these potential drivers could be solved with more physicians, though this may not be a realistic option. To support timely and continues treatment for people with hypertension where physicians are the only health professional authorized to prescribe medicines, it seems imperative to find solutions that does not require physicians only.

Many low and middle-income countries would not meet the *demand* of medical appointments needed by people with hypertension. This would lead to lower rates of hypertension treatment and control at the country level. Because hypertension is the leading risk factor for cardiovascular morbidity and mortality,[1] and has poor treatment and control rates in many low- and middle-income countries,[4] healthcare systems should secure better care for people with hypertension including the uninterrupted provision of antihypertensive medication. A synergic participation of physicians, non-physician healthcare workers and lay people (e.g., community health workers) would be needed to secure continuous treatment for (all) people with hypertension.

For peer review only - http://bmjopen.bmj.com/site/about/guidelines.xhtml

Acknowledgements

None.

Funding

RMC-L is supported by a Wellcome Trust International Training Fellowship (Wellcome Trust 214185/Z/18/Z). DN receives support from Resolve to Save Lives, an initiative of Vital Strategies, which is funded by Bloomberg Philanthropies, the Bill & Melinda Gates Foundation, and Gates Philanthropy Partners, which is funded with support from the Chan Zuckerberg Initiative.

Conflict of interest

None to declare.

Contributions

All authors conceived the study. WCGV collated, pooled and conducted the analysis. RMC-L conducted the analysis and drafted the first version. All authors discussed the findings and provided critical input to the manuscript. All authors approved the submitted version.

Data availability

All data sources herein analysed are in the public domain, and links to these sources have been provided in the main manuscript. We are also providing the analysis data (i.e., after pooling individual data sources) and the analysis code in R.

For peer review only - http://bmjopen.bmj.com/site/about/guidelines.xhtml

For peer review only

FIGURES

Figure 1. Indicator of positive or negative gap by number of medical appointments (1 through 12) per year by country.

A negative gap indicates that there is more demand (need of medical appointment by people with hypertension) than offer (number of physicians).

Figure 2. Proportion of countries with a negative gap by number of medical appointments (1 through 12) per year by world region.

Two countries (Cook Islands and Nauru) were not included in the plot because their region was not available in the data source (World Bank).

Figure 3. World maps showing countries according to the gap level by the three possible scenarios, and signalling which countries would always have a negative gap regardless of the scenario.

Figure 4. Hypertension treatment cascade metrics by positive or negative gap stratified by the three possible scenarios.

Each dot represents a country.

Figure 5. Correlation between hypertension treatment cascade metrics and physician density.

Each dot is a country; colours indicate the income group of each country.

For peer review only - http://bmjopen.bmj.com/site/about/guidelines.xhtml

REFERENCES

1. GBD 2019 Risk Factors Collaborators. Global burden of 87 risk factors in 204 countries and territories, 1990-2019: a systematic analysis for the Global Burden of Disease Study 2019. *Lancet (London, England)* 2020; **396**(10258): 1223-49.

2. Zhou B, Perel P, Mensah GA, Ezzati M. Global epidemiology, health burden and effective interventions for elevated blood pressure and hypertension. *Nature reviews Cardiology* 2021: 1-18.

3. Attaei MW, Khatib R, McKee M, et al. Availability and affordability of blood pressure-lowering medicines and the effect on blood pressure control in high-income, middle-income, and low-income countries: an analysis of the PURE study data. *The Lancet Public health* 2017; **2**(9): e411-e9.

4. NCD Risk Factor Collaboration (NCD-RisC). Worldwide trends in hypertension prevalence and progress in treatment and control from 1990 to 2019: a pooled analysis of 1201 population-representative studies with 104 million participants. *Lancet (London, England)* 2021; **398**(10304): 957-80.

5. Vedanthan R, Ray M, Fuster V, Magenheim E. Hypertension Treatment Rates and Health Care Worker Density. *Hypertension (Dallas, Tex : 1979)* 2019; **73**(3): 594-601.

6. Neupane D, Gao Y, Feng Y, Matsushita K, Appel LJ. Estimation of the Global Gap in Clinic Visits for Hypertension Care Between Patient Need and Physician Capacity. *Hypertension (Dallas, Tex : 1979)* 2021; **78**(3): 779-86.

7. Non-Communicable Diseases Risk Factor Collaboration (NCD-RisC). [cited 2021 Sept 23]. Available from: https://ncdrisc.org/.

8. The Global Health Observatory - Medical doctors (number). [cited 2021 Sep 23]. Available from: https://www.who.int/data/gho/data/indicators/indicator-details/GHO/medical-doctors-(number).

9. Global Burden of Disease Study 2019 (GBD 2019) Population Estimates 1950-2019. [cited 2021 Sep 23]. Available from: http://ghdx.healthdata.org/record/ihme-data/gbd-2019-population-estimates-1950-2019.

For peer review only

For peer review only - http://bmjopen.bmj.com/site/about/guidelines.xhtml

10. World Bank Country and Leading Groups. [cited 2021 Sep 23]. Available from: https://datahelpdesk.worldbank.org/knowledgebase/articles/906519-world-bank-country-and-lending-groups.

11. Joshi R, Alim M, Kengne AP, et al. Task shifting for non-communicable disease management in low and middle income countries--a systematic review. *PloS one* 2014; **9**(8): e103754.

12. Ogedegbe G, Gyamfi J, Plange-Rhule J, et al. Task shifting interventions for cardiovascular risk reduction in low-income and middle-income countries: a systematic review of randomised controlled trials. *BMJ open* 2014; **4**(10): e005983.

13. Kavita, Thakur JS, Vijayvergiya R, Ghai S. Task shifting of cardiovascular risk assessment and communication by nurses for primary and secondary prevention of cardiovascular diseases in a tertiary health care setting of Northern India. *BMC health services research* 2020; **20**(1): 10.

14. Ogedegbe G, Plange-Rhule J, Gyamfi J, et al. Health insurance coverage with or without a nurse-led task shifting strategy for hypertension control: A pragmatic cluster randomized trial in Ghana. *PLoS medicine* 2018; **15**(5): e1002561.

15. Schwalm JD, McCready T, Lopez-Jaramillo P, et al. A community-based comprehensive intervention to reduce cardiovascular risk in hypertension (HOPE 4): a cluster-randomised controlled trial. *Lancet (London, England)* 2019; **394**(10205): 1231-42.

16. Anand TN, Joseph LM, Geetha AV, Prabhakaran D, Jeemon P. Task sharing with non-physician health-care workers for management of blood pressure in low-income and middle-income countries: a systematic review and meta-analysis. *The Lancet Global health* 2019; **7**(6): e761-e71.

17. Wagner TD, Jones MC, Salgado TM, Dixon DL. Pharmacist's role in hypertension management: a review of key randomized controlled trials. *Journal of human hypertension* 2020; **34**(7): 487-94.

18. Gouda D, Singh PM, Gouda P, Goudra B. An Overview of Health Care Worker Reported Deaths During the COVID-19 Pandemic. *Journal of the American Board of Family Medicine : JABFM* 2021; **34**(Suppl): S244-s6.

For peer review only - http://bmjopen.bmj.com/site/about/guidelines.xhtml

19. Skeete J, Connell K, Ordunez P, DiPette DJ. Approaches to the Management of Hypertension in Resource-Limited Settings: Strategies to Overcome the Hypertension Crisis in the Post-COVID Era. *Integrated blood pressure control* 2020; **13**: 125-33.

20. Botezat A, Ramos R. Physicians' brain drain - a gravity model of migration flows. *Globalization and Health* 2020; **16**(1): 7.

For peer review only

For peer review only - http://bmjopen.bmj.com/site/about/guidelines.xhtml

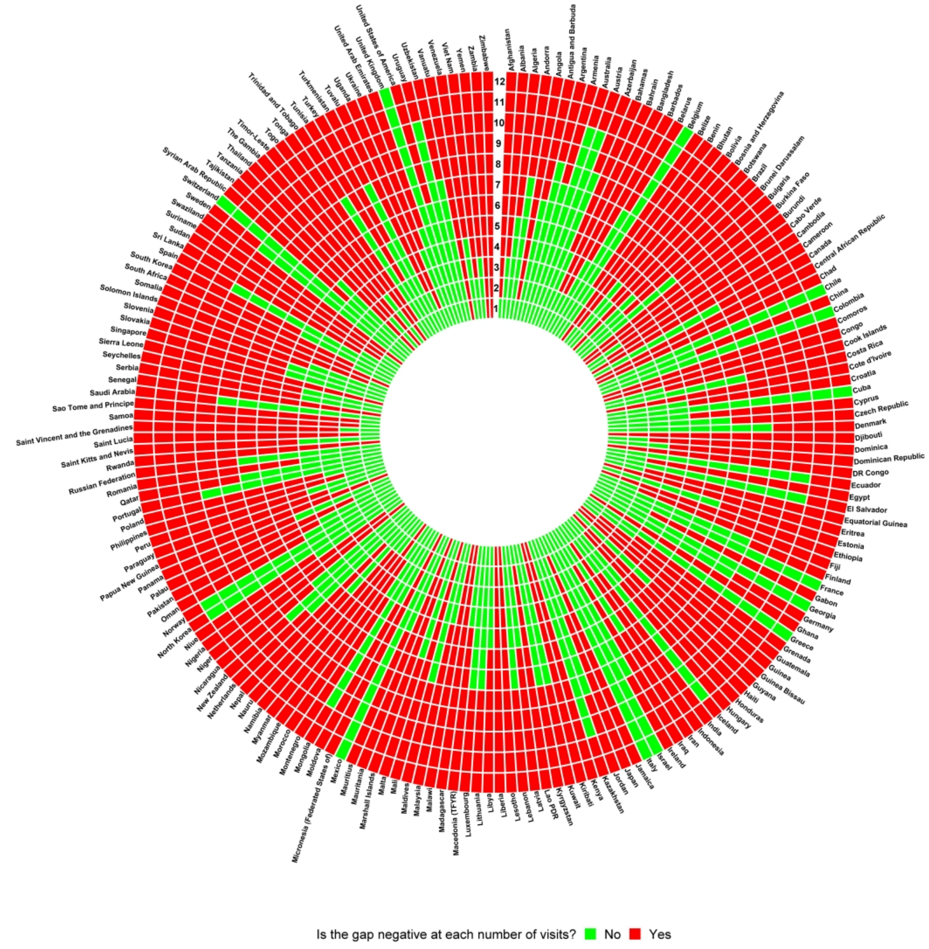

1693x1693mm (72 x 72 DPI)

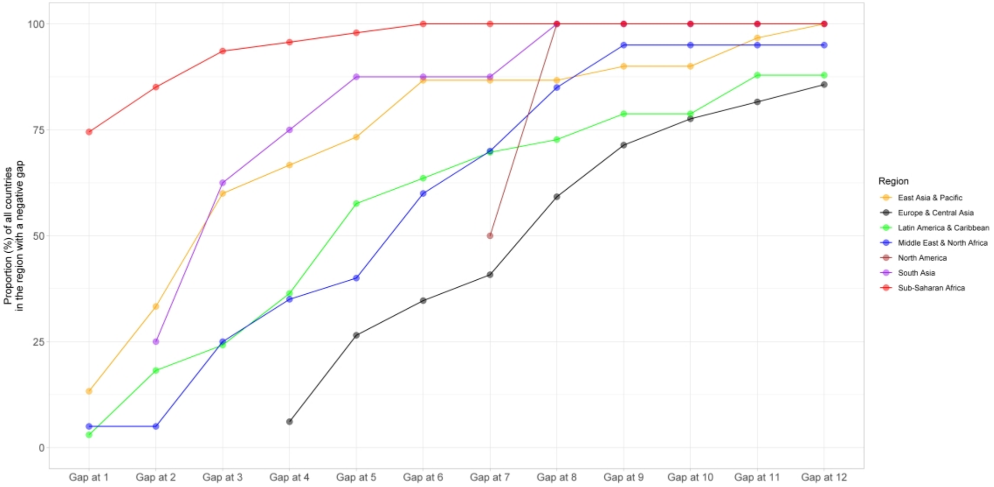

1693x846mm (72 x 72 DPI)

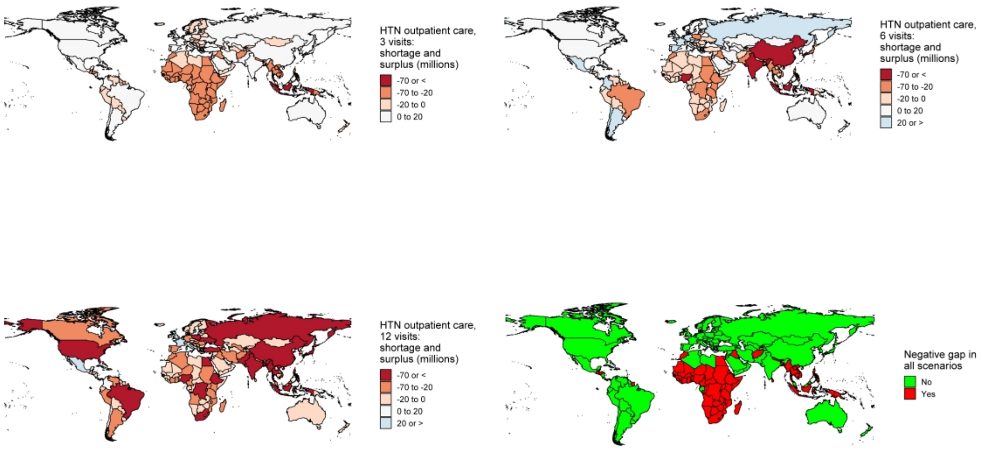

2116x1270mm (72 x 72 DPI)

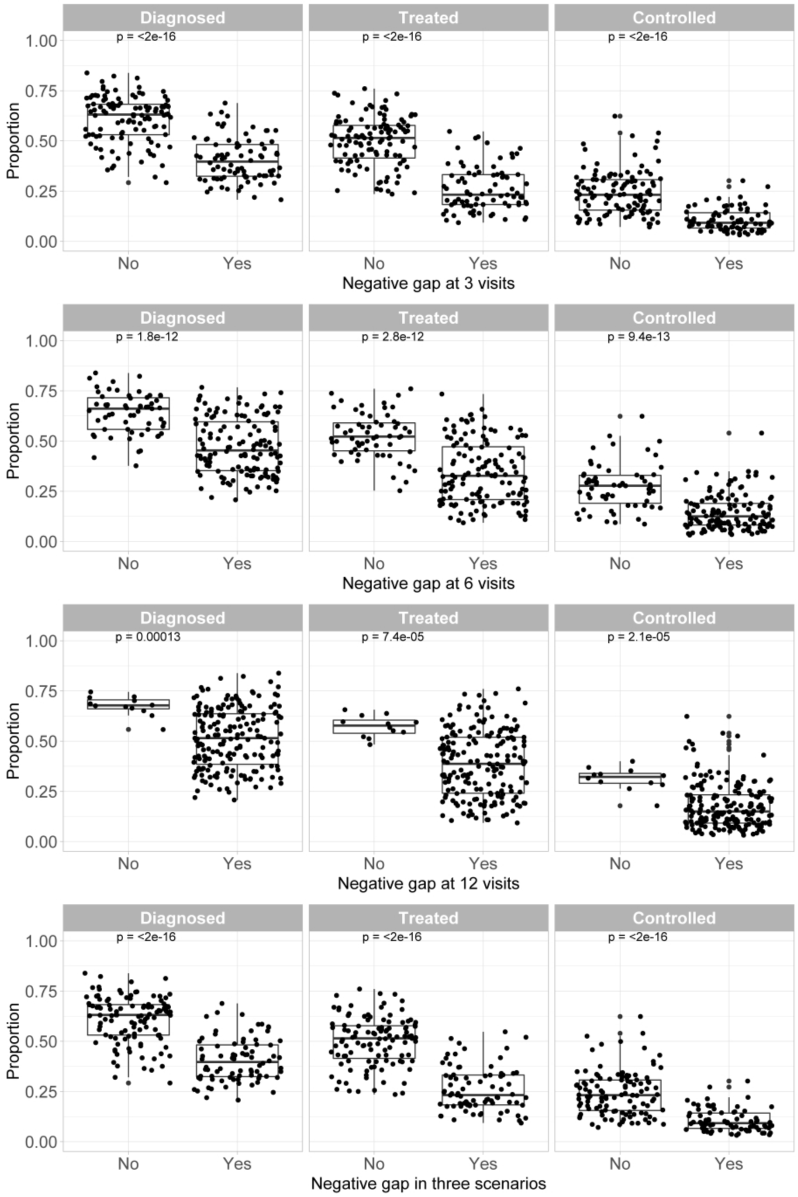

1587x2381mm (72 x 72 DPI)

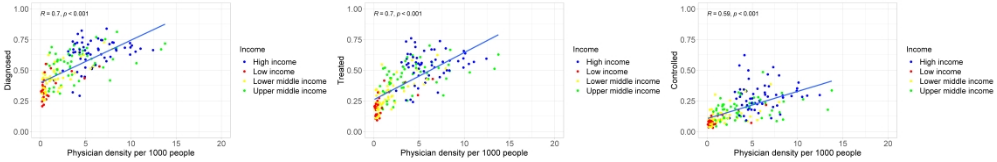

1614x258mm (236 x 236 DPI)

SUPPLEMENTARY MATERIAL

Gap between demand and offer of medical appointments for hypertension care: A global analysis

Corresponding Author

Rodrigo M Carrillo-Larco, MD

Department of Epidemiology and Biostatistics, School of Public Health, Imperial College London, London, UK.

r.carrillo-larco@imperial.ac.uk

For peer review only

EXPANDED METHODS ... 3

Data sources ... 3

Data management ... 4

New variables ... 4

References ... 6

Supplementary Table 1. Gap (expressed in 1,000,000 medical appointments) between offer of medical appointments and demand of medical appointments by people with hypertension per year, from one through twelve medical appointments per year. ... 7

For peer review only

For peer review only - http://bmjopen.bmj.com/site/about/guidelines.xhtml

EXPANDED METHODS

All analysis codes are available as Supplementary Materials.

Data sources

We combined multiple data sources.

- Hypertension[1, 2] data from the Non-Communicable Disease Risk Factor Collaboration (NCD-RisC). These data included prevalence of hypertension, and amongst people with hypertension, these data also provided the proportion of diagnosed (i.e., awareness), treated and controlled. These data are for all countries and for all years until 2019. All these hypertension estimates are sex-stratified and for the age group 30-79 years.
- The number of physicians[3] was retrieved from the World Health Organization (WHO) observatory. These data are for all countries, though the latest available data may be different by country (i.e., not all countries have data for the same years). The most recent data was for the year 2019 (not all countries have data until 2019).

Year	Number of countries with data	Proportion of all data
2004	1	0.005
2008	1	0.005
2009	1	0.005
2010	1	0.005
2011	1	0.005
2012	3	0.016
2013	3	0.016
2014	16	0.084
2015	14	0.073
2016	17	0.089
2017	32	0.168
2018	60	0.314
2019	41	0.215

For peer review only - http://bmjopen.bmj.com/site/about/guidelines.xhtml

- Population[4] data (number of inhabitants per country) was retrieved from the Institute for Health Metrics and Evaluation (IHME). We extracted the number of men and women in the age group 30-79 for all years until 2019.
- The World Bank classification[5] was used to label countries according to income (low, middle and high income) and world region. We only used the latest available data.

Data management

We downloaded each data source. In the dataset for number of physicians we only kept the most recent year per country. The hypertension data and the number of physicians data were merged matching by year and country; in other words, both data sources belonged to the same year. These data were then merged with population data matching by year, country and sex (because hypertension data were sex-stratified). Matching by country, we then merged with the World Bank data; the World Bank data would not necessarily be synchronic with the other data. Overall, hypertension, number of physicians and population data was synchronic (belonged to the same year).

New variables

The sex-specific prevalence of hypertension was multiplied by the number of men and women in each country, to compute the absolute number of men and women with hypertension. These sex-specific absolute numbers of people with hypertension were then multiplied by the proportion of diagnosed, treated and controlled; this, to compute the absolute number of people with diagnosed, treated and controlled hypertension. Because all these estimates were sex-specific, we summed them within each country to have one estimate per country (i.e., overall by country rather than sex-stratified). For diagnosis, treatment and control rates, we followed a similar procedure to move from sex-stratified estimates to one-only per country.

We computed the number of medical appointments needed by people with hypertension, hereafter referred to as *demand*. We multiplied the absolute number of people with hypertension by [1…6…12]. If people with hypertension would need 1 medical appointment per year, then there would be as many medical appointment as people with hypertension (multiplication by 1). On the other hand, if people with hypertension would need 12 medical appointments per year, then there

For peer review only - http://bmjopen.bmj.com/site/about/guidelines.xhtml

would be as many medical appointments as 12 times the number of people with hypertension (multiplication by 12). The same logic would apply for all natural numbers between 1 and 12.

We computed the number of medical appointments available for people with hypertension, hereafter referred to as *offer*. For this, we multiplied the number of physicians by 5,000; this factor was conceived assuming that a physician could see 25 patients per day and work 200 days per year (25 x 200 = 5,000). The product between the number of physicians and 5,000 was then multiplied by 0.10, because we assumed that 10% of their time was dedicated to hypertension care.

We computed the gap between the *offer* and *demand* (*offer* - *demand*), as a simple subtraction between the number of medical appointments available (*offer*) and the number of medical appointments needed by people with hypertension (*demand*). We did this subtraction 12 times, for each of the potential number of medical appointments per year (1 through 12, as described in the second paragraph of this section above).

A negative gap would mean that the *demand* exceeded the *offer*. In other words, the healthcare system would not have enough physicians to meet the needs of medical appointments per year for all patients with hypertension.

For peer review only

References

1. Non-Communicable Diseases Risk Factor Collaboration (NCD-RisC). [cited 2021 Sept 23]. Available from: https://ncdrisc.org/.

2. NCD Risk Factor Collaboration (NCD-RisC). Worldwide trends in hypertension prevalence and progress in treatment and control from 1990 to 2019: a pooled analysis of 1201 population-representative studies with 104 million participants. *Lancet (London, England)* 2021; **398**(10304): 957-80.

3. The Global Health Observatory - Medical doctors (number). [cited 2021 Sep 23]. Available from: https://www.who.int/data/gho/data/indicators/indicator-details/GHO/medical-doctors-(number).

4. Global Burden of Disease Study 2019 (GBD 2019) Population Estimates 1950-2019. [cited 2021 Sep 23]. Available from: http://ghdx.healthdata.org/record/ihme-data/gbd-2019-population-estimates-1950-2019.

5. World Bank Country and Leading Groups. [cited 2021 Sep 23]. Available from: https://datahelpdesk.worldbank.org/knowledgebase/articles/906519-world-bank-country-and-lending-groups.

For peer review only

For peer review only - http://bmjopen.bmj.com/site/about/guidelines.xhtml

Supplementary Table 1. Gap (expressed in 1,000,000 medical appointments) between offer of medical appointments and demand of medical appointments by people with hypertension per year, from one through twelve medical appointments per year.

Country	Year	Number medical appointment	Gap
Afghanistan	2016	Gap 1 visit per year	1.828452
Albania	2019	Gap 1 visit per year	1.647525
Algeria	2018	Gap 1 visit per year	29.86253
Andorra	2015	Gap 1 visit per year	0.112669
Angola	2017	Gap 1 visit per year	0.580218
Antigua and Barbuda	2017	Gap 1 visit per year	0.111459
Argentina	2017	Gap 1 visit per year	77.132914
Armenia	2017	Gap 1 visit per year	5.648676
Australia	2018	Gap 1 visit per year	42.152433
Austria	2018	Gap 1 visit per year	20.915018
Azerbaijan	2014	Gap 1 visit per year	14.534057
Bahamas	2017	Gap 1 visit per year	0.284629
Bahrain	2015	Gap 1 visit per year	0.37155
Bangladesh	2019	Gap 1 visit per year	32.989788
Barbados	2017	Gap 1 visit per year	0.274082
Belarus	2015	Gap 1 visit per year	21.331173
Belgium	2019	Gap 1 visit per year	31.886496
Belize	2018	Gap 1 visit per year	0.151593
Benin	2019	Gap 1 visit per year	-0.561566
Bhutan	2019	Gap 1 visit per year	0.041024
Bolivia	2017	Gap 1 visit per year	4.531482
Bosnia and Herzegovina	2015	Gap 1 visit per year	2.609187
Botswana	2018	Gap 1 visit per year	-0.058452
Brazil	2019	Gap 1 visit per year	192.521573
Brunei Darussalam	2017	Gap 1 visit per year	0.251274
Bulgaria	2018	Gap 1 visit per year	12.430232
Burkina Faso	2019	Gap 1 visit per year	-0.722325
Burundi	2017	Gap 1 visit per year	-0.375562
Cabo Verde	2015	Gap 1 visit per year	0.118139
Cambodia	2014	Gap 1 visit per year	0.186672
Cameroon	2011	Gap 1 visit per year	-1.040146
Canada	2019	Gap 1 visit per year	39.573208
Central African Republic	2015	Gap 1 visit per year	-0.373227
Chad	2019	Gap 1 visit per year	-0.856829
Chile	2018	Gap 1 visit per year	44.745072
China	2017	Gap 1 visit per year	1159.935565
Colombia	2018	Gap 1 visit per year	88.245797

For peer review only - http://bmjopen.bmj.com/site/about/guidelines.xhtml

Comoros	2012	Gap 1 visit per year	-0.011996
Congo	2016	Gap 1 visit per year	-0.345107
Cook Islands	2014	Gap 1 visit per year	0.008377
Costa Rica	2018	Gap 1 visit per year	6.325041
Cote d'Ivoire	2019	Gap 1 visit per year	-0.675864
Croatia	2016	Gap 1 visit per year	4.807949
Cuba	2018	Gap 1 visit per year	44.676305
Cyprus	2016	Gap 1 visit per year	0.884493
Czech Republic	2019	Gap 1 visit per year	18.685206
DR Congo	2013	Gap 1 visit per year	-3.00928
Denmark	2018	Gap 1 visit per year	10.652162
Djibouti	2014	Gap 1 visit per year	-0.022054
Dominica	2018	Gap 1 visit per year	0.021846
Dominican Republic	2019	Gap 1 visit per year	5.478795
Ecuador	2017	Gap 1 visit per year	16.775124
Egypt	2019	Gap 1 visit per year	23.325701
El Salvador	2018	Gap 1 visit per year	8.353597
Equatorial Guinea	2017	Gap 1 visit per year	0.143317
Eritrea	2004	Gap 1 visit per year	-0.207366
Estonia	2017	Gap 1 visit per year	1.918452
Ethiopia	2018	Gap 1 visit per year	-2.943908
Fiji	2015	Gap 1 visit per year	0.230687
Finland	2018	Gap 1 visit per year	11.317765
France	2018	Gap 1 visit per year	198.165051
Gabon	2017	Gap 1 visit per year	0.500059
Georgia	2019	Gap 1 visit per year	13.104753
Germany	2018	Gap 1 visit per year	158.387529
Ghana	2019	Gap 1 visit per year	-1.741726
Greece	2018	Gap 1 visit per year	30.243184
Grenada	2018	Gap 1 visit per year	0.055392
Guatemala	2018	Gap 1 visit per year	1.311459
Guinea	2016	Gap 1 visit per year	-0.715036
Guinea Bissau	2016	Gap 1 visit per year	-0.054769
Guyana	2018	Gap 1 visit per year	0.576234
Haiti	2018	Gap 1 visit per year	-0.490878
Honduras	2017	Gap 1 visit per year	0.474619
Hungary	2018	Gap 1 visit per year	13.137566
Iceland	2019	Gap 1 visit per year	0.638479
India	2019	Gap 1 visit per year	448.510152
Indonesia	2019	Gap 1 visit per year	13.12214
Iran	2018	Gap 1 visit per year	54.631301
Iraq	2018	Gap 1 visit per year	7.303598
Ireland	2019	Gap 1 visit per year	7.183227
Israel	2019	Gap 1 visit per year	21.870098
Italy	2019	Gap 1 visit per year	226.051799

For peer review only - http://bmjopen.bmj.com/site/about/guidelines.xhtml

For peer review only

Jamaica	2016	Gap 1 visit per year	0.068221
Japan	2018	Gap 1 visit per year	124.425971
Jordan	2017	Gap 1 visit per year	10.029233
Kazakhstan	2014	Gap 1 visit per year	31.023502
Kenya	2018	Gap 1 visit per year	-0.402478
Kiribati	2013	Gap 1 visit per year	-0.002621
Kuwait	2015	Gap 1 visit per year	4.409757
Kyrgyzstan	2014	Gap 1 visit per year	5.597887
Lao PDR	2017	Gap 1 visit per year	0.621481
Latvia	2018	Gap 1 visit per year	2.569005
Lebanon	2018	Gap 1 visit per year	6.306692
Lesotho	2010	Gap 1 visit per year	-0.18446
Liberia	2015	Gap 1 visit per year	-0.405862
Libya	2017	Gap 1 visit per year	5.668735
Lithuania	2019	Gap 1 visit per year	6.003088
Luxembourg	2017	Gap 1 visit per year	0.765244
Macedonia (TFYR)	2015	Gap 1 visit per year	2.365155
Madagascar	2014	Gap 1 visit per year	-0.281018
Malawi	2018	Gap 1 visit per year	-0.91504
Malaysia	2015	Gap 1 visit per year	18.000037
Maldives	2018	Gap 1 visit per year	0.377925
Mali	2018	Gap 1 visit per year	-0.382266
Malta	2015	Gap 1 visit per year	0.518033
Marshall Islands	2012	Gap 1 visit per year	0.006026
Mauritania	2018	Gap 1 visit per year	-0.002867
Mauritius	2018	Gap 1 visit per year	1.331465
Mexico	2018	Gap 1 visit per year	287.724789
Micronesia (Federated States of)	2009	Gap 1 visit per year	-0.000528
Moldova	2019	Gap 1 visit per year	4.019244
Mongolia	2018	Gap 1 visit per year	5.499934
Montenegro	2018	Gap 1 visit per year	0.68017
Morocco	2017	Gap 1 visit per year	7.262086
Mozambique	2019	Gap 1 visit per year	-1.359652
Myanmar	2019	Gap 1 visit per year	10.606037
Namibia	2017	Gap 1 visit per year	0.377202
Nauru	2015	Gap 1 visit per year	0.005723
Nepal	2019	Gap 1 visit per year	7.277984
Netherlands	2018	Gap 1 visit per year	27.577283
New Zealand	2018	Gap 1 visit per year	7.199367
Nicaragua	2018	Gap 1 visit per year	4.545426
Niger	2016	Gap 1 visit per year	-1.420563
Nigeria	2018	Gap 1 visit per year	18.245549
Niue	2008	Gap 1 visit per year	0.001206
North Korea	2017	Gap 1 visit per year	42.819763
Norway	2019	Gap 1 visit per year	11.995948

For peer review only

Oman	2019	Gap 1 visit per year	3.988676
Pakistan	2019	Gap 1 visit per year	91.145146
Palau	2014	Gap 1 visit per year	0.008051
Panama	2019	Gap 1 visit per year	2.779373
Papua New Guinea	2019	Gap 1 visit per year	-0.574706
Paraguay	2018	Gap 1 visit per year	3.141787
Peru	2018	Gap 1 visit per year	10.049479
Philippines	2017	Gap 1 visit per year	17.790306
Poland	2017	Gap 1 visit per year	32.338857
Portugal	2018	Gap 1 visit per year	24.46577
Qatar	2018	Gap 1 visit per year	2.986651
Romania	2017	Gap 1 visit per year	22.700743
Russian Federation	2018	Gap 1 visit per year	279.82376
Rwanda	2019	Gap 1 visit per year	-0.356469
Saint Kitts and Nevis	2018	Gap 1 visit per year	0.057905
Saint Lucia	2017	Gap 1 visit per year	0.020162
Saint Vincent and the Grenadines	2012	Gap 1 visit per year	0.029732
Samoa	2016	Gap 1 visit per year	0.006922
Sao Tome and Principe	2015	Gap 1 visit per year	0.005724
Saudi Arabia	2018	Gap 1 visit per year	38.968292
Senegal	2019	Gap 1 visit per year	-0.961246
Serbia	2016	Gap 1 visit per year	10.957077
Seychelles	2018	Gap 1 visit per year	0.095142
Sierra Leone	2018	Gap 1 visit per year	-0.593629
Singapore	2016	Gap 1 visit per year	5.260476
Slovakia	2018	Gap 1 visit per year	7.958753
Slovenia	2018	Gap 1 visit per year	2.605381
Solomon Islands	2016	Gap 1 visit per year	0.010102
Somalia	2014	Gap 1 visit per year	-1.372145
South Africa	2019	Gap 1 visit per year	12.650215
South Korea	2018	Gap 1 visit per year	50.972288
Spain	2018	Gap 1 visit per year	84.352391
Sri Lanka	2019	Gap 1 visit per year	7.93756
Sudan	2017	Gap 1 visit per year	0.673453
Suriname	2018	Gap 1 visit per year	0.116125
Swaziland	2016	Gap 1 visit per year	-0.086304
Sweden	2017	Gap 1 visit per year	19.17131
Switzerland	2018	Gap 1 visit per year	16.959064
Syrian Arab Republic	2016	Gap 1 visit per year	8.841597
Tajikistan	2014	Gap 1 visit per year	5.889196
Tanzania	2014	Gap 1 visit per year	-2.898421
Thailand	2019	Gap 1 visit per year	18.001516
The Gambia	2019	Gap 1 visit per year	-0.087449
Timor-Leste	2019	Gap 1 visit per year	0.351507
Togo	2019	Gap 1 visit per year	-0.528087

For peer review only - http://bmjopen.bmj.com/site/about/guidelines.xhtml

Tonga	2013	Gap 1 visit per year	0.013223
Trinidad and Tobago	2019	Gap 1 visit per year	2.768295
Tunisia	2017	Gap 1 visit per year	5.403192
Turkey	2018	Gap 1 visit per year	60.335343
Turkmenistan	2014	Gap 1 visit per year	5.367995
Tuvalu	2014	Gap 1 visit per year	0.00279
Uganda	2017	Gap 1 visit per year	0.703368
Ukraine	2014	Gap 1 visit per year	53.160334
United Arab Emirates	2018	Gap 1 visit per year	10.014865
United Kingdom	2019	Gap 1 visit per year	184.019574
United States of America	2018	Gap 1 visit per year	357.101853
Uruguay	2017	Gap 1 visit per year	7.645388
Uzbekistan	2014	Gap 1 visit per year	31.284773
Vanuatu	2016	Gap 1 visit per year	-0.011021
Venezuela	2017	Gap 1 visit per year	20.089373
Viet Nam	2016	Gap 1 visit per year	25.724354
Yemen	2014	Gap 1 visit per year	4.755468
Zambia	2016	Gap 1 visit per year	-0.514945
Zimbabwe	2018	Gap 1 visit per year	-0.219587
Afghanistan	2016	Gap 2 visit per year	-1.264096
Albania	2019	Gap 2 visit per year	0.92255
Algeria	2018	Gap 2 visit per year	23.42306
Andorra	2015	Gap 2 visit per year	0.095338
Angola	2017	Gap 2 visit per year	-2.039564
Antigua and Barbuda	2017	Gap 2 visit per year	0.090918
Argentina	2017	Gap 2 visit per year	66.609328
Armenia	2017	Gap 2 visit per year	4.815352
Australia	2018	Gap 2 visit per year	37.502866
Austria	2018	Gap 2 visit per year	18.661536
Azerbaijan	2014	Gap 2 visit per year	12.690114
Bahamas	2017	Gap 2 visit per year	0.199258
Bahrain	2015	Gap 2 visit per year	0.1081
Bangladesh	2019	Gap 2 visit per year	14.075076
Barbados	2017	Gap 2 visit per year	0.192164
Belarus	2015	Gap 2 visit per year	18.164846
Belgium	2019	Gap 2 visit per year	29.410992
Belize	2018	Gap 2 visit per year	0.096686
Benin	2019	Gap 2 visit per year	-1.504632
Bhutan	2019	Gap 2 visit per year	-0.093452
Bolivia	2017	Gap 2 visit per year	3.298964
Bosnia and Herzegovina	2015	Gap 2 visit per year	1.511874
Botswana	2018	Gap 2 visit per year	-0.442904
Brazil	2019	Gap 2 visit per year	141.213646
Brunei Darussalam	2017	Gap 2 visit per year	0.161048
Bulgaria	2018	Gap 2 visit per year	10.026964

For peer review only

Burkina Faso	2019	Gap 2 visit per year	-2.39965
Burundi	2017	Gap 2 visit per year	-1.293124
Cabo Verde	2015	Gap 2 visit per year	0.031278
Cambodia	2014	Gap 2 visit per year	-1.098656
Cameroon	2011	Gap 2 visit per year	-3.001292
Canada	2019	Gap 2 visit per year	33.458916
Central African Republic	2015	Gap 2 visit per year	-0.908454
Chad	2019	Gap 2 visit per year	-2.146158
Chile	2018	Gap 2 visit per year	40.959144
China	2017	Gap 2 visit per year	905.37163
Colombia	2018	Gap 2 visit per year	81.039594
Comoros	2012	Gap 2 visit per year	-0.085492
Congo	2016	Gap 2 visit per year	-0.955214
Cook Islands	2014	Gap 2 visit per year	0.004254
Costa Rica	2018	Gap 2 visit per year	5.416082
Cote d'Ivoire	2019	Gap 2 visit per year	-3.438228
Croatia	2016	Gap 2 visit per year	3.303898
Cuba	2018	Gap 2 visit per year	41.61961
Cyprus	2016	Gap 2 visit per year	0.627486
Czech Republic	2019	Gap 2 visit per year	15.373412
DR Congo	2013	Gap 2 visit per year	-9.22756
Denmark	2018	Gap 2 visit per year	9.153824
Djibouti	2014	Gap 2 visit per year	-0.144608
Dominica	2018	Gap 2 visit per year	0.004192
Dominican Republic	2019	Gap 2 visit per year	3.16209
Ecuador	2017	Gap 2 visit per year	14.918748
Egypt	2019	Gap 2 visit per year	9.189902
El Salvador	2018	Gap 2 visit per year	7.493694
Equatorial Guinea	2017	Gap 2 visit per year	0.033134
Eritrea	2004	Gap 2 visit per year	-0.522232
Estonia	2017	Gap 2 visit per year	1.552404
Ethiopia	2018	Gap 2 visit per year	-10.085316
Fiji	2015	Gap 2 visit per year	0.087874
Finland	2018	Gap 2 visit per year	9.82203
France	2018	Gap 2 visit per year	183.993102
Gabon	2017	Gap 2 visit per year	0.296118
Georgia	2019	Gap 2 visit per year	12.064006
Germany	2018	Gap 2 visit per year	138.074558
Ghana	2019	Gap 2 visit per year	-5.101452
Greece	2018	Gap 2 visit per year	27.729868
Grenada	2018	Gap 2 visit per year	0.030784
Guatemala	2018	Gap 2 visit per year	-0.438082
Guinea	2016	Gap 2 visit per year	-1.918572
Guinea Bissau	2016	Gap 2 visit per year	-0.223038
Guyana	2018	Gap 2 visit per year	0.443968

For peer review only

Haiti	2018	Gap 2 visit per year	-2.284756
Honduras	2017	Gap 2 visit per year	-0.507262
Hungary	2018	Gap 2 visit per year	9.736132
Iceland	2019	Gap 2 visit per year	0.574958
India	2019	Gap 2 visit per year	262.934304
Indonesia	2019	Gap 2 visit per year	-36.68672
Iran	2018	Gap 2 visit per year	44.460602
Iraq	2018	Gap 2 visit per year	1.003196
Ireland	2019	Gap 2 visit per year	6.183454
Israel	2019	Gap 2 visit per year	20.453696
Italy	2019	Gap 2 visit per year	209.508598
Jamaica	2016	Gap 2 visit per year	-0.524558
Japan	2018	Gap 2 visit per year	91.148942
Jordan	2017	Gap 2 visit per year	8.688966
Kazakhstan	2014	Gap 2 visit per year	27.615004
Kenya	2018	Gap 2 visit per year	-4.825956
Kiribati	2013	Gap 2 visit per year	-0.016242
Kuwait	2015	Gap 2 visit per year	3.744514
Kyrgyzstan	2014	Gap 2 visit per year	4.728774
Lao PDR	2017	Gap 2 visit per year	-0.052538
Latvia	2018	Gap 2 visit per year	1.95451
Lebanon	2018	Gap 2 visit per year	5.397884
Lesotho	2010	Gap 2 visit per year	-0.43792
Liberia	2015	Gap 2 visit per year	-0.895724
Libya	2017	Gap 2 visit per year	4.45897
Lithuania	2019	Gap 2 visit per year	5.052176
Luxembourg	2017	Gap 2 visit per year	0.640488
Macedonia (TFYR)	2015	Gap 2 visit per year	1.74281
Madagascar	2014	Gap 2 visit per year	-2.699536
Malawi	2018	Gap 2 visit per year	-2.15458
Malaysia	2015	Gap 2 visit per year	12.754574
Maldives	2018	Gap 2 visit per year	0.31535
Mali	2018	Gap 2 visit per year	-1.991532
Malta	2015	Gap 2 visit per year	0.416066
Marshall Islands	2012	Gap 2 visit per year	5.20E-05
Mauritania	2018	Gap 2 visit per year	-0.416234
Mauritius	2018	Gap 2 visit per year	1.05793
Mexico	2018	Gap 2 visit per year	269.464578
Micronesia (Federated States of)	2009	Gap 2 visit per year	-0.011056
Moldova	2019	Gap 2 visit per year	2.865988
Mongolia	2018	Gap 2 visit per year	4.894368
Montenegro	2018	Gap 2 visit per year	0.49534
Morocco	2017	Gap 2 visit per year	1.522672
Mozambique	2019	Gap 2 visit per year	-4.004304
Myanmar	2019	Gap 2 visit per year	1.299074

For peer review only - http://bmjopen.bmj.com/site/about/guidelines.xhtml

For peer review only

Namibia	2017	Gap 2 visit per year	0.043904
Nauru	2015	Gap 2 visit per year	0.004446
Nepal	2019	Gap 2 visit per year	2.982968
Netherlands	2018	Gap 2 visit per year	23.538066
New Zealand	2018	Gap 2 visit per year	6.286734
Nicaragua	2018	Gap 2 visit per year	3.709852
Niger	2016	Gap 2 visit per year	-3.291126
Nigeria	2018	Gap 2 visit per year	-0.780402
Niue	2008	Gap 2 visit per year	0.000912
North Korea	2017	Gap 2 visit per year	38.806026
Norway	2019	Gap 2 visit per year	10.853896
Oman	2019	Gap 2 visit per year	3.176352
Pakistan	2019	Gap 2 visit per year	61.240792
Palau	2014	Gap 2 visit per year	0.003602
Panama	2019	Gap 2 visit per year	2.092746
Papua New Guinea	2019	Gap 2 visit per year	-1.439412
Paraguay	2018	Gap 2 visit per year	1.573074
Peru	2018	Gap 2 visit per year	6.983458
Philippines	2017	Gap 2 visit per year	4.010112
Poland	2017	Gap 2 visit per year	19.535714
Portugal	2018	Gap 2 visit per year	21.69254
Qatar	2018	Gap 2 visit per year	2.516802
Romania	2017	Gap 2 visit per year	16.109986
Russian Federation	2018	Gap 2 visit per year	236.15102
Rwanda	2019	Gap 2 visit per year	-1.458938
Saint Kitts and Nevis	2018	Gap 2 visit per year	0.04331
Saint Lucia	2017	Gap 2 visit per year	-0.017676
Saint Vincent and the Grenadines	2012	Gap 2 visit per year	0.008464
Samoa	2016	Gap 2 visit per year	-0.019656
Sao Tome and Principe	2015	Gap 2 visit per year	-0.020052
Saudi Arabia	2018	Gap 2 visit per year	33.925084
Senegal	2019	Gap 2 visit per year	-2.639992
Serbia	2016	Gap 2 visit per year	8.132654
Seychelles	2018	Gap 2 visit per year	0.070284
Sierra Leone	2018	Gap 2 visit per year	-1.470258
Singapore	2016	Gap 2 visit per year	4.037452
Slovakia	2018	Gap 2 visit per year	6.328506
Slovenia	2018	Gap 2 visit per year	1.915262
Solomon Islands	2016	Gap 2 visit per year	-0.039796
Somalia	2014	Gap 2 visit per year	-2.89879
South Africa	2019	Gap 2 visit per year	2.10393
South Korea	2018	Gap 2 visit per year	40.329576
Spain	2018	Gap 2 visit per year	74.621782
Sri Lanka	2019	Gap 2 visit per year	3.57262
Sudan	2017	Gap 2 visit per year	-3.994594

For peer review only - http://bmjopen.bmj.com/site/about/guidelines.xhtml

Suriname	2018	Gap 2 visit per year	-0.00375
Swaziland	2016	Gap 2 visit per year	-0.226108
Sweden	2017	Gap 2 visit per year	16.89362
Switzerland	2018	Gap 2 visit per year	15.448128
Syrian Arab Republic	2016	Gap 2 visit per year	6.440694
Tajikistan	2014	Gap 2 visit per year	4.668892
Tanzania	2014	Gap 2 visit per year	-7.304342
Thailand	2019	Gap 2 visit per year	4.016032
The Gambia	2019	Gap 2 visit per year	-0.300898
Timor-Leste	2019	Gap 2 visit per year	0.206014
Togo	2019	Gap 2 visit per year	-1.369674
Tonga	2013	Gap 2 visit per year	-0.001054
Trinidad and Tobago	2019	Gap 2 visit per year	2.41459
Tunisia	2017	Gap 2 visit per year	3.360384
Turkey	2018	Gap 2 visit per year	46.295186
Turkmenistan	2014	Gap 2 visit per year	4.65549
Tuvalu	2014	Gap 2 visit per year	0.00058
Uganda	2017	Gap 2 visit per year	-2.052264
Ukraine	2014	Gap 2 visit per year	38.827668
United Arab Emirates	2018	Gap 2 visit per year	7.85723
United Kingdom	2019	Gap 2 visit per year	171.415648
United States of America	2018	Gap 2 visit per year	288.383206
Uruguay	2017	Gap 2 visit per year	6.802776
Uzbekistan	2014	Gap 2 visit per year	26.451046
Vanuatu	2016	Gap 2 visit per year	-0.045042
Venezuela	2017	Gap 2 visit per year	14.745746
Viet Nam	2016	Gap 2 visit per year	12.679208
Yemen	2014	Gap 2 visit per year	2.730936
Zambia	2016	Gap 2 visit per year	-1.78689
Zimbabwe	2018	Gap 2 visit per year	-1.952174
Afghanistan	2016	Gap 3 visit per year	-4.356644
Albania	2019	Gap 3 visit per year	0.197575
Algeria	2018	Gap 3 visit per year	16.98359
Andorra	2015	Gap 3 visit per year	0.078007
Angola	2017	Gap 3 visit per year	-4.659346
Antigua and Barbuda	2017	Gap 3 visit per year	0.070377
Argentina	2017	Gap 3 visit per year	56.085742
Armenia	2017	Gap 3 visit per year	3.982028
Australia	2018	Gap 3 visit per year	32.853299
Austria	2018	Gap 3 visit per year	16.408054
Azerbaijan	2014	Gap 3 visit per year	10.846171
Bahamas	2017	Gap 3 visit per year	0.113887
Bahrain	2015	Gap 3 visit per year	-0.15535
Bangladesh	2019	Gap 3 visit per year	-4.839636
Barbados	2017	Gap 3 visit per year	0.110246

For peer review only

For peer review only - http://bmjopen.bmj.com/site/about/guidelines.xhtml

Belarus	2015	Gap 3 visit per year	14.998519
Belgium	2019	Gap 3 visit per year	26.935488
Belize	2018	Gap 3 visit per year	0.041779
Benin	2019	Gap 3 visit per year	-2.447698
Bhutan	2019	Gap 3 visit per year	-0.227928
Bolivia	2017	Gap 3 visit per year	2.066446
Bosnia and Herzegovina	2015	Gap 3 visit per year	0.414561
Botswana	2018	Gap 3 visit per year	-0.827356
Brazil	2019	Gap 3 visit per year	89.905719
Brunei Darussalam	2017	Gap 3 visit per year	0.070822
Bulgaria	2018	Gap 3 visit per year	7.623696
Burkina Faso	2019	Gap 3 visit per year	-4.076975
Burundi	2017	Gap 3 visit per year	-2.210686
Cabo Verde	2015	Gap 3 visit per year	-0.055583
Cambodia	2014	Gap 3 visit per year	-2.383984
Cameroon	2011	Gap 3 visit per year	-4.962438
Canada	2019	Gap 3 visit per year	27.344624
Central African Republic	2015	Gap 3 visit per year	-1.443681
Chad	2019	Gap 3 visit per year	-3.435487
Chile	2018	Gap 3 visit per year	37.173216
China	2017	Gap 3 visit per year	650.807695
Colombia	2018	Gap 3 visit per year	73.833391
Comoros	2012	Gap 3 visit per year	-0.158988
Congo	2016	Gap 3 visit per year	-1.565321
Cook Islands	2014	Gap 3 visit per year	0.000131
Costa Rica	2018	Gap 3 visit per year	4.507123
Cote d'Ivoire	2019	Gap 3 visit per year	-6.200592
Croatia	2016	Gap 3 visit per year	1.799847
Cuba	2018	Gap 3 visit per year	38.562915
Cyprus	2016	Gap 3 visit per year	0.370479
Czech Republic	2019	Gap 3 visit per year	12.061618
DR Congo	2013	Gap 3 visit per year	-15.44584
Denmark	2018	Gap 3 visit per year	7.655486
Djibouti	2014	Gap 3 visit per year	-0.267162
Dominica	2018	Gap 3 visit per year	-0.013462
Dominican Republic	2019	Gap 3 visit per year	0.845385
Ecuador	2017	Gap 3 visit per year	13.062372
Egypt	2019	Gap 3 visit per year	-4.945897
El Salvador	2018	Gap 3 visit per year	6.633791
Equatorial Guinea	2017	Gap 3 visit per year	-0.077049
Eritrea	2004	Gap 3 visit per year	-0.837098
Estonia	2017	Gap 3 visit per year	1.186356
Ethiopia	2018	Gap 3 visit per year	-17.226724
Fiji	2015	Gap 3 visit per year	-0.054939
Finland	2018	Gap 3 visit per year	8.326295

For peer review only

France	2018	Gap 3 visit per year	169.821153
Gabon	2017	Gap 3 visit per year	0.092177
Georgia	2019	Gap 3 visit per year	11.023259
Germany	2018	Gap 3 visit per year	117.761587
Ghana	2019	Gap 3 visit per year	-8.461178
Greece	2018	Gap 3 visit per year	25.216552
Grenada	2018	Gap 3 visit per year	0.006176
Guatemala	2018	Gap 3 visit per year	-2.187623
Guinea	2016	Gap 3 visit per year	-3.122108
Guinea Bissau	2016	Gap 3 visit per year	-0.391307
Guyana	2018	Gap 3 visit per year	0.311702
Haiti	2018	Gap 3 visit per year	-4.078634
Honduras	2017	Gap 3 visit per year	-1.489143
Hungary	2018	Gap 3 visit per year	6.334698
Iceland	2019	Gap 3 visit per year	0.511437
India	2019	Gap 3 visit per year	77.358456
Indonesia	2019	Gap 3 visit per year	-86.49558
Iran	2018	Gap 3 visit per year	34.289903
Iraq	2018	Gap 3 visit per year	-5.297206
Ireland	2019	Gap 3 visit per year	5.183681
Israel	2019	Gap 3 visit per year	19.037294
Italy	2019	Gap 3 visit per year	192.965397
Jamaica	2016	Gap 3 visit per year	-1.117337
Japan	2018	Gap 3 visit per year	57.871913
Jordan	2017	Gap 3 visit per year	7.348699
Kazakhstan	2014	Gap 3 visit per year	24.206506
Kenya	2018	Gap 3 visit per year	-9.249434
Kiribati	2013	Gap 3 visit per year	-0.029863
Kuwait	2015	Gap 3 visit per year	3.079271
Kyrgyzstan	2014	Gap 3 visit per year	3.859661
Lao PDR	2017	Gap 3 visit per year	-0.726557
Latvia	2018	Gap 3 visit per year	1.340015
Lebanon	2018	Gap 3 visit per year	4.489076
Lesotho	2010	Gap 3 visit per year	-0.69138
Liberia	2015	Gap 3 visit per year	-1.385586
Libya	2017	Gap 3 visit per year	3.249205
Lithuania	2019	Gap 3 visit per year	4.101264
Luxembourg	2017	Gap 3 visit per year	0.515732
Macedonia (TFYR)	2015	Gap 3 visit per year	1.120465
Madagascar	2014	Gap 3 visit per year	-5.118054
Malawi	2018	Gap 3 visit per year	-3.39412
Malaysia	2015	Gap 3 visit per year	7.509111
Maldives	2018	Gap 3 visit per year	0.252775
Mali	2018	Gap 3 visit per year	-3.600798
Malta	2015	Gap 3 visit per year	0.314099

For peer review only

For peer review only - http://bmjopen.bmj.com/site/about/guidelines.xhtml

Marshall Islands	2012	Gap 3 visit per year	-0.005922
Mauritania	2018	Gap 3 visit per year	-0.829601
Mauritius	2018	Gap 3 visit per year	0.784395
Mexico	2018	Gap 3 visit per year	251.204367
Micronesia (Federated States of)	2009	Gap 3 visit per year	-0.021584
Moldova	2019	Gap 3 visit per year	1.712732
Mongolia	2018	Gap 3 visit per year	4.288802
Montenegro	2018	Gap 3 visit per year	0.31051
Morocco	2017	Gap 3 visit per year	-4.216742
Mozambique	2019	Gap 3 visit per year	-6.648956
Myanmar	2019	Gap 3 visit per year	-8.007889
Namibia	2017	Gap 3 visit per year	-0.289394
Nauru	2015	Gap 3 visit per year	0.003169
Nepal	2019	Gap 3 visit per year	-1.312048
Netherlands	2018	Gap 3 visit per year	19.498849
New Zealand	2018	Gap 3 visit per year	5.374101
Nicaragua	2018	Gap 3 visit per year	2.874278
Niger	2016	Gap 3 visit per year	-5.161689
Nigeria	2018	Gap 3 visit per year	-19.806353
Niue	2008	Gap 3 visit per year	0.000618
North Korea	2017	Gap 3 visit per year	34.792289
Norway	2019	Gap 3 visit per year	9.711844
Oman	2019	Gap 3 visit per year	2.364028
Pakistan	2019	Gap 3 visit per year	31.336438
Palau	2014	Gap 3 visit per year	-0.000847
Panama	2019	Gap 3 visit per year	1.406119
Papua New Guinea	2019	Gap 3 visit per year	-2.304118
Paraguay	2018	Gap 3 visit per year	0.004361
Peru	2018	Gap 3 visit per year	3.917437
Philippines	2017	Gap 3 visit per year	-9.770082
Poland	2017	Gap 3 visit per year	6.732571
Portugal	2018	Gap 3 visit per year	18.91931
Qatar	2018	Gap 3 visit per year	2.046953
Romania	2017	Gap 3 visit per year	9.519229
Russian Federation	2018	Gap 3 visit per year	192.47828
Rwanda	2019	Gap 3 visit per year	-2.561407
Saint Kitts and Nevis	2018	Gap 3 visit per year	0.028715
Saint Lucia	2017	Gap 3 visit per year	-0.055514
Saint Vincent and the Grenadines	2012	Gap 3 visit per year	-0.012804
Samoa	2016	Gap 3 visit per year	-0.046234
Sao Tome and Principe	2015	Gap 3 visit per year	-0.045828
Saudi Arabia	2018	Gap 3 visit per year	28.881876
Senegal	2019	Gap 3 visit per year	-4.318738
Serbia	2016	Gap 3 visit per year	5.308231
Seychelles	2018	Gap 3 visit per year	0.045426

For peer review only

Sierra Leone	2018	Gap 3 visit per year	-2.346887
Singapore	2016	Gap 3 visit per year	2.814428
Slovakia	2018	Gap 3 visit per year	4.698259
Slovenia	2018	Gap 3 visit per year	1.225143
Solomon Islands	2016	Gap 3 visit per year	-0.089694
Somalia	2014	Gap 3 visit per year	-4.425435
South Africa	2019	Gap 3 visit per year	-8.442355
South Korea	2018	Gap 3 visit per year	29.686864
Spain	2018	Gap 3 visit per year	64.891173
Sri Lanka	2019	Gap 3 visit per year	-0.79232
Sudan	2017	Gap 3 visit per year	-8.662641
Suriname	2018	Gap 3 visit per year	-0.123625
Swaziland	2016	Gap 3 visit per year	-0.365912
Sweden	2017	Gap 3 visit per year	14.61593
Switzerland	2018	Gap 3 visit per year	13.937192
Syrian Arab Republic	2016	Gap 3 visit per year	4.039791
Tajikistan	2014	Gap 3 visit per year	3.448588
Tanzania	2014	Gap 3 visit per year	-11.710263
Thailand	2019	Gap 3 visit per year	-9.969452
The Gambia	2019	Gap 3 visit per year	-0.514347
Timor-Leste	2019	Gap 3 visit per year	0.060521
Togo	2019	Gap 3 visit per year	-2.211261
Tonga	2013	Gap 3 visit per year	-0.015331
Trinidad and Tobago	2019	Gap 3 visit per year	2.060885
Tunisia	2017	Gap 3 visit per year	1.317576
Turkey	2018	Gap 3 visit per year	32.255029
Turkmenistan	2014	Gap 3 visit per year	3.942985
Tuvalu	2014	Gap 3 visit per year	-0.00163
Uganda	2017	Gap 3 visit per year	-4.807896
Ukraine	2014	Gap 3 visit per year	24.495002
United Arab Emirates	2018	Gap 3 visit per year	5.699595
United Kingdom	2019	Gap 3 visit per year	158.811722
United States of America	2018	Gap 3 visit per year	219.664559
Uruguay	2017	Gap 3 visit per year	5.960164
Uzbekistan	2014	Gap 3 visit per year	21.617319
Vanuatu	2016	Gap 3 visit per year	-0.079063
Venezuela	2017	Gap 3 visit per year	9.402119
Viet Nam	2016	Gap 3 visit per year	-0.365938
Yemen	2014	Gap 3 visit per year	0.706404
Zambia	2016	Gap 3 visit per year	-3.058835
Zimbabwe	2018	Gap 3 visit per year	-3.684761
Afghanistan	2016	Gap 4 visit per year	-7.449192
Albania	2019	Gap 4 visit per year	-0.5274
Algeria	2018	Gap 4 visit per year	10.54412
Andorra	2015	Gap 4 visit per year	0.060676

For peer review only

Angola	2017	Gap 4 visit per year	-7.279128
Antigua and Barbuda	2017	Gap 4 visit per year	0.049836
Argentina	2017	Gap 4 visit per year	45.562156
Armenia	2017	Gap 4 visit per year	3.148704
Australia	2018	Gap 4 visit per year	28.203732
Austria	2018	Gap 4 visit per year	14.154572
Azerbaijan	2014	Gap 4 visit per year	9.002228
Bahamas	2017	Gap 4 visit per year	0.028516
Bahrain	2015	Gap 4 visit per year	-0.4188
Bangladesh	2019	Gap 4 visit per year	-23.754348
Barbados	2017	Gap 4 visit per year	0.028328
Belarus	2015	Gap 4 visit per year	11.832192
Belgium	2019	Gap 4 visit per year	24.459984
Belize	2018	Gap 4 visit per year	-0.013128
Benin	2019	Gap 4 visit per year	-3.390764
Bhutan	2019	Gap 4 visit per year	-0.362404
Bolivia	2017	Gap 4 visit per year	0.833928
Bosnia and Herzegovina	2015	Gap 4 visit per year	-0.682752
Botswana	2018	Gap 4 visit per year	-1.211808
Brazil	2019	Gap 4 visit per year	38.597792
Brunei Darussalam	2017	Gap 4 visit per year	-0.019404
Bulgaria	2018	Gap 4 visit per year	5.220428
Burkina Faso	2019	Gap 4 visit per year	-5.7543
Burundi	2017	Gap 4 visit per year	-3.128248
Cabo Verde	2015	Gap 4 visit per year	-0.142444
Cambodia	2014	Gap 4 visit per year	-3.669312
Cameroon	2011	Gap 4 visit per year	-6.923584
Canada	2019	Gap 4 visit per year	21.230332
Central African Republic	2015	Gap 4 visit per year	-1.978908
Chad	2019	Gap 4 visit per year	-4.724816
Chile	2018	Gap 4 visit per year	33.387288
China	2017	Gap 4 visit per year	396.24376
Colombia	2018	Gap 4 visit per year	66.627188
Comoros	2012	Gap 4 visit per year	-0.232484
Congo	2016	Gap 4 visit per year	-2.175428
Cook Islands	2014	Gap 4 visit per year	-0.003992
Costa Rica	2018	Gap 4 visit per year	3.598164
Cote d'Ivoire	2019	Gap 4 visit per year	-8.962956
Croatia	2016	Gap 4 visit per year	0.295796
Cuba	2018	Gap 4 visit per year	35.50622
Cyprus	2016	Gap 4 visit per year	0.113472
Czech Republic	2019	Gap 4 visit per year	8.749824
DR Congo	2013	Gap 4 visit per year	-21.66412
Denmark	2018	Gap 4 visit per year	6.157148
Djibouti	2014	Gap 4 visit per year	-0.389716

For peer review only

Dominica	2018	Gap 4 visit per year	-0.031116
Dominican Republic	2019	Gap 4 visit per year	-1.47132
Ecuador	2017	Gap 4 visit per year	11.205996
Egypt	2019	Gap 4 visit per year	-19.081696
El Salvador	2018	Gap 4 visit per year	5.773888
Equatorial Guinea	2017	Gap 4 visit per year	-0.187232
Eritrea	2004	Gap 4 visit per year	-1.151964
Estonia	2017	Gap 4 visit per year	0.820308
Ethiopia	2018	Gap 4 visit per year	-24.368132
Fiji	2015	Gap 4 visit per year	-0.197752
Finland	2018	Gap 4 visit per year	6.83056
France	2018	Gap 4 visit per year	155.649204
Gabon	2017	Gap 4 visit per year	-0.111764
Georgia	2019	Gap 4 visit per year	9.982512
Germany	2018	Gap 4 visit per year	97.448616
Ghana	2019	Gap 4 visit per year	-11.820904
Greece	2018	Gap 4 visit per year	22.703236
Grenada	2018	Gap 4 visit per year	-0.018432
Guatemala	2018	Gap 4 visit per year	-3.937164
Guinea	2016	Gap 4 visit per year	-4.325644
Guinea Bissau	2016	Gap 4 visit per year	-0.559576
Guyana	2018	Gap 4 visit per year	0.179436
Haiti	2018	Gap 4 visit per year	-5.872512
Honduras	2017	Gap 4 visit per year	-2.471024
Hungary	2018	Gap 4 visit per year	2.933264
Iceland	2019	Gap 4 visit per year	0.447916
India	2019	Gap 4 visit per year	-108.217392
Indonesia	2019	Gap 4 visit per year	-136.30444
Iran	2018	Gap 4 visit per year	24.119204
Iraq	2018	Gap 4 visit per year	-11.597608
Ireland	2019	Gap 4 visit per year	4.183908
Israel	2019	Gap 4 visit per year	17.620892
Italy	2019	Gap 4 visit per year	176.422196
Jamaica	2016	Gap 4 visit per year	-1.710116
Japan	2018	Gap 4 visit per year	24.594884
Jordan	2017	Gap 4 visit per year	6.008432
Kazakhstan	2014	Gap 4 visit per year	20.798008
Kenya	2018	Gap 4 visit per year	-13.672912
Kiribati	2013	Gap 4 visit per year	-0.043484
Kuwait	2015	Gap 4 visit per year	2.414028
Kyrgyzstan	2014	Gap 4 visit per year	2.990548
Lao PDR	2017	Gap 4 visit per year	-1.400576
Latvia	2018	Gap 4 visit per year	0.72552
Lebanon	2018	Gap 4 visit per year	3.580268
Lesotho	2010	Gap 4 visit per year	-0.94484

For peer review only - http://bmjopen.bmj.com/site/about/guidelines.xhtml

For peer review only

Liberia	2015	Gap 4 visit per year	-1.875448
Libya	2017	Gap 4 visit per year	2.03944
Lithuania	2019	Gap 4 visit per year	3.150352
Luxembourg	2017	Gap 4 visit per year	0.390976
Macedonia (TFYR)	2015	Gap 4 visit per year	0.49812
Madagascar	2014	Gap 4 visit per year	-7.536572
Malawi	2018	Gap 4 visit per year	-4.63366
Malaysia	2015	Gap 4 visit per year	2.263648
Maldives	2018	Gap 4 visit per year	0.1902
Mali	2018	Gap 4 visit per year	-5.210064
Malta	2015	Gap 4 visit per year	0.212132
Marshall Islands	2012	Gap 4 visit per year	-0.011896
Mauritania	2018	Gap 4 visit per year	-1.242968
Mauritius	2018	Gap 4 visit per year	0.51086
Mexico	2018	Gap 4 visit per year	232.944156
Micronesia (Federated States of)	2009	Gap 4 visit per year	-0.032112
Moldova	2019	Gap 4 visit per year	0.559476
Mongolia	2018	Gap 4 visit per year	3.683236
Montenegro	2018	Gap 4 visit per year	0.12568
Morocco	2017	Gap 4 visit per year	-9.956156
Mozambique	2019	Gap 4 visit per year	-9.293608
Myanmar	2019	Gap 4 visit per year	-17.314852
Namibia	2017	Gap 4 visit per year	-0.622692
Nauru	2015	Gap 4 visit per year	0.001892
Nepal	2019	Gap 4 visit per year	-5.607064
Netherlands	2018	Gap 4 visit per year	15.459632
New Zealand	2018	Gap 4 visit per year	4.461468
Nicaragua	2018	Gap 4 visit per year	2.038704
Niger	2016	Gap 4 visit per year	-7.032252
Nigeria	2018	Gap 4 visit per year	-38.832304
Niue	2008	Gap 4 visit per year	0.000324
North Korea	2017	Gap 4 visit per year	30.778552
Norway	2019	Gap 4 visit per year	8.569792
Oman	2019	Gap 4 visit per year	1.551704
Pakistan	2019	Gap 4 visit per year	1.432084
Palau	2014	Gap 4 visit per year	-0.005296
Panama	2019	Gap 4 visit per year	0.719492
Papua New Guinea	2019	Gap 4 visit per year	-3.168824
Paraguay	2018	Gap 4 visit per year	-1.564352
Peru	2018	Gap 4 visit per year	0.851416
Philippines	2017	Gap 4 visit per year	-23.550276
Poland	2017	Gap 4 visit per year	-6.070572
Portugal	2018	Gap 4 visit per year	16.14608
Qatar	2018	Gap 4 visit per year	1.577104
Romania	2017	Gap 4 visit per year	2.928472

For peer review only

Russian Federation	2018	Gap 4 visit per year	148.80554
Rwanda	2019	Gap 4 visit per year	-3.663876
Saint Kitts and Nevis	2018	Gap 4 visit per year	0.01412
Saint Lucia	2017	Gap 4 visit per year	-0.093352
Saint Vincent and the Grenadines	2012	Gap 4 visit per year	-0.034072
Samoa	2016	Gap 4 visit per year	-0.072812
Sao Tome and Principe	2015	Gap 4 visit per year	-0.071604
Saudi Arabia	2018	Gap 4 visit per year	23.838668
Senegal	2019	Gap 4 visit per year	-5.997484
Serbia	2016	Gap 4 visit per year	2.483808
Seychelles	2018	Gap 4 visit per year	0.020568
Sierra Leone	2018	Gap 4 visit per year	-3.223516
Singapore	2016	Gap 4 visit per year	1.591404
Slovakia	2018	Gap 4 visit per year	3.068012
Slovenia	2018	Gap 4 visit per year	0.535024
Solomon Islands	2016	Gap 4 visit per year	-0.139592
Somalia	2014	Gap 4 visit per year	-5.95208
South Africa	2019	Gap 4 visit per year	-18.98864
South Korea	2018	Gap 4 visit per year	19.044152
Spain	2018	Gap 4 visit per year	55.160564
Sri Lanka	2019	Gap 4 visit per year	-5.15726
Sudan	2017	Gap 4 visit per year	-13.330688
Suriname	2018	Gap 4 visit per year	-0.2435
Swaziland	2016	Gap 4 visit per year	-0.505716
Sweden	2017	Gap 4 visit per year	12.33824
Switzerland	2018	Gap 4 visit per year	12.426256
Syrian Arab Republic	2016	Gap 4 visit per year	1.638888
Tajikistan	2014	Gap 4 visit per year	2.228284
Tanzania	2014	Gap 4 visit per year	-16.116184
Thailand	2019	Gap 4 visit per year	-23.954936
The Gambia	2019	Gap 4 visit per year	-0.727796
Timor-Leste	2019	Gap 4 visit per year	-0.084972
Togo	2019	Gap 4 visit per year	-3.052848
Tonga	2013	Gap 4 visit per year	-0.029608
Trinidad and Tobago	2019	Gap 4 visit per year	1.70718
Tunisia	2017	Gap 4 visit per year	-0.725232
Turkey	2018	Gap 4 visit per year	18.214872
Turkmenistan	2014	Gap 4 visit per year	3.23048
Tuvalu	2014	Gap 4 visit per year	-0.00384
Uganda	2017	Gap 4 visit per year	-7.563528
Ukraine	2014	Gap 4 visit per year	10.162336
United Arab Emirates	2018	Gap 4 visit per year	3.54196
United Kingdom	2019	Gap 4 visit per year	146.207796
United States of America	2018	Gap 4 visit per year	150.945912
Uruguay	2017	Gap 4 visit per year	5.117552

For peer review only

For peer review only - http://bmjopen.bmj.com/site/about/guidelines.xhtml

Uzbekistan	2014	Gap 4 visit per year	16.783592
Vanuatu	2016	Gap 4 visit per year	-0.113084
Venezuela	2017	Gap 4 visit per year	4.058492
Viet Nam	2016	Gap 4 visit per year	-13.411084
Yemen	2014	Gap 4 visit per year	-1.318128
Zambia	2016	Gap 4 visit per year	-4.33078
Zimbabwe	2018	Gap 4 visit per year	-5.417348
Afghanistan	2016	Gap 5 visit per year	-10.54174
Albania	2019	Gap 5 visit per year	-1.252375
Algeria	2018	Gap 5 visit per year	4.10465
Andorra	2015	Gap 5 visit per year	0.043345
Angola	2017	Gap 5 visit per year	-9.89891
Antigua and Barbuda	2017	Gap 5 visit per year	0.029295
Argentina	2017	Gap 5 visit per year	35.03857
Armenia	2017	Gap 5 visit per year	2.31538
Australia	2018	Gap 5 visit per year	23.554165
Austria	2018	Gap 5 visit per year	11.90109
Azerbaijan	2014	Gap 5 visit per year	7.158285
Bahamas	2017	Gap 5 visit per year	-0.056855
Bahrain	2015	Gap 5 visit per year	-0.68225
Bangladesh	2019	Gap 5 visit per year	-42.66906
Barbados	2017	Gap 5 visit per year	-0.05359
Belarus	2015	Gap 5 visit per year	8.665865
Belgium	2019	Gap 5 visit per year	21.98448
Belize	2018	Gap 5 visit per year	-0.068035
Benin	2019	Gap 5 visit per year	-4.33383
Bhutan	2019	Gap 5 visit per year	-0.49688
Bolivia	2017	Gap 5 visit per year	-0.39859
Bosnia and Herzegovina	2015	Gap 5 visit per year	-1.780065
Botswana	2018	Gap 5 visit per year	-1.59626
Brazil	2019	Gap 5 visit per year	-12.710135
Brunei Darussalam	2017	Gap 5 visit per year	-0.10963
Bulgaria	2018	Gap 5 visit per year	2.81716
Burkina Faso	2019	Gap 5 visit per year	-7.431625
Burundi	2017	Gap 5 visit per year	-4.04581
Cabo Verde	2015	Gap 5 visit per year	-0.229305
Cambodia	2014	Gap 5 visit per year	-4.95464
Cameroon	2011	Gap 5 visit per year	-8.88473
Canada	2019	Gap 5 visit per year	15.11604
Central African Republic	2015	Gap 5 visit per year	-2.514135
Chad	2019	Gap 5 visit per year	-6.014145
Chile	2018	Gap 5 visit per year	29.60136
China	2017	Gap 5 visit per year	141.679825
Colombia	2018	Gap 5 visit per year	59.420985
Comoros	2012	Gap 5 visit per year	-0.30598

For peer review only - http://bmjopen.bmj.com/site/about/guidelines.xhtml

Congo	2016	Gap 5 visit per year	-2.785535
Cook Islands	2014	Gap 5 visit per year	-0.008115
Costa Rica	2018	Gap 5 visit per year	2.689205
Cote d'Ivoire	2019	Gap 5 visit per year	-11.72532
Croatia	2016	Gap 5 visit per year	-1.208255
Cuba	2018	Gap 5 visit per year	32.449525
Cyprus	2016	Gap 5 visit per year	-0.143535
Czech Republic	2019	Gap 5 visit per year	5.43803
DR Congo	2013	Gap 5 visit per year	-27.8824
Denmark	2018	Gap 5 visit per year	4.65881
Djibouti	2014	Gap 5 visit per year	-0.51227
Dominica	2018	Gap 5 visit per year	-0.04877
Dominican Republic	2019	Gap 5 visit per year	-3.788025
Ecuador	2017	Gap 5 visit per year	9.34962
Egypt	2019	Gap 5 visit per year	-33.217495
El Salvador	2018	Gap 5 visit per year	4.913985
Equatorial Guinea	2017	Gap 5 visit per year	-0.297415
Eritrea	2004	Gap 5 visit per year	-1.46683
Estonia	2017	Gap 5 visit per year	0.45426
Ethiopia	2018	Gap 5 visit per year	-31.50954
Fiji	2015	Gap 5 visit per year	-0.340565
Finland	2018	Gap 5 visit per year	5.334825
France	2018	Gap 5 visit per year	141.477255
Gabon	2017	Gap 5 visit per year	-0.315705
Georgia	2019	Gap 5 visit per year	8.941765
Germany	2018	Gap 5 visit per year	77.135645
Ghana	2019	Gap 5 visit per year	-15.18063
Greece	2018	Gap 5 visit per year	20.18992
Grenada	2018	Gap 5 visit per year	-0.04304
Guatemala	2018	Gap 5 visit per year	-5.686705
Guinea	2016	Gap 5 visit per year	-5.52918
Guinea Bissau	2016	Gap 5 visit per year	-0.727845
Guyana	2018	Gap 5 visit per year	0.04717
Haiti	2018	Gap 5 visit per year	-7.66639
Honduras	2017	Gap 5 visit per year	-3.452905
Hungary	2018	Gap 5 visit per year	-0.46817
Iceland	2019	Gap 5 visit per year	0.384395
India	2019	Gap 5 visit per year	-293.79324
Indonesia	2019	Gap 5 visit per year	-186.1133
Iran	2018	Gap 5 visit per year	13.948505
Iraq	2018	Gap 5 visit per year	-17.89801
Ireland	2019	Gap 5 visit per year	3.184135
Israel	2019	Gap 5 visit per year	16.20449
Italy	2019	Gap 5 visit per year	159.878995
Jamaica	2016	Gap 5 visit per year	-2.302895

For peer review only - http://bmjopen.bmj.com/site/about/guidelines.xhtml

Japan	2018	Gap 5 visit per year	-8.682145
Jordan	2017	Gap 5 visit per year	4.668165
Kazakhstan	2014	Gap 5 visit per year	17.38951
Kenya	2018	Gap 5 visit per year	-18.09639
Kiribati	2013	Gap 5 visit per year	-0.057105
Kuwait	2015	Gap 5 visit per year	1.748785
Kyrgyzstan	2014	Gap 5 visit per year	2.121435
Lao PDR	2017	Gap 5 visit per year	-2.074595
Latvia	2018	Gap 5 visit per year	0.111025
Lebanon	2018	Gap 5 visit per year	2.67146
Lesotho	2010	Gap 5 visit per year	-1.1983
Liberia	2015	Gap 5 visit per year	-2.36531
Libya	2017	Gap 5 visit per year	0.829675
Lithuania	2019	Gap 5 visit per year	2.19944
Luxembourg	2017	Gap 5 visit per year	0.26622
Macedonia (TFYR)	2015	Gap 5 visit per year	-0.124225
Madagascar	2014	Gap 5 visit per year	-9.95509
Malawi	2018	Gap 5 visit per year	-5.8732
Malaysia	2015	Gap 5 visit per year	-2.981815
Maldives	2018	Gap 5 visit per year	0.127625
Mali	2018	Gap 5 visit per year	-6.81933
Malta	2015	Gap 5 visit per year	0.110165
Marshall Islands	2012	Gap 5 visit per year	-0.01787
Mauritania	2018	Gap 5 visit per year	-1.656335
Mauritius	2018	Gap 5 visit per year	0.237325
Mexico	2018	Gap 5 visit per year	214.683945
Micronesia (Federated States of)	2009	Gap 5 visit per year	-0.04264
Moldova	2019	Gap 5 visit per year	-0.59378
Mongolia	2018	Gap 5 visit per year	3.07767
Montenegro	2018	Gap 5 visit per year	-0.05915
Morocco	2017	Gap 5 visit per year	-15.69557
Mozambique	2019	Gap 5 visit per year	-11.93826
Myanmar	2019	Gap 5 visit per year	-26.621815
Namibia	2017	Gap 5 visit per year	-0.95599
Nauru	2015	Gap 5 visit per year	0.000615
Nepal	2019	Gap 5 visit per year	-9.90208
Netherlands	2018	Gap 5 visit per year	11.420415
New Zealand	2018	Gap 5 visit per year	3.548835
Nicaragua	2018	Gap 5 visit per year	1.20313
Niger	2016	Gap 5 visit per year	-8.902815
Nigeria	2018	Gap 5 visit per year	-57.858255
Niue	2008	Gap 5 visit per year	3.00E-05
North Korea	2017	Gap 5 visit per year	26.764815
Norway	2019	Gap 5 visit per year	7.42774
Oman	2019	Gap 5 visit per year	0.73938

For peer review only

Pakistan	2019	Gap 5 visit per year	-28.47227
Palau	2014	Gap 5 visit per year	-0.009745
Panama	2019	Gap 5 visit per year	0.032865
Papua New Guinea	2019	Gap 5 visit per year	-4.03353
Paraguay	2018	Gap 5 visit per year	-3.133065
Peru	2018	Gap 5 visit per year	-2.214605
Philippines	2017	Gap 5 visit per year	-37.33047
Poland	2017	Gap 5 visit per year	-18.873715
Portugal	2018	Gap 5 visit per year	13.37285
Qatar	2018	Gap 5 visit per year	1.107255
Romania	2017	Gap 5 visit per year	-3.662285
Russian Federation	2018	Gap 5 visit per year	105.1328
Rwanda	2019	Gap 5 visit per year	-4.766345
Saint Kitts and Nevis	2018	Gap 5 visit per year	-0.000475
Saint Lucia	2017	Gap 5 visit per year	-0.13119
Saint Vincent and the Grenadines	2012	Gap 5 visit per year	-0.05534
Samoa	2016	Gap 5 visit per year	-0.09939
Sao Tome and Principe	2015	Gap 5 visit per year	-0.09738
Saudi Arabia	2018	Gap 5 visit per year	18.79546
Senegal	2019	Gap 5 visit per year	-7.67623
Serbia	2016	Gap 5 visit per year	-0.340615
Seychelles	2018	Gap 5 visit per year	-0.00429
Sierra Leone	2018	Gap 5 visit per year	-4.100145
Singapore	2016	Gap 5 visit per year	0.36838
Slovakia	2018	Gap 5 visit per year	1.437765
Slovenia	2018	Gap 5 visit per year	-0.155095
Solomon Islands	2016	Gap 5 visit per year	-0.18949
Somalia	2014	Gap 5 visit per year	-7.478725
South Africa	2019	Gap 5 visit per year	-29.534925
South Korea	2018	Gap 5 visit per year	8.40144
Spain	2018	Gap 5 visit per year	45.429955
Sri Lanka	2019	Gap 5 visit per year	-9.5222
Sudan	2017	Gap 5 visit per year	-17.998735
Suriname	2018	Gap 5 visit per year	-0.363375
Swaziland	2016	Gap 5 visit per year	-0.64552
Sweden	2017	Gap 5 visit per year	10.06055
Switzerland	2018	Gap 5 visit per year	10.91532
Syrian Arab Republic	2016	Gap 5 visit per year	-0.762015
Tajikistan	2014	Gap 5 visit per year	1.00798
Tanzania	2014	Gap 5 visit per year	-20.522105
Thailand	2019	Gap 5 visit per year	-37.94042
The Gambia	2019	Gap 5 visit per year	-0.941245
Timor-Leste	2019	Gap 5 visit per year	-0.230465
Togo	2019	Gap 5 visit per year	-3.894435
Tonga	2013	Gap 5 visit per year	-0.043885

For peer review only

Trinidad and Tobago	2019	Gap 5 visit per year	1.353475
Tunisia	2017	Gap 5 visit per year	-2.76804
Turkey	2018	Gap 5 visit per year	4.174715
Turkmenistan	2014	Gap 5 visit per year	2.517975
Tuvalu	2014	Gap 5 visit per year	-0.00605
Uganda	2017	Gap 5 visit per year	-10.31916
Ukraine	2014	Gap 5 visit per year	-4.17033
United Arab Emirates	2018	Gap 5 visit per year	1.384325
United Kingdom	2019	Gap 5 visit per year	133.60387
United States of America	2018	Gap 5 visit per year	82.227265
Uruguay	2017	Gap 5 visit per year	4.27494
Uzbekistan	2014	Gap 5 visit per year	11.949865
Vanuatu	2016	Gap 5 visit per year	-0.147105
Venezuela	2017	Gap 5 visit per year	-1.285135
Viet Nam	2016	Gap 5 visit per year	-26.45623
Yemen	2014	Gap 5 visit per year	-3.34266
Zambia	2016	Gap 5 visit per year	-5.602725
Zimbabwe	2018	Gap 5 visit per year	-7.149935
Afghanistan	2016	Gap 6 visit per year	-13.634288
Albania	2019	Gap 6 visit per year	-1.97735
Algeria	2018	Gap 6 visit per year	-2.33482
Andorra	2015	Gap 6 visit per year	0.026014
Angola	2017	Gap 6 visit per year	-12.518692
Antigua and Barbuda	2017	Gap 6 visit per year	0.008754
Argentina	2017	Gap 6 visit per year	24.514984
Armenia	2017	Gap 6 visit per year	1.482056
Australia	2018	Gap 6 visit per year	18.904598
Austria	2018	Gap 6 visit per year	9.647608
Azerbaijan	2014	Gap 6 visit per year	5.314342
Bahamas	2017	Gap 6 visit per year	-0.142226
Bahrain	2015	Gap 6 visit per year	-0.9457
Bangladesh	2019	Gap 6 visit per year	-61.583772
Barbados	2017	Gap 6 visit per year	-0.135508
Belarus	2015	Gap 6 visit per year	5.499538
Belgium	2019	Gap 6 visit per year	19.508976
Belize	2018	Gap 6 visit per year	-0.122942
Benin	2019	Gap 6 visit per year	-5.276896
Bhutan	2019	Gap 6 visit per year	-0.631356
Bolivia	2017	Gap 6 visit per year	-1.631108
Bosnia and Herzegovina	2015	Gap 6 visit per year	-2.877378
Botswana	2018	Gap 6 visit per year	-1.980712
Brazil	2019	Gap 6 visit per year	-64.018062
Brunei Darussalam	2017	Gap 6 visit per year	-0.199856
Bulgaria	2018	Gap 6 visit per year	0.413892
Burkina Faso	2019	Gap 6 visit per year	-9.10895

For peer review only

Burundi	2017	Gap 6 visit per year	-4.963372
Cabo Verde	2015	Gap 6 visit per year	-0.316166
Cambodia	2014	Gap 6 visit per year	-6.239968
Cameroon	2011	Gap 6 visit per year	-10.845876
Canada	2019	Gap 6 visit per year	9.001748
Central African Republic	2015	Gap 6 visit per year	-3.049362
Chad	2019	Gap 6 visit per year	-7.303474
Chile	2018	Gap 6 visit per year	25.815432
China	2017	Gap 6 visit per year	-112.88411
Colombia	2018	Gap 6 visit per year	52.214782
Comoros	2012	Gap 6 visit per year	-0.379476
Congo	2016	Gap 6 visit per year	-3.395642
Cook Islands	2014	Gap 6 visit per year	-0.012238
Costa Rica	2018	Gap 6 visit per year	1.780246
Cote d'Ivoire	2019	Gap 6 visit per year	-14.487684
Croatia	2016	Gap 6 visit per year	-2.712306
Cuba	2018	Gap 6 visit per year	29.39283
Cyprus	2016	Gap 6 visit per year	-0.400542
Czech Republic	2019	Gap 6 visit per year	2.126236
DR Congo	2013	Gap 6 visit per year	-34.10068
Denmark	2018	Gap 6 visit per year	3.160472
Djibouti	2014	Gap 6 visit per year	-0.634824
Dominica	2018	Gap 6 visit per year	-0.066424
Dominican Republic	2019	Gap 6 visit per year	-6.10473
Ecuador	2017	Gap 6 visit per year	7.493244
Egypt	2019	Gap 6 visit per year	-47.353294
El Salvador	2018	Gap 6 visit per year	4.054082
Equatorial Guinea	2017	Gap 6 visit per year	-0.407598
Eritrea	2004	Gap 6 visit per year	-1.781696
Estonia	2017	Gap 6 visit per year	0.088212
Ethiopia	2018	Gap 6 visit per year	-38.650948
Fiji	2015	Gap 6 visit per year	-0.483378
Finland	2018	Gap 6 visit per year	3.83909
France	2018	Gap 6 visit per year	127.305306
Gabon	2017	Gap 6 visit per year	-0.519646
Georgia	2019	Gap 6 visit per year	7.901018
Germany	2018	Gap 6 visit per year	56.822674
Ghana	2019	Gap 6 visit per year	-18.540356
Greece	2018	Gap 6 visit per year	17.676604
Grenada	2018	Gap 6 visit per year	-0.067648
Guatemala	2018	Gap 6 visit per year	-7.436246
Guinea	2016	Gap 6 visit per year	-6.732716
Guinea Bissau	2016	Gap 6 visit per year	-0.896114
Guyana	2018	Gap 6 visit per year	-0.085096
Haiti	2018	Gap 6 visit per year	-9.460268

For peer review only

For peer review only - http://bmjopen.bmj.com/site/about/guidelines.xhtml

Honduras	2017	Gap 6 visit per year	-4.434786
Hungary	2018	Gap 6 visit per year	-3.869604
Iceland	2019	Gap 6 visit per year	0.320874
India	2019	Gap 6 visit per year	-479.369088
Indonesia	2019	Gap 6 visit per year	-235.92216
Iran	2018	Gap 6 visit per year	3.777806
Iraq	2018	Gap 6 visit per year	-24.198412
Ireland	2019	Gap 6 visit per year	2.184362
Israel	2019	Gap 6 visit per year	14.788088
Italy	2019	Gap 6 visit per year	143.335794
Jamaica	2016	Gap 6 visit per year	-2.895674
Japan	2018	Gap 6 visit per year	-41.959174
Jordan	2017	Gap 6 visit per year	3.327898
Kazakhstan	2014	Gap 6 visit per year	13.981012
Kenya	2018	Gap 6 visit per year	-22.519868
Kiribati	2013	Gap 6 visit per year	-0.070726
Kuwait	2015	Gap 6 visit per year	1.083542
Kyrgyzstan	2014	Gap 6 visit per year	1.252322
Lao PDR	2017	Gap 6 visit per year	-2.748614
Latvia	2018	Gap 6 visit per year	-0.50347
Lebanon	2018	Gap 6 visit per year	1.762652
Lesotho	2010	Gap 6 visit per year	-1.45176
Liberia	2015	Gap 6 visit per year	-2.855172
Libya	2017	Gap 6 visit per year	-0.38009
Lithuania	2019	Gap 6 visit per year	1.248528
Luxembourg	2017	Gap 6 visit per year	0.141464
Macedonia (TFYR)	2015	Gap 6 visit per year	-0.74657
Madagascar	2014	Gap 6 visit per year	-12.373608
Malawi	2018	Gap 6 visit per year	-7.11274
Malaysia	2015	Gap 6 visit per year	-8.227278
Maldives	2018	Gap 6 visit per year	0.06505
Mali	2018	Gap 6 visit per year	-8.428596
Malta	2015	Gap 6 visit per year	0.008198
Marshall Islands	2012	Gap 6 visit per year	-0.023844
Mauritania	2018	Gap 6 visit per year	-2.069702
Mauritius	2018	Gap 6 visit per year	-0.03621
Mexico	2018	Gap 6 visit per year	196.423734
Micronesia (Federated States of)	2009	Gap 6 visit per year	-0.053168
Moldova	2019	Gap 6 visit per year	-1.747036
Mongolia	2018	Gap 6 visit per year	2.472104
Montenegro	2018	Gap 6 visit per year	-0.24398
Morocco	2017	Gap 6 visit per year	-21.434984
Mozambique	2019	Gap 6 visit per year	-14.582912
Myanmar	2019	Gap 6 visit per year	-35.928778
Namibia	2017	Gap 6 visit per year	-1.289288

For peer review only

Nauru	2015	Gap 6 visit per year	-0.000662
Nepal	2019	Gap 6 visit per year	-14.197096
Netherlands	2018	Gap 6 visit per year	7.381198
New Zealand	2018	Gap 6 visit per year	2.636202
Nicaragua	2018	Gap 6 visit per year	0.367556
Niger	2016	Gap 6 visit per year	-10.773378
Nigeria	2018	Gap 6 visit per year	-76.884206
Niue	2008	Gap 6 visit per year	-0.000264
North Korea	2017	Gap 6 visit per year	22.751078
Norway	2019	Gap 6 visit per year	6.285688
Oman	2019	Gap 6 visit per year	-0.072944
Pakistan	2019	Gap 6 visit per year	-58.376624
Palau	2014	Gap 6 visit per year	-0.014194
Panama	2019	Gap 6 visit per year	-0.653762
Papua New Guinea	2019	Gap 6 visit per year	-4.898236
Paraguay	2018	Gap 6 visit per year	-4.701778
Peru	2018	Gap 6 visit per year	-5.280626
Philippines	2017	Gap 6 visit per year	-51.110664
Poland	2017	Gap 6 visit per year	-31.676858
Portugal	2018	Gap 6 visit per year	10.59962
Qatar	2018	Gap 6 visit per year	0.637406
Romania	2017	Gap 6 visit per year	-10.253042
Russian Federation	2018	Gap 6 visit per year	61.46006
Rwanda	2019	Gap 6 visit per year	-5.868814
Saint Kitts and Nevis	2018	Gap 6 visit per year	-0.01507
Saint Lucia	2017	Gap 6 visit per year	-0.169028
Saint Vincent and the Grenadines	2012	Gap 6 visit per year	-0.076608
Samoa	2016	Gap 6 visit per year	-0.125968
Sao Tome and Principe	2015	Gap 6 visit per year	-0.123156
Saudi Arabia	2018	Gap 6 visit per year	13.752252
Senegal	2019	Gap 6 visit per year	-9.354976
Serbia	2016	Gap 6 visit per year	-3.165038
Seychelles	2018	Gap 6 visit per year	-0.029148
Sierra Leone	2018	Gap 6 visit per year	-4.976774
Singapore	2016	Gap 6 visit per year	-0.854644
Slovakia	2018	Gap 6 visit per year	-0.192482
Slovenia	2018	Gap 6 visit per year	-0.845214
Solomon Islands	2016	Gap 6 visit per year	-0.239388
Somalia	2014	Gap 6 visit per year	-9.00537
South Africa	2019	Gap 6 visit per year	-40.08121
South Korea	2018	Gap 6 visit per year	-2.241272
Spain	2018	Gap 6 visit per year	35.699346
Sri Lanka	2019	Gap 6 visit per year	-13.88714
Sudan	2017	Gap 6 visit per year	-22.666782
Suriname	2018	Gap 6 visit per year	-0.48325

For peer review only

Swaziland	2016	Gap 6 visit per year	-0.785324
Sweden	2017	Gap 6 visit per year	7.78286
Switzerland	2018	Gap 6 visit per year	9.404384
Syrian Arab Republic	2016	Gap 6 visit per year	-3.162918
Tajikistan	2014	Gap 6 visit per year	-0.212324
Tanzania	2014	Gap 6 visit per year	-24.928026
Thailand	2019	Gap 6 visit per year	-51.925904
The Gambia	2019	Gap 6 visit per year	-1.154694
Timor-Leste	2019	Gap 6 visit per year	-0.375958
Togo	2019	Gap 6 visit per year	-4.736022
Tonga	2013	Gap 6 visit per year	-0.058162
Trinidad and Tobago	2019	Gap 6 visit per year	0.99977
Tunisia	2017	Gap 6 visit per year	-4.810848
Turkey	2018	Gap 6 visit per year	-9.865442
Turkmenistan	2014	Gap 6 visit per year	1.80547
Tuvalu	2014	Gap 6 visit per year	-0.00826
Uganda	2017	Gap 6 visit per year	-13.074792
Ukraine	2014	Gap 6 visit per year	-18.502996
United Arab Emirates	2018	Gap 6 visit per year	-0.77331
United Kingdom	2019	Gap 6 visit per year	120.999944
United States of America	2018	Gap 6 visit per year	13.508618
Uruguay	2017	Gap 6 visit per year	3.432328
Uzbekistan	2014	Gap 6 visit per year	7.116138
Vanuatu	2016	Gap 6 visit per year	-0.181126
Venezuela	2017	Gap 6 visit per year	-6.628762
Viet Nam	2016	Gap 6 visit per year	-39.501376
Yemen	2014	Gap 6 visit per year	-5.367192
Zambia	2016	Gap 6 visit per year	-6.87467
Zimbabwe	2018	Gap 6 visit per year	-8.882522
Afghanistan	2016	Gap 7 visit per year	-16.726836
Albania	2019	Gap 7 visit per year	-2.702325
Algeria	2018	Gap 7 visit per year	-8.77429
Andorra	2015	Gap 7 visit per year	0.008683
Angola	2017	Gap 7 visit per year	-15.138474
Antigua and Barbuda	2017	Gap 7 visit per year	-0.011787
Argentina	2017	Gap 7 visit per year	13.991398
Armenia	2017	Gap 7 visit per year	0.648732
Australia	2018	Gap 7 visit per year	14.255031
Austria	2018	Gap 7 visit per year	7.394126
Azerbaijan	2014	Gap 7 visit per year	3.470399
Bahamas	2017	Gap 7 visit per year	-0.227597
Bahrain	2015	Gap 7 visit per year	-1.20915
Bangladesh	2019	Gap 7 visit per year	-80.498484
Barbados	2017	Gap 7 visit per year	-0.217426
Belarus	2015	Gap 7 visit per year	2.333211

For peer review only

Belgium	2019	Gap 7 visit per year	17.033472
Belize	2018	Gap 7 visit per year	-0.177849
Benin	2019	Gap 7 visit per year	-6.219962
Bhutan	2019	Gap 7 visit per year	-0.765832
Bolivia	2017	Gap 7 visit per year	-2.863626
Bosnia and Herzegovina	2015	Gap 7 visit per year	-3.974691
Botswana	2018	Gap 7 visit per year	-2.365164
Brazil	2019	Gap 7 visit per year	-115.325989
Brunei Darussalam	2017	Gap 7 visit per year	-0.290082
Bulgaria	2018	Gap 7 visit per year	-1.989376
Burkina Faso	2019	Gap 7 visit per year	-10.786275
Burundi	2017	Gap 7 visit per year	-5.880934
Cabo Verde	2015	Gap 7 visit per year	-0.403027
Cambodia	2014	Gap 7 visit per year	-7.525296
Cameroon	2011	Gap 7 visit per year	-12.807022
Canada	2019	Gap 7 visit per year	2.887456
Central African Republic	2015	Gap 7 visit per year	-3.584589
Chad	2019	Gap 7 visit per year	-8.592803
Chile	2018	Gap 7 visit per year	22.029504
China	2017	Gap 7 visit per year	-367.448045
Colombia	2018	Gap 7 visit per year	45.008579
Comoros	2012	Gap 7 visit per year	-0.452972
Congo	2016	Gap 7 visit per year	-4.005749
Cook Islands	2014	Gap 7 visit per year	-0.016361
Costa Rica	2018	Gap 7 visit per year	0.871287
Cote d'Ivoire	2019	Gap 7 visit per year	-17.250048
Croatia	2016	Gap 7 visit per year	-4.216357
Cuba	2018	Gap 7 visit per year	26.336135
Cyprus	2016	Gap 7 visit per year	-0.657549
Czech Republic	2019	Gap 7 visit per year	-1.185558
DR Congo	2013	Gap 7 visit per year	-40.31896
Denmark	2018	Gap 7 visit per year	1.662134
Djibouti	2014	Gap 7 visit per year	-0.757378
Dominica	2018	Gap 7 visit per year	-0.084078
Dominican Republic	2019	Gap 7 visit per year	-8.421435
Ecuador	2017	Gap 7 visit per year	5.636868
Egypt	2019	Gap 7 visit per year	-61.489093
El Salvador	2018	Gap 7 visit per year	3.194179
Equatorial Guinea	2017	Gap 7 visit per year	-0.517781
Eritrea	2004	Gap 7 visit per year	-2.096562
Estonia	2017	Gap 7 visit per year	-0.277836
Ethiopia	2018	Gap 7 visit per year	-45.792356
Fiji	2015	Gap 7 visit per year	-0.626191
Finland	2018	Gap 7 visit per year	2.343355
France	2018	Gap 7 visit per year	113.133357

For peer review only - http://bmjopen.bmj.com/site/about/guidelines.xhtml

Gabon	2017	Gap 7 visit per year	-0.723587
Georgia	2019	Gap 7 visit per year	6.860271
Germany	2018	Gap 7 visit per year	36.509703
Ghana	2019	Gap 7 visit per year	-21.900082
Greece	2018	Gap 7 visit per year	15.163288
Grenada	2018	Gap 7 visit per year	-0.092256
Guatemala	2018	Gap 7 visit per year	-9.185787
Guinea	2016	Gap 7 visit per year	-7.936252
Guinea Bissau	2016	Gap 7 visit per year	-1.064383
Guyana	2018	Gap 7 visit per year	-0.217362
Haiti	2018	Gap 7 visit per year	-11.254146
Honduras	2017	Gap 7 visit per year	-5.416667
Hungary	2018	Gap 7 visit per year	-7.271038
Iceland	2019	Gap 7 visit per year	0.257353
India	2019	Gap 7 visit per year	-664.944936
Indonesia	2019	Gap 7 visit per year	-285.73102
Iran	2018	Gap 7 visit per year	-6.392893
Iraq	2018	Gap 7 visit per year	-30.498814
Ireland	2019	Gap 7 visit per year	1.184589
Israel	2019	Gap 7 visit per year	13.371686
Italy	2019	Gap 7 visit per year	126.792593
Jamaica	2016	Gap 7 visit per year	-3.488453
Japan	2018	Gap 7 visit per year	-75.236203
Jordan	2017	Gap 7 visit per year	1.987631
Kazakhstan	2014	Gap 7 visit per year	10.572514
Kenya	2018	Gap 7 visit per year	-26.943346
Kiribati	2013	Gap 7 visit per year	-0.084347
Kuwait	2015	Gap 7 visit per year	0.418299
Kyrgyzstan	2014	Gap 7 visit per year	0.383209
Lao PDR	2017	Gap 7 visit per year	-3.422633
Latvia	2018	Gap 7 visit per year	-1.117965
Lebanon	2018	Gap 7 visit per year	0.853844
Lesotho	2010	Gap 7 visit per year	-1.70522
Liberia	2015	Gap 7 visit per year	-3.345034
Libya	2017	Gap 7 visit per year	-1.589855
Lithuania	2019	Gap 7 visit per year	0.297616
Luxembourg	2017	Gap 7 visit per year	0.016708
Macedonia (TFYR)	2015	Gap 7 visit per year	-1.368915
Madagascar	2014	Gap 7 visit per year	-14.792126
Malawi	2018	Gap 7 visit per year	-8.35228
Malaysia	2015	Gap 7 visit per year	-13.472741
Maldives	2018	Gap 7 visit per year	0.002475
Mali	2018	Gap 7 visit per year	-10.037862
Malta	2015	Gap 7 visit per year	-0.093769
Marshall Islands	2012	Gap 7 visit per year	-0.029818

For peer review only

Mauritania	2018	Gap 7 visit per year	-2.483069
Mauritius	2018	Gap 7 visit per year	-0.309745
Mexico	2018	Gap 7 visit per year	178.163523
Micronesia (Federated States of)	2009	Gap 7 visit per year	-0.063696
Moldova	2019	Gap 7 visit per year	-2.900292
Mongolia	2018	Gap 7 visit per year	1.866538
Montenegro	2018	Gap 7 visit per year	-0.42881
Morocco	2017	Gap 7 visit per year	-27.174398
Mozambique	2019	Gap 7 visit per year	-17.227564
Myanmar	2019	Gap 7 visit per year	-45.235741
Namibia	2017	Gap 7 visit per year	-1.622586
Nauru	2015	Gap 7 visit per year	-0.001939
Nepal	2019	Gap 7 visit per year	-18.492112
Netherlands	2018	Gap 7 visit per year	3.341981
New Zealand	2018	Gap 7 visit per year	1.723569
Nicaragua	2018	Gap 7 visit per year	-0.468018
Niger	2016	Gap 7 visit per year	-12.643941
Nigeria	2018	Gap 7 visit per year	-95.910157
Niue	2008	Gap 7 visit per year	-0.000558
North Korea	2017	Gap 7 visit per year	18.737341
Norway	2019	Gap 7 visit per year	5.143636
Oman	2019	Gap 7 visit per year	-0.885268
Pakistan	2019	Gap 7 visit per year	-88.280978
Palau	2014	Gap 7 visit per year	-0.018643
Panama	2019	Gap 7 visit per year	-1.340389
Papua New Guinea	2019	Gap 7 visit per year	-5.762942
Paraguay	2018	Gap 7 visit per year	-6.270491
Peru	2018	Gap 7 visit per year	-8.346647
Philippines	2017	Gap 7 visit per year	-64.890858
Poland	2017	Gap 7 visit per year	-44.480001
Portugal	2018	Gap 7 visit per year	7.82639
Qatar	2018	Gap 7 visit per year	0.167557
Romania	2017	Gap 7 visit per year	-16.843799
Russian Federation	2018	Gap 7 visit per year	17.78732
Rwanda	2019	Gap 7 visit per year	-6.971283
Saint Kitts and Nevis	2018	Gap 7 visit per year	-0.029665
Saint Lucia	2017	Gap 7 visit per year	-0.206866
Saint Vincent and the Grenadines	2012	Gap 7 visit per year	-0.097876
Samoa	2016	Gap 7 visit per year	-0.152546
Sao Tome and Principe	2015	Gap 7 visit per year	-0.148932
Saudi Arabia	2018	Gap 7 visit per year	8.709044
Senegal	2019	Gap 7 visit per year	-11.033722
Serbia	2016	Gap 7 visit per year	-5.989461
Seychelles	2018	Gap 7 visit per year	-0.054006
Sierra Leone	2018	Gap 7 visit per year	-5.853403

For peer review only

For peer review only - http://bmjopen.bmj.com/site/about/guidelines.xhtml

Singapore	2016	Gap 7 visit per year	-2.077668
Slovakia	2018	Gap 7 visit per year	-1.822729
Slovenia	2018	Gap 7 visit per year	-1.535333
Solomon Islands	2016	Gap 7 visit per year	-0.289286
Somalia	2014	Gap 7 visit per year	-10.532015
South Africa	2019	Gap 7 visit per year	-50.627495
South Korea	2018	Gap 7 visit per year	-12.883984
Spain	2018	Gap 7 visit per year	25.968737
Sri Lanka	2019	Gap 7 visit per year	-18.25208
Sudan	2017	Gap 7 visit per year	-27.334829
Suriname	2018	Gap 7 visit per year	-0.603125
Swaziland	2016	Gap 7 visit per year	-0.925128
Sweden	2017	Gap 7 visit per year	5.50517
Switzerland	2018	Gap 7 visit per year	7.893448
Syrian Arab Republic	2016	Gap 7 visit per year	-5.563821
Tajikistan	2014	Gap 7 visit per year	-1.432628
Tanzania	2014	Gap 7 visit per year	-29.333947
Thailand	2019	Gap 7 visit per year	-65.911388
The Gambia	2019	Gap 7 visit per year	-1.368143
Timor-Leste	2019	Gap 7 visit per year	-0.521451
Togo	2019	Gap 7 visit per year	-5.577609
Tonga	2013	Gap 7 visit per year	-0.072439
Trinidad and Tobago	2019	Gap 7 visit per year	0.646065
Tunisia	2017	Gap 7 visit per year	-6.853656
Turkey	2018	Gap 7 visit per year	-23.905599
Turkmenistan	2014	Gap 7 visit per year	1.092965
Tuvalu	2014	Gap 7 visit per year	-0.01047
Uganda	2017	Gap 7 visit per year	-15.830424
Ukraine	2014	Gap 7 visit per year	-32.835662
United Arab Emirates	2018	Gap 7 visit per year	-2.930945
United Kingdom	2019	Gap 7 visit per year	108.396018
United States of America	2018	Gap 7 visit per year	-55.210029
Uruguay	2017	Gap 7 visit per year	2.589716
Uzbekistan	2014	Gap 7 visit per year	2.282411
Vanuatu	2016	Gap 7 visit per year	-0.215147
Venezuela	2017	Gap 7 visit per year	-11.972389
Viet Nam	2016	Gap 7 visit per year	-52.546522
Yemen	2014	Gap 7 visit per year	-7.391724
Zambia	2016	Gap 7 visit per year	-8.146615
Zimbabwe	2018	Gap 7 visit per year	-10.615109
Afghanistan	2016	Gap 8 visit per year	-19.819384
Albania	2019	Gap 8 visit per year	-3.4273
Algeria	2018	Gap 8 visit per year	-15.21376
Andorra	2015	Gap 8 visit per year	-0.008648
Angola	2017	Gap 8 visit per year	-17.758256

For peer review only - http://bmjopen.bmj.com/site/about/guidelines.xhtml

Antigua and Barbuda	2017	Gap 8 visit per year	-0.032328
Argentina	2017	Gap 8 visit per year	3.467812
Armenia	2017	Gap 8 visit per year	-0.184592
Australia	2018	Gap 8 visit per year	9.605464
Austria	2018	Gap 8 visit per year	5.140644
Azerbaijan	2014	Gap 8 visit per year	1.626456
Bahamas	2017	Gap 8 visit per year	-0.312968
Bahrain	2015	Gap 8 visit per year	-1.4726
Bangladesh	2019	Gap 8 visit per year	-99.413196
Barbados	2017	Gap 8 visit per year	-0.299344
Belarus	2015	Gap 8 visit per year	-0.833116
Belgium	2019	Gap 8 visit per year	14.557968
Belize	2018	Gap 8 visit per year	-0.232756
Benin	2019	Gap 8 visit per year	-7.163028
Bhutan	2019	Gap 8 visit per year	-0.900308
Bolivia	2017	Gap 8 visit per year	-4.096144
Bosnia and Herzegovina	2015	Gap 8 visit per year	-5.072004
Botswana	2018	Gap 8 visit per year	-2.749616
Brazil	2019	Gap 8 visit per year	-166.633916
Brunei Darussalam	2017	Gap 8 visit per year	-0.380308
Bulgaria	2018	Gap 8 visit per year	-4.392644
Burkina Faso	2019	Gap 8 visit per year	-12.4636
Burundi	2017	Gap 8 visit per year	-6.798496
Cabo Verde	2015	Gap 8 visit per year	-0.489888
Cambodia	2014	Gap 8 visit per year	-8.810624
Cameroon	2011	Gap 8 visit per year	-14.768168
Canada	2019	Gap 8 visit per year	-3.226836
Central African Republic	2015	Gap 8 visit per year	-4.119816
Chad	2019	Gap 8 visit per year	-9.882132
Chile	2018	Gap 8 visit per year	18.243576
China	2017	Gap 8 visit per year	-622.01198
Colombia	2018	Gap 8 visit per year	37.802376
Comoros	2012	Gap 8 visit per year	-0.526468
Congo	2016	Gap 8 visit per year	-4.615856
Cook Islands	2014	Gap 8 visit per year	-0.020484
Costa Rica	2018	Gap 8 visit per year	-0.037672
Cote d'Ivoire	2019	Gap 8 visit per year	-20.012412
Croatia	2016	Gap 8 visit per year	-5.720408
Cuba	2018	Gap 8 visit per year	23.27944
Cyprus	2016	Gap 8 visit per year	-0.914556
Czech Republic	2019	Gap 8 visit per year	-4.497352
DR Congo	2013	Gap 8 visit per year	-46.53724
Denmark	2018	Gap 8 visit per year	0.163796
Djibouti	2014	Gap 8 visit per year	-0.879932
Dominica	2018	Gap 8 visit per year	-0.101732

For peer review only - http://bmjopen.bmj.com/site/about/guidelines.xhtml

Dominican Republic	2019	Gap 8 visit per year	-10.73814
Ecuador	2017	Gap 8 visit per year	3.780492
Egypt	2019	Gap 8 visit per year	-75.624892
El Salvador	2018	Gap 8 visit per year	2.334276
Equatorial Guinea	2017	Gap 8 visit per year	-0.627964
Eritrea	2004	Gap 8 visit per year	-2.411428
Estonia	2017	Gap 8 visit per year	-0.643884
Ethiopia	2018	Gap 8 visit per year	-52.933764
Fiji	2015	Gap 8 visit per year	-0.769004
Finland	2018	Gap 8 visit per year	0.84762
France	2018	Gap 8 visit per year	98.961408
Gabon	2017	Gap 8 visit per year	-0.927528
Georgia	2019	Gap 8 visit per year	5.819524
Germany	2018	Gap 8 visit per year	16.196732
Ghana	2019	Gap 8 visit per year	-25.259808
Greece	2018	Gap 8 visit per year	12.649972
Grenada	2018	Gap 8 visit per year	-0.116864
Guatemala	2018	Gap 8 visit per year	-10.935328
Guinea	2016	Gap 8 visit per year	-9.139788
Guinea Bissau	2016	Gap 8 visit per year	-1.232652
Guyana	2018	Gap 8 visit per year	-0.349628
Haiti	2018	Gap 8 visit per year	-13.048024
Honduras	2017	Gap 8 visit per year	-6.398548
Hungary	2018	Gap 8 visit per year	-10.672472
Iceland	2019	Gap 8 visit per year	0.193832
India	2019	Gap 8 visit per year	-850.520784
Indonesia	2019	Gap 8 visit per year	-335.53988
Iran	2018	Gap 8 visit per year	-16.563592
Iraq	2018	Gap 8 visit per year	-36.799216
Ireland	2019	Gap 8 visit per year	0.184816
Israel	2019	Gap 8 visit per year	11.955284
Italy	2019	Gap 8 visit per year	110.249392
Jamaica	2016	Gap 8 visit per year	-4.081232
Japan	2018	Gap 8 visit per year	-108.513232
Jordan	2017	Gap 8 visit per year	0.647364
Kazakhstan	2014	Gap 8 visit per year	7.164016
Kenya	2018	Gap 8 visit per year	-31.366824
Kiribati	2013	Gap 8 visit per year	-0.097968
Kuwait	2015	Gap 8 visit per year	-0.246944
Kyrgyzstan	2014	Gap 8 visit per year	-0.485904
Lao PDR	2017	Gap 8 visit per year	-4.096652
Latvia	2018	Gap 8 visit per year	-1.73246
Lebanon	2018	Gap 8 visit per year	-0.054964
Lesotho	2010	Gap 8 visit per year	-1.95868
Liberia	2015	Gap 8 visit per year	-3.834896

For peer review only - http://bmjopen.bmj.com/site/about/guidelines.xhtml

Libya	2017	Gap 8 visit per year	-2.79962
Lithuania	2019	Gap 8 visit per year	-0.653296
Luxembourg	2017	Gap 8 visit per year	-0.108048
Macedonia (TFYR)	2015	Gap 8 visit per year	-1.99126
Madagascar	2014	Gap 8 visit per year	-17.210644
Malawi	2018	Gap 8 visit per year	-9.59182
Malaysia	2015	Gap 8 visit per year	-18.718204
Maldives	2018	Gap 8 visit per year	-0.0601
Mali	2018	Gap 8 visit per year	-11.647128
Malta	2015	Gap 8 visit per year	-0.195736
Marshall Islands	2012	Gap 8 visit per year	-0.035792
Mauritania	2018	Gap 8 visit per year	-2.896436
Mauritius	2018	Gap 8 visit per year	-0.58328
Mexico	2018	Gap 8 visit per year	159.903312
Micronesia (Federated States of)	2009	Gap 8 visit per year	-0.074224
Moldova	2019	Gap 8 visit per year	-4.053548
Mongolia	2018	Gap 8 visit per year	1.260972
Montenegro	2018	Gap 8 visit per year	-0.61364
Morocco	2017	Gap 8 visit per year	-32.913812
Mozambique	2019	Gap 8 visit per year	-19.872216
Myanmar	2019	Gap 8 visit per year	-54.542704
Namibia	2017	Gap 8 visit per year	-1.955884
Nauru	2015	Gap 8 visit per year	-0.003216
Nepal	2019	Gap 8 visit per year	-22.787128
Netherlands	2018	Gap 8 visit per year	-0.697236
New Zealand	2018	Gap 8 visit per year	0.810936
Nicaragua	2018	Gap 8 visit per year	-1.303592
Niger	2016	Gap 8 visit per year	-14.514504
Nigeria	2018	Gap 8 visit per year	-114.936108
Niue	2008	Gap 8 visit per year	-0.000852
North Korea	2017	Gap 8 visit per year	14.723604
Norway	2019	Gap 8 visit per year	4.001584
Oman	2019	Gap 8 visit per year	-1.697592
Pakistan	2019	Gap 8 visit per year	-118.185332
Palau	2014	Gap 8 visit per year	-0.023092
Panama	2019	Gap 8 visit per year	-2.027016
Papua New Guinea	2019	Gap 8 visit per year	-6.627648
Paraguay	2018	Gap 8 visit per year	-7.839204
Peru	2018	Gap 8 visit per year	-11.412668
Philippines	2017	Gap 8 visit per year	-78.671052
Poland	2017	Gap 8 visit per year	-57.283144
Portugal	2018	Gap 8 visit per year	5.05316
Qatar	2018	Gap 8 visit per year	-0.302292
Romania	2017	Gap 8 visit per year	-23.434556
Russian Federation	2018	Gap 8 visit per year	-25.88542

For peer review only

Rwanda	2019	Gap 8 visit per year	-8.073752
Saint Kitts and Nevis	2018	Gap 8 visit per year	-0.04426
Saint Lucia	2017	Gap 8 visit per year	-0.244704
Saint Vincent and the Grenadines	2012	Gap 8 visit per year	-0.119144
Samoa	2016	Gap 8 visit per year	-0.179124
Sao Tome and Principe	2015	Gap 8 visit per year	-0.174708
Saudi Arabia	2018	Gap 8 visit per year	3.665836
Senegal	2019	Gap 8 visit per year	-12.712468
Serbia	2016	Gap 8 visit per year	-8.813884
Seychelles	2018	Gap 8 visit per year	-0.078864
Sierra Leone	2018	Gap 8 visit per year	-6.730032
Singapore	2016	Gap 8 visit per year	-3.300692
Slovakia	2018	Gap 8 visit per year	-3.452976
Slovenia	2018	Gap 8 visit per year	-2.225452
Solomon Islands	2016	Gap 8 visit per year	-0.339184
Somalia	2014	Gap 8 visit per year	-12.05866
South Africa	2019	Gap 8 visit per year	-61.17378
South Korea	2018	Gap 8 visit per year	-23.526696
Spain	2018	Gap 8 visit per year	16.238128
Sri Lanka	2019	Gap 8 visit per year	-22.61702
Sudan	2017	Gap 8 visit per year	-32.002876
Suriname	2018	Gap 8 visit per year	-0.723
Swaziland	2016	Gap 8 visit per year	-1.064932
Sweden	2017	Gap 8 visit per year	3.22748
Switzerland	2018	Gap 8 visit per year	6.382512
Syrian Arab Republic	2016	Gap 8 visit per year	-7.964724
Tajikistan	2014	Gap 8 visit per year	-2.652932
Tanzania	2014	Gap 8 visit per year	-33.739868
Thailand	2019	Gap 8 visit per year	-79.896872
The Gambia	2019	Gap 8 visit per year	-1.581592
Timor-Leste	2019	Gap 8 visit per year	-0.666944
Togo	2019	Gap 8 visit per year	-6.419196
Tonga	2013	Gap 8 visit per year	-0.086716
Trinidad and Tobago	2019	Gap 8 visit per year	0.29236
Tunisia	2017	Gap 8 visit per year	-8.896464
Turkey	2018	Gap 8 visit per year	-37.945756
Turkmenistan	2014	Gap 8 visit per year	0.38046
Tuvalu	2014	Gap 8 visit per year	-0.01268
Uganda	2017	Gap 8 visit per year	-18.586056
Ukraine	2014	Gap 8 visit per year	-47.168328
United Arab Emirates	2018	Gap 8 visit per year	-5.08858
United Kingdom	2019	Gap 8 visit per year	95.792092
United States of America	2018	Gap 8 visit per year	-123.928676
Uruguay	2017	Gap 8 visit per year	1.747104
Uzbekistan	2014	Gap 8 visit per year	-2.551316

For peer review only

Vanuatu	2016	Gap 8 visit per year	-0.249168
Venezuela	2017	Gap 8 visit per year	-17.316016
Viet Nam	2016	Gap 8 visit per year	-65.591668
Yemen	2014	Gap 8 visit per year	-9.416256
Zambia	2016	Gap 8 visit per year	-9.41856
Zimbabwe	2018	Gap 8 visit per year	-12.347696
Afghanistan	2016	Gap 9 visit per year	-22.911932
Albania	2019	Gap 9 visit per year	-4.152275
Algeria	2018	Gap 9 visit per year	-21.65323
Andorra	2015	Gap 9 visit per year	-0.025979
Angola	2017	Gap 9 visit per year	-20.378038
Antigua and Barbuda	2017	Gap 9 visit per year	-0.052869
Argentina	2017	Gap 9 visit per year	-7.055774
Armenia	2017	Gap 9 visit per year	-1.017916
Australia	2018	Gap 9 visit per year	4.955897
Austria	2018	Gap 9 visit per year	2.887162
Azerbaijan	2014	Gap 9 visit per year	-0.217487
Bahamas	2017	Gap 9 visit per year	-0.398339
Bahrain	2015	Gap 9 visit per year	-1.73605
Bangladesh	2019	Gap 9 visit per year	-118.327908
Barbados	2017	Gap 9 visit per year	-0.381262
Belarus	2015	Gap 9 visit per year	-3.999443
Belgium	2019	Gap 9 visit per year	12.082464
Belize	2018	Gap 9 visit per year	-0.287663
Benin	2019	Gap 9 visit per year	-8.106094
Bhutan	2019	Gap 9 visit per year	-1.034784
Bolivia	2017	Gap 9 visit per year	-5.328662
Bosnia and Herzegovina	2015	Gap 9 visit per year	-6.169317
Botswana	2018	Gap 9 visit per year	-3.134068
Brazil	2019	Gap 9 visit per year	-217.941843
Brunei Darussalam	2017	Gap 9 visit per year	-0.470534
Bulgaria	2018	Gap 9 visit per year	-6.795912
Burkina Faso	2019	Gap 9 visit per year	-14.140925
Burundi	2017	Gap 9 visit per year	-7.716058
Cabo Verde	2015	Gap 9 visit per year	-0.576749
Cambodia	2014	Gap 9 visit per year	-10.095952
Cameroon	2011	Gap 9 visit per year	-16.729314
Canada	2019	Gap 9 visit per year	-9.341128
Central African Republic	2015	Gap 9 visit per year	-4.655043
Chad	2019	Gap 9 visit per year	-11.171461
Chile	2018	Gap 9 visit per year	14.457648
China	2017	Gap 9 visit per year	-876.575915
Colombia	2018	Gap 9 visit per year	30.596173
Comoros	2012	Gap 9 visit per year	-0.599964
Congo	2016	Gap 9 visit per year	-5.225963

For peer review only - http://bmjopen.bmj.com/site/about/guidelines.xhtml

For peer review only

Cook Islands	2014	Gap 9 visit per year	-0.024607
Costa Rica	2018	Gap 9 visit per year	-0.946631
Cote d'Ivoire	2019	Gap 9 visit per year	-22.774776
Croatia	2016	Gap 9 visit per year	-7.224459
Cuba	2018	Gap 9 visit per year	20.222745
Cyprus	2016	Gap 9 visit per year	-1.171563
Czech Republic	2019	Gap 9 visit per year	-7.809146
DR Congo	2013	Gap 9 visit per year	-52.75552
Denmark	2018	Gap 9 visit per year	-1.334542
Djibouti	2014	Gap 9 visit per year	-1.002486
Dominica	2018	Gap 9 visit per year	-0.119386
Dominican Republic	2019	Gap 9 visit per year	-13.054845
Ecuador	2017	Gap 9 visit per year	1.924116
Egypt	2019	Gap 9 visit per year	-89.760691
El Salvador	2018	Gap 9 visit per year	1.474373
Equatorial Guinea	2017	Gap 9 visit per year	-0.738147
Eritrea	2004	Gap 9 visit per year	-2.726294
Estonia	2017	Gap 9 visit per year	-1.009932
Ethiopia	2018	Gap 9 visit per year	-60.075172
Fiji	2015	Gap 9 visit per year	-0.911817
Finland	2018	Gap 9 visit per year	-0.648115
France	2018	Gap 9 visit per year	84.789459
Gabon	2017	Gap 9 visit per year	-1.131469
Georgia	2019	Gap 9 visit per year	4.778777
Germany	2018	Gap 9 visit per year	-4.116239
Ghana	2019	Gap 9 visit per year	-28.619534
Greece	2018	Gap 9 visit per year	10.136656
Grenada	2018	Gap 9 visit per year	-0.141472
Guatemala	2018	Gap 9 visit per year	-12.684869
Guinea	2016	Gap 9 visit per year	-10.343324
Guinea Bissau	2016	Gap 9 visit per year	-1.400921
Guyana	2018	Gap 9 visit per year	-0.481894
Haiti	2018	Gap 9 visit per year	-14.841902
Honduras	2017	Gap 9 visit per year	-7.380429
Hungary	2018	Gap 9 visit per year	-14.073906
Iceland	2019	Gap 9 visit per year	0.130311
India	2019	Gap 9 visit per year	-1036.096632
Indonesia	2019	Gap 9 visit per year	-385.34874
Iran	2018	Gap 9 visit per year	-26.734291
Iraq	2018	Gap 9 visit per year	-43.099618
Ireland	2019	Gap 9 visit per year	-0.814957
Israel	2019	Gap 9 visit per year	10.538882
Italy	2019	Gap 9 visit per year	93.706191
Jamaica	2016	Gap 9 visit per year	-4.674011
Japan	2018	Gap 9 visit per year	-141.790261

For peer review only

Jordan	2017	Gap 9 visit per year	-0.692903
Kazakhstan	2014	Gap 9 visit per year	3.755518
Kenya	2018	Gap 9 visit per year	-35.790302
Kiribati	2013	Gap 9 visit per year	-0.111589
Kuwait	2015	Gap 9 visit per year	-0.912187
Kyrgyzstan	2014	Gap 9 visit per year	-1.355017
Lao PDR	2017	Gap 9 visit per year	-4.770671
Latvia	2018	Gap 9 visit per year	-2.346955
Lebanon	2018	Gap 9 visit per year	-0.963772
Lesotho	2010	Gap 9 visit per year	-2.21214
Liberia	2015	Gap 9 visit per year	-4.324758
Libya	2017	Gap 9 visit per year	-4.009385
Lithuania	2019	Gap 9 visit per year	-1.604208
Luxembourg	2017	Gap 9 visit per year	-0.232804
Macedonia (TFYR)	2015	Gap 9 visit per year	-2.613605
Madagascar	2014	Gap 9 visit per year	-19.629162
Malawi	2018	Gap 9 visit per year	-10.83136
Malaysia	2015	Gap 9 visit per year	-23.963667
Maldives	2018	Gap 9 visit per year	-0.122675
Mali	2018	Gap 9 visit per year	-13.256394
Malta	2015	Gap 9 visit per year	-0.297703
Marshall Islands	2012	Gap 9 visit per year	-0.041766
Mauritania	2018	Gap 9 visit per year	-3.309803
Mauritius	2018	Gap 9 visit per year	-0.856815
Mexico	2018	Gap 9 visit per year	141.643101
Micronesia (Federated States of)	2009	Gap 9 visit per year	-0.084752
Moldova	2019	Gap 9 visit per year	-5.206804
Mongolia	2018	Gap 9 visit per year	0.655406
Montenegro	2018	Gap 9 visit per year	-0.79847
Morocco	2017	Gap 9 visit per year	-38.653226
Mozambique	2019	Gap 9 visit per year	-22.516868
Myanmar	2019	Gap 9 visit per year	-63.849667
Namibia	2017	Gap 9 visit per year	-2.289182
Nauru	2015	Gap 9 visit per year	-0.004493
Nepal	2019	Gap 9 visit per year	-27.082144
Netherlands	2018	Gap 9 visit per year	-4.736453
New Zealand	2018	Gap 9 visit per year	-0.101697
Nicaragua	2018	Gap 9 visit per year	-2.139166
Niger	2016	Gap 9 visit per year	-16.385067
Nigeria	2018	Gap 9 visit per year	-133.962059
Niue	2008	Gap 9 visit per year	-0.001146
North Korea	2017	Gap 9 visit per year	10.709867
Norway	2019	Gap 9 visit per year	2.859532
Oman	2019	Gap 9 visit per year	-2.509916
Pakistan	2019	Gap 9 visit per year	-148.089686

For peer review only

Palau	2014	Gap 9 visit per year	-0.027541
Panama	2019	Gap 9 visit per year	-2.713643
Papua New Guinea	2019	Gap 9 visit per year	-7.492354
Paraguay	2018	Gap 9 visit per year	-9.407917
Peru	2018	Gap 9 visit per year	-14.478689
Philippines	2017	Gap 9 visit per year	-92.451246
Poland	2017	Gap 9 visit per year	-70.086287
Portugal	2018	Gap 9 visit per year	2.27993
Qatar	2018	Gap 9 visit per year	-0.772141
Romania	2017	Gap 9 visit per year	-30.025313
Russian Federation	2018	Gap 9 visit per year	-69.55816
Rwanda	2019	Gap 9 visit per year	-9.176221
Saint Kitts and Nevis	2018	Gap 9 visit per year	-0.058855
Saint Lucia	2017	Gap 9 visit per year	-0.282542
Saint Vincent and the Grenadines	2012	Gap 9 visit per year	-0.140412
Samoa	2016	Gap 9 visit per year	-0.205702
Sao Tome and Principe	2015	Gap 9 visit per year	-0.200484
Saudi Arabia	2018	Gap 9 visit per year	-1.377372
Senegal	2019	Gap 9 visit per year	-14.391214
Serbia	2016	Gap 9 visit per year	-11.638307
Seychelles	2018	Gap 9 visit per year	-0.103722
Sierra Leone	2018	Gap 9 visit per year	-7.606661
Singapore	2016	Gap 9 visit per year	-4.523716
Slovakia	2018	Gap 9 visit per year	-5.083223
Slovenia	2018	Gap 9 visit per year	-2.915571
Solomon Islands	2016	Gap 9 visit per year	-0.389082
Somalia	2014	Gap 9 visit per year	-13.585305
South Africa	2019	Gap 9 visit per year	-71.720065
South Korea	2018	Gap 9 visit per year	-34.169408
Spain	2018	Gap 9 visit per year	6.507519
Sri Lanka	2019	Gap 9 visit per year	-26.98196
Sudan	2017	Gap 9 visit per year	-36.670923
Suriname	2018	Gap 9 visit per year	-0.842875
Swaziland	2016	Gap 9 visit per year	-1.204736
Sweden	2017	Gap 9 visit per year	0.94979
Switzerland	2018	Gap 9 visit per year	4.871576
Syrian Arab Republic	2016	Gap 9 visit per year	-10.365627
Tajikistan	2014	Gap 9 visit per year	-3.873236
Tanzania	2014	Gap 9 visit per year	-38.145789
Thailand	2019	Gap 9 visit per year	-93.882356
The Gambia	2019	Gap 9 visit per year	-1.795041
Timor-Leste	2019	Gap 9 visit per year	-0.812437
Togo	2019	Gap 9 visit per year	-7.260783
Tonga	2013	Gap 9 visit per year	-0.100993
Trinidad and Tobago	2019	Gap 9 visit per year	-0.061345

For peer review only

Tunisia	2017	Gap 9 visit per year	-10.939272
Turkey	2018	Gap 9 visit per year	-51.985913
Turkmenistan	2014	Gap 9 visit per year	-0.332045
Tuvalu	2014	Gap 9 visit per year	-0.01489
Uganda	2017	Gap 9 visit per year	-21.341688
Ukraine	2014	Gap 9 visit per year	-61.500994
United Arab Emirates	2018	Gap 9 visit per year	-7.246215
United Kingdom	2019	Gap 9 visit per year	83.188166
United States of America	2018	Gap 9 visit per year	-192.647323
Uruguay	2017	Gap 9 visit per year	0.904492
Uzbekistan	2014	Gap 9 visit per year	-7.385043
Vanuatu	2016	Gap 9 visit per year	-0.283189
Venezuela	2017	Gap 9 visit per year	-22.659643
Viet Nam	2016	Gap 9 visit per year	-78.636814
Yemen	2014	Gap 9 visit per year	-11.440788
Zambia	2016	Gap 9 visit per year	-10.690505
Zimbabwe	2018	Gap 9 visit per year	-14.080283
Afghanistan	2016	Gap 10 visit per year	-26.00448
Albania	2019	Gap 10 visit per year	-4.87725
Algeria	2018	Gap 10 visit per year	-28.0927
Andorra	2015	Gap 10 visit per year	-0.04331
Angola	2017	Gap 10 visit per year	-22.99782
Antigua and Barbuda	2017	Gap 10 visit per year	-0.07341
Argentina	2017	Gap 10 visit per year	-17.57936
Armenia	2017	Gap 10 visit per year	-1.85124
Australia	2018	Gap 10 visit per year	0.30633
Austria	2018	Gap 10 visit per year	0.63368
Azerbaijan	2014	Gap 10 visit per year	-2.06143
Bahamas	2017	Gap 10 visit per year	-0.48371
Bahrain	2015	Gap 10 visit per year	-1.9995
Bangladesh	2019	Gap 10 visit per year	-137.24262
Barbados	2017	Gap 10 visit per year	-0.46318
Belarus	2015	Gap 10 visit per year	-7.16577
Belgium	2019	Gap 10 visit per year	9.60696
Belize	2018	Gap 10 visit per year	-0.34257
Benin	2019	Gap 10 visit per year	-9.04916
Bhutan	2019	Gap 10 visit per year	-1.16926
Bolivia	2017	Gap 10 visit per year	-6.56118
Bosnia and Herzegovina	2015	Gap 10 visit per year	-7.26663
Botswana	2018	Gap 10 visit per year	-3.51852
Brazil	2019	Gap 10 visit per year	-269.24977
Brunei Darussalam	2017	Gap 10 visit per year	-0.56076
Bulgaria	2018	Gap 10 visit per year	-9.19918
Burkina Faso	2019	Gap 10 visit per year	-15.81825
Burundi	2017	Gap 10 visit per year	-8.63362

For peer review only

For peer review only - http://bmjopen.bmj.com/site/about/guidelines.xhtml

Cabo Verde	2015	Gap 10 visit per year	-0.66361
Cambodia	2014	Gap 10 visit per year	-11.38128
Cameroon	2011	Gap 10 visit per year	-18.69046
Canada	2019	Gap 10 visit per year	-15.45542
Central African Republic	2015	Gap 10 visit per year	-5.19027
Chad	2019	Gap 10 visit per year	-12.46079
Chile	2018	Gap 10 visit per year	10.67172
China	2017	Gap 10 visit per year	-1131.13985
Colombia	2018	Gap 10 visit per year	23.38997
Comoros	2012	Gap 10 visit per year	-0.67346
Congo	2016	Gap 10 visit per year	-5.83607
Cook Islands	2014	Gap 10 visit per year	-0.02873
Costa Rica	2018	Gap 10 visit per year	-1.85559
Cote d'Ivoire	2019	Gap 10 visit per year	-25.53714
Croatia	2016	Gap 10 visit per year	-8.72851
Cuba	2018	Gap 10 visit per year	17.16605
Cyprus	2016	Gap 10 visit per year	-1.42857
Czech Republic	2019	Gap 10 visit per year	-11.12094
DR Congo	2013	Gap 10 visit per year	-58.9738
Denmark	2018	Gap 10 visit per year	-2.83288
Djibouti	2014	Gap 10 visit per year	-1.12504
Dominica	2018	Gap 10 visit per year	-0.13704
Dominican Republic	2019	Gap 10 visit per year	-15.37155
Ecuador	2017	Gap 10 visit per year	0.06774
Egypt	2019	Gap 10 visit per year	-103.89649
El Salvador	2018	Gap 10 visit per year	0.61447
Equatorial Guinea	2017	Gap 10 visit per year	-0.84833
Eritrea	2004	Gap 10 visit per year	-3.04116
Estonia	2017	Gap 10 visit per year	-1.37598
Ethiopia	2018	Gap 10 visit per year	-67.21658
Fiji	2015	Gap 10 visit per year	-1.05463
Finland	2018	Gap 10 visit per year	-2.14385
France	2018	Gap 10 visit per year	70.61751
Gabon	2017	Gap 10 visit per year	-1.33541
Georgia	2019	Gap 10 visit per year	3.73803
Germany	2018	Gap 10 visit per year	-24.42921
Ghana	2019	Gap 10 visit per year	-31.97926
Greece	2018	Gap 10 visit per year	7.62334
Grenada	2018	Gap 10 visit per year	-0.16608
Guatemala	2018	Gap 10 visit per year	-14.43441
Guinea	2016	Gap 10 visit per year	-11.54686
Guinea Bissau	2016	Gap 10 visit per year	-1.56919
Guyana	2018	Gap 10 visit per year	-0.61416
Haiti	2018	Gap 10 visit per year	-16.63578
Honduras	2017	Gap 10 visit per year	-8.36231

For peer review only

Hungary	2018	Gap 10 visit per year	-17.47534
Iceland	2019	Gap 10 visit per year	0.06679
India	2019	Gap 10 visit per year	-1221.67248
Indonesia	2019	Gap 10 visit per year	-435.1576
Iran	2018	Gap 10 visit per year	-36.90499
Iraq	2018	Gap 10 visit per year	-49.40002
Ireland	2019	Gap 10 visit per year	-1.81473
Israel	2019	Gap 10 visit per year	9.12248
Italy	2019	Gap 10 visit per year	77.16299
Jamaica	2016	Gap 10 visit per year	-5.26679
Japan	2018	Gap 10 visit per year	-175.06729
Jordan	2017	Gap 10 visit per year	-2.03317
Kazakhstan	2014	Gap 10 visit per year	0.34702
Kenya	2018	Gap 10 visit per year	-40.21378
Kiribati	2013	Gap 10 visit per year	-0.12521
Kuwait	2015	Gap 10 visit per year	-1.57743
Kyrgyzstan	2014	Gap 10 visit per year	-2.22413
Lao PDR	2017	Gap 10 visit per year	-5.44469
Latvia	2018	Gap 10 visit per year	-2.96145
Lebanon	2018	Gap 10 visit per year	-1.87258
Lesotho	2010	Gap 10 visit per year	-2.4656
Liberia	2015	Gap 10 visit per year	-4.81462
Libya	2017	Gap 10 visit per year	-5.21915
Lithuania	2019	Gap 10 visit per year	-2.55512
Luxembourg	2017	Gap 10 visit per year	-0.35756
Macedonia (TFYR)	2015	Gap 10 visit per year	-3.23595
Madagascar	2014	Gap 10 visit per year	-22.04768
Malawi	2018	Gap 10 visit per year	-12.0709
Malaysia	2015	Gap 10 visit per year	-29.20913
Maldives	2018	Gap 10 visit per year	-0.18525
Mali	2018	Gap 10 visit per year	-14.86566
Malta	2015	Gap 10 visit per year	-0.39967
Marshall Islands	2012	Gap 10 visit per year	-0.04774
Mauritania	2018	Gap 10 visit per year	-3.72317
Mauritius	2018	Gap 10 visit per year	-1.13035
Mexico	2018	Gap 10 visit per year	123.38289
Micronesia (Federated States of)	2009	Gap 10 visit per year	-0.09528
Moldova	2019	Gap 10 visit per year	-6.36006
Mongolia	2018	Gap 10 visit per year	0.04984
Montenegro	2018	Gap 10 visit per year	-0.9833
Morocco	2017	Gap 10 visit per year	-44.39264
Mozambique	2019	Gap 10 visit per year	-25.16152
Myanmar	2019	Gap 10 visit per year	-73.15663
Namibia	2017	Gap 10 visit per year	-2.62248
Nauru	2015	Gap 10 visit per year	-0.00577

For peer review only

Nepal	2019	Gap 10 visit per year	-31.37716
Netherlands	2018	Gap 10 visit per year	-8.77567
New Zealand	2018	Gap 10 visit per year	-1.01433
Nicaragua	2018	Gap 10 visit per year	-2.97474
Niger	2016	Gap 10 visit per year	-18.25563
Nigeria	2018	Gap 10 visit per year	-152.98801
Niue	2008	Gap 10 visit per year	-0.00144
North Korea	2017	Gap 10 visit per year	6.69613
Norway	2019	Gap 10 visit per year	1.71748
Oman	2019	Gap 10 visit per year	-3.32224
Pakistan	2019	Gap 10 visit per year	-177.99404
Palau	2014	Gap 10 visit per year	-0.03199
Panama	2019	Gap 10 visit per year	-3.40027
Papua New Guinea	2019	Gap 10 visit per year	-8.35706
Paraguay	2018	Gap 10 visit per year	-10.97663
Peru	2018	Gap 10 visit per year	-17.54471
Philippines	2017	Gap 10 visit per year	-106.23144
Poland	2017	Gap 10 visit per year	-82.88943
Portugal	2018	Gap 10 visit per year	-0.4933
Qatar	2018	Gap 10 visit per year	-1.24199
Romania	2017	Gap 10 visit per year	-36.61607
Russian Federation	2018	Gap 10 visit per year	-113.2309
Rwanda	2019	Gap 10 visit per year	-10.27869
Saint Kitts and Nevis	2018	Gap 10 visit per year	-0.07345
Saint Lucia	2017	Gap 10 visit per year	-0.32038
Saint Vincent and the Grenadines	2012	Gap 10 visit per year	-0.16168
Samoa	2016	Gap 10 visit per year	-0.23228
Sao Tome and Principe	2015	Gap 10 visit per year	-0.22626
Saudi Arabia	2018	Gap 10 visit per year	-6.42058
Senegal	2019	Gap 10 visit per year	-16.06996
Serbia	2016	Gap 10 visit per year	-14.46273
Seychelles	2018	Gap 10 visit per year	-0.12858
Sierra Leone	2018	Gap 10 visit per year	-8.48329
Singapore	2016	Gap 10 visit per year	-5.74674
Slovakia	2018	Gap 10 visit per year	-6.71347
Slovenia	2018	Gap 10 visit per year	-3.60569
Solomon Islands	2016	Gap 10 visit per year	-0.43898
Somalia	2014	Gap 10 visit per year	-15.11195
South Africa	2019	Gap 10 visit per year	-82.26635
South Korea	2018	Gap 10 visit per year	-44.81212
Spain	2018	Gap 10 visit per year	-3.22309
Sri Lanka	2019	Gap 10 visit per year	-31.3469
Sudan	2017	Gap 10 visit per year	-41.33897
Suriname	2018	Gap 10 visit per year	-0.96275
Swaziland	2016	Gap 10 visit per year	-1.34454

For peer review only

Sweden	2017	Gap 10 visit per year	-1.3279
Switzerland	2018	Gap 10 visit per year	3.36064
Syrian Arab Republic	2016	Gap 10 visit per year	-12.76653
Tajikistan	2014	Gap 10 visit per year	-5.09354
Tanzania	2014	Gap 10 visit per year	-42.55171
Thailand	2019	Gap 10 visit per year	-107.86784
The Gambia	2019	Gap 10 visit per year	-2.00849
Timor-Leste	2019	Gap 10 visit per year	-0.95793
Togo	2019	Gap 10 visit per year	-8.10237
Tonga	2013	Gap 10 visit per year	-0.11527
Trinidad and Tobago	2019	Gap 10 visit per year	-0.41505
Tunisia	2017	Gap 10 visit per year	-12.98208
Turkey	2018	Gap 10 visit per year	-66.02607
Turkmenistan	2014	Gap 10 visit per year	-1.04455
Tuvalu	2014	Gap 10 visit per year	-0.0171
Uganda	2017	Gap 10 visit per year	-24.09732
Ukraine	2014	Gap 10 visit per year	-75.83366
United Arab Emirates	2018	Gap 10 visit per year	-9.40385
United Kingdom	2019	Gap 10 visit per year	70.58424
United States of America	2018	Gap 10 visit per year	-261.36597
Uruguay	2017	Gap 10 visit per year	0.06188
Uzbekistan	2014	Gap 10 visit per year	-12.21877
Vanuatu	2016	Gap 10 visit per year	-0.31721
Venezuela	2017	Gap 10 visit per year	-28.00327
Viet Nam	2016	Gap 10 visit per year	-91.68196
Yemen	2014	Gap 10 visit per year	-13.46532
Zambia	2016	Gap 10 visit per year	-11.96245
Zimbabwe	2018	Gap 10 visit per year	-15.81287
Afghanistan	2016	Gap 11 visit per year	-29.097028
Albania	2019	Gap 11 visit per year	-5.602225
Algeria	2018	Gap 11 visit per year	-34.53217
Andorra	2015	Gap 11 visit per year	-0.060641
Angola	2017	Gap 11 visit per year	-25.617602
Antigua and Barbuda	2017	Gap 11 visit per year	-0.093951
Argentina	2017	Gap 11 visit per year	-28.102946
Armenia	2017	Gap 11 visit per year	-2.684564
Australia	2018	Gap 11 visit per year	-4.343237
Austria	2018	Gap 11 visit per year	-1.619802
Azerbaijan	2014	Gap 11 visit per year	-3.905373
Bahamas	2017	Gap 11 visit per year	-0.569081
Bahrain	2015	Gap 11 visit per year	-2.26295
Bangladesh	2019	Gap 11 visit per year	-156.157332
Barbados	2017	Gap 11 visit per year	-0.545098
Belarus	2015	Gap 11 visit per year	-10.332097
Belgium	2019	Gap 11 visit per year	7.131456

For peer review only - http://bmjopen.bmj.com/site/about/guidelines.xhtml

For peer review only

Belize	2018	Gap 11 visit per year	-0.397477
Benin	2019	Gap 11 visit per year	-9.992226
Bhutan	2019	Gap 11 visit per year	-1.303736
Bolivia	2017	Gap 11 visit per year	-7.793698
Bosnia and Herzegovina	2015	Gap 11 visit per year	-8.363943
Botswana	2018	Gap 11 visit per year	-3.902972
Brazil	2019	Gap 11 visit per year	-320.557697
Brunei Darussalam	2017	Gap 11 visit per year	-0.650986
Bulgaria	2018	Gap 11 visit per year	-11.602448
Burkina Faso	2019	Gap 11 visit per year	-17.495575
Burundi	2017	Gap 11 visit per year	-9.551182
Cabo Verde	2015	Gap 11 visit per year	-0.750471
Cambodia	2014	Gap 11 visit per year	-12.666608
Cameroon	2011	Gap 11 visit per year	-20.651606
Canada	2019	Gap 11 visit per year	-21.569712
Central African Republic	2015	Gap 11 visit per year	-5.725497
Chad	2019	Gap 11 visit per year	-13.750119
Chile	2018	Gap 11 visit per year	6.885792
China	2017	Gap 11 visit per year	-1385.703785
Colombia	2018	Gap 11 visit per year	16.183767
Comoros	2012	Gap 11 visit per year	-0.746956
Congo	2016	Gap 11 visit per year	-6.446177
Cook Islands	2014	Gap 11 visit per year	-0.032853
Costa Rica	2018	Gap 11 visit per year	-2.764549
Cote d'Ivoire	2019	Gap 11 visit per year	-28.299504
Croatia	2016	Gap 11 visit per year	-10.232561
Cuba	2018	Gap 11 visit per year	14.109355
Cyprus	2016	Gap 11 visit per year	-1.685577
Czech Republic	2019	Gap 11 visit per year	-14.432734
DR Congo	2013	Gap 11 visit per year	-65.19208
Denmark	2018	Gap 11 visit per year	-4.331218
Djibouti	2014	Gap 11 visit per year	-1.247594
Dominica	2018	Gap 11 visit per year	-0.154694
Dominican Republic	2019	Gap 11 visit per year	-17.688255
Ecuador	2017	Gap 11 visit per year	-1.788636
Egypt	2019	Gap 11 visit per year	-118.032289
El Salvador	2018	Gap 11 visit per year	-0.245433
Equatorial Guinea	2017	Gap 11 visit per year	-0.958513
Eritrea	2004	Gap 11 visit per year	-3.356026
Estonia	2017	Gap 11 visit per year	-1.742028
Ethiopia	2018	Gap 11 visit per year	-74.357988
Fiji	2015	Gap 11 visit per year	-1.197443
Finland	2018	Gap 11 visit per year	-3.639585
France	2018	Gap 11 visit per year	56.445561
Gabon	2017	Gap 11 visit per year	-1.539351

For peer review only

Georgia	2019	Gap 11 visit per year	2.697283
Germany	2018	Gap 11 visit per year	-44.742181
Ghana	2019	Gap 11 visit per year	-35.338986
Greece	2018	Gap 11 visit per year	5.110024
Grenada	2018	Gap 11 visit per year	-0.190688
Guatemala	2018	Gap 11 visit per year	-16.183951
Guinea	2016	Gap 11 visit per year	-12.750396
Guinea Bissau	2016	Gap 11 visit per year	-1.737459
Guyana	2018	Gap 11 visit per year	-0.746426
Haiti	2018	Gap 11 visit per year	-18.429658
Honduras	2017	Gap 11 visit per year	-9.344191
Hungary	2018	Gap 11 visit per year	-20.876774
Iceland	2019	Gap 11 visit per year	0.003269
India	2019	Gap 11 visit per year	-1407.248328
Indonesia	2019	Gap 11 visit per year	-484.96646
Iran	2018	Gap 11 visit per year	-47.075689
Iraq	2018	Gap 11 visit per year	-55.700422
Ireland	2019	Gap 11 visit per year	-2.814503
Israel	2019	Gap 11 visit per year	7.706078
Italy	2019	Gap 11 visit per year	60.619789
Jamaica	2016	Gap 11 visit per year	-5.859569
Japan	2018	Gap 11 visit per year	-208.344319
Jordan	2017	Gap 11 visit per year	-3.373437
Kazakhstan	2014	Gap 11 visit per year	-3.061478
Kenya	2018	Gap 11 visit per year	-44.637258
Kiribati	2013	Gap 11 visit per year	-0.138831
Kuwait	2015	Gap 11 visit per year	-2.242673
Kyrgyzstan	2014	Gap 11 visit per year	-3.093243
Lao PDR	2017	Gap 11 visit per year	-6.118709
Latvia	2018	Gap 11 visit per year	-3.575945
Lebanon	2018	Gap 11 visit per year	-2.781388
Lesotho	2010	Gap 11 visit per year	-2.71906
Liberia	2015	Gap 11 visit per year	-5.304482
Libya	2017	Gap 11 visit per year	-6.428915
Lithuania	2019	Gap 11 visit per year	-3.506032
Luxembourg	2017	Gap 11 visit per year	-0.482316
Macedonia (TFYR)	2015	Gap 11 visit per year	-3.858295
Madagascar	2014	Gap 11 visit per year	-24.466198
Malawi	2018	Gap 11 visit per year	-13.31044
Malaysia	2015	Gap 11 visit per year	-34.454593
Maldives	2018	Gap 11 visit per year	-0.247825
Mali	2018	Gap 11 visit per year	-16.474926
Malta	2015	Gap 11 visit per year	-0.501637
Marshall Islands	2012	Gap 11 visit per year	-0.053714
Mauritania	2018	Gap 11 visit per year	-4.136537

For peer review only - http://bmjopen.bmj.com/site/about/guidelines.xhtml

For peer review only

Mauritius	2018	Gap 11 visit per year	-1.403885
Mexico	2018	Gap 11 visit per year	105.122679
Micronesia (Federated States of)	2009	Gap 11 visit per year	-0.105808
Moldova	2019	Gap 11 visit per year	-7.513316
Mongolia	2018	Gap 11 visit per year	-0.555726
Montenegro	2018	Gap 11 visit per year	-1.16813
Morocco	2017	Gap 11 visit per year	-50.132054
Mozambique	2019	Gap 11 visit per year	-27.806172
Myanmar	2019	Gap 11 visit per year	-82.463593
Namibia	2017	Gap 11 visit per year	-2.955778
Nauru	2015	Gap 11 visit per year	-0.007047
Nepal	2019	Gap 11 visit per year	-35.672176
Netherlands	2018	Gap 11 visit per year	-12.814887
New Zealand	2018	Gap 11 visit per year	-1.926963
Nicaragua	2018	Gap 11 visit per year	-3.810314
Niger	2016	Gap 11 visit per year	-20.126193
Nigeria	2018	Gap 11 visit per year	-172.013961
Niue	2008	Gap 11 visit per year	-0.001734
North Korea	2017	Gap 11 visit per year	2.682393
Norway	2019	Gap 11 visit per year	0.575428
Oman	2019	Gap 11 visit per year	-4.134564
Pakistan	2019	Gap 11 visit per year	-207.898394
Palau	2014	Gap 11 visit per year	-0.036439
Panama	2019	Gap 11 visit per year	-4.086897
Papua New Guinea	2019	Gap 11 visit per year	-9.221766
Paraguay	2018	Gap 11 visit per year	-12.545343
Peru	2018	Gap 11 visit per year	-20.610731
Philippines	2017	Gap 11 visit per year	-120.011634
Poland	2017	Gap 11 visit per year	-95.692573
Portugal	2018	Gap 11 visit per year	-3.26653
Qatar	2018	Gap 11 visit per year	-1.711839
Romania	2017	Gap 11 visit per year	-43.206827
Russian Federation	2018	Gap 11 visit per year	-156.90364
Rwanda	2019	Gap 11 visit per year	-11.381159
Saint Kitts and Nevis	2018	Gap 11 visit per year	-0.088045
Saint Lucia	2017	Gap 11 visit per year	-0.358218
Saint Vincent and the Grenadines	2012	Gap 11 visit per year	-0.182948
Samoa	2016	Gap 11 visit per year	-0.258858
Sao Tome and Principe	2015	Gap 11 visit per year	-0.252036
Saudi Arabia	2018	Gap 11 visit per year	-11.463788
Senegal	2019	Gap 11 visit per year	-17.748706
Serbia	2016	Gap 11 visit per year	-17.287153
Seychelles	2018	Gap 11 visit per year	-0.153438
Sierra Leone	2018	Gap 11 visit per year	-9.359919
Singapore	2016	Gap 11 visit per year	-6.969764

For peer review only

Slovakia	2018	Gap 11 visit per year	-8.343717
Slovenia	2018	Gap 11 visit per year	-4.295809
Solomon Islands	2016	Gap 11 visit per year	-0.488878
Somalia	2014	Gap 11 visit per year	-16.638595
South Africa	2019	Gap 11 visit per year	-92.812635
South Korea	2018	Gap 11 visit per year	-55.454832
Spain	2018	Gap 11 visit per year	-12.953699
Sri Lanka	2019	Gap 11 visit per year	-35.71184
Sudan	2017	Gap 11 visit per year	-46.007017
Suriname	2018	Gap 11 visit per year	-1.082625
Swaziland	2016	Gap 11 visit per year	-1.484344
Sweden	2017	Gap 11 visit per year	-3.60559
Switzerland	2018	Gap 11 visit per year	1.849704
Syrian Arab Republic	2016	Gap 11 visit per year	-15.167433
Tajikistan	2014	Gap 11 visit per year	-6.313844
Tanzania	2014	Gap 11 visit per year	-46.957631
Thailand	2019	Gap 11 visit per year	-121.853324
The Gambia	2019	Gap 11 visit per year	-2.221939
Timor-Leste	2019	Gap 11 visit per year	-1.103423
Togo	2019	Gap 11 visit per year	-8.943957
Tonga	2013	Gap 11 visit per year	-0.129547
Trinidad and Tobago	2019	Gap 11 visit per year	-0.768755
Tunisia	2017	Gap 11 visit per year	-15.024888
Turkey	2018	Gap 11 visit per year	-80.066227
Turkmenistan	2014	Gap 11 visit per year	-1.757055
Tuvalu	2014	Gap 11 visit per year	-0.01931
Uganda	2017	Gap 11 visit per year	-26.852952
Ukraine	2014	Gap 11 visit per year	-90.166326
United Arab Emirates	2018	Gap 11 visit per year	-11.561485
United Kingdom	2019	Gap 11 visit per year	57.980314
United States of America	2018	Gap 11 visit per year	-330.084617
Uruguay	2017	Gap 11 visit per year	-0.780732
Uzbekistan	2014	Gap 11 visit per year	-17.052497
Vanuatu	2016	Gap 11 visit per year	-0.351231
Venezuela	2017	Gap 11 visit per year	-33.346897
Viet Nam	2016	Gap 11 visit per year	-104.727106
Yemen	2014	Gap 11 visit per year	-15.489852
Zambia	2016	Gap 11 visit per year	-13.234395
Zimbabwe	2018	Gap 11 visit per year	-17.545457
Afghanistan	2016	Gap 12 visit per year	-32.189576
Albania	2019	Gap 12 visit per year	-6.3272
Algeria	2018	Gap 12 visit per year	-40.97164
Andorra	2015	Gap 12 visit per year	-0.077972
Angola	2017	Gap 12 visit per year	-28.237384
Antigua and Barbuda	2017	Gap 12 visit per year	-0.114492

For peer review only - http://bmjopen.bmj.com/site/about/guidelines.xhtml

For peer review only

Argentina	2017	Gap 12 visit per year	-38.626532
Armenia	2017	Gap 12 visit per year	-3.517888
Australia	2018	Gap 12 visit per year	-8.992804
Austria	2018	Gap 12 visit per year	-3.873284
Azerbaijan	2014	Gap 12 visit per year	-5.749316
Bahamas	2017	Gap 12 visit per year	-0.654452
Bahrain	2015	Gap 12 visit per year	-2.5264
Bangladesh	2019	Gap 12 visit per year	-175.072044
Barbados	2017	Gap 12 visit per year	-0.627016
Belarus	2015	Gap 12 visit per year	-13.498424
Belgium	2019	Gap 12 visit per year	4.655952
Belize	2018	Gap 12 visit per year	-0.452384
Benin	2019	Gap 12 visit per year	-10.935292
Bhutan	2019	Gap 12 visit per year	-1.438212
Bolivia	2017	Gap 12 visit per year	-9.026216
Bosnia and Herzegovina	2015	Gap 12 visit per year	-9.461256
Botswana	2018	Gap 12 visit per year	-4.287424
Brazil	2019	Gap 12 visit per year	-371.865624
Brunei Darussalam	2017	Gap 12 visit per year	-0.741212
Bulgaria	2018	Gap 12 visit per year	-14.005716
Burkina Faso	2019	Gap 12 visit per year	-19.1729
Burundi	2017	Gap 12 visit per year	-10.468744
Cabo Verde	2015	Gap 12 visit per year	-0.837332
Cambodia	2014	Gap 12 visit per year	-13.951936
Cameroon	2011	Gap 12 visit per year	-22.612752
Canada	2019	Gap 12 visit per year	-27.684004
Central African Republic	2015	Gap 12 visit per year	-6.260724
Chad	2019	Gap 12 visit per year	-15.039448
Chile	2018	Gap 12 visit per year	3.099864
China	2017	Gap 12 visit per year	-1640.26772
Colombia	2018	Gap 12 visit per year	8.977564
Comoros	2012	Gap 12 visit per year	-0.820452
Congo	2016	Gap 12 visit per year	-7.056284
Cook Islands	2014	Gap 12 visit per year	-0.036976
Costa Rica	2018	Gap 12 visit per year	-3.673508
Cote d'Ivoire	2019	Gap 12 visit per year	-31.061868
Croatia	2016	Gap 12 visit per year	-11.736612
Cuba	2018	Gap 12 visit per year	11.05266
Cyprus	2016	Gap 12 visit per year	-1.942584
Czech Republic	2019	Gap 12 visit per year	-17.744528
DR Congo	2013	Gap 12 visit per year	-71.41036
Denmark	2018	Gap 12 visit per year	-5.829556
Djibouti	2014	Gap 12 visit per year	-1.370148
Dominica	2018	Gap 12 visit per year	-0.172348
Dominican Republic	2019	Gap 12 visit per year	-20.00496

For peer review only

Ecuador	2017	Gap 12 visit per year	-3.645012
Egypt	2019	Gap 12 visit per year	-132.168088
El Salvador	2018	Gap 12 visit per year	-1.105336
Equatorial Guinea	2017	Gap 12 visit per year	-1.068696
Eritrea	2004	Gap 12 visit per year	-3.670892
Estonia	2017	Gap 12 visit per year	-2.108076
Ethiopia	2018	Gap 12 visit per year	-81.499396
Fiji	2015	Gap 12 visit per year	-1.340256
Finland	2018	Gap 12 visit per year	-5.13532
France	2018	Gap 12 visit per year	42.273612
Gabon	2017	Gap 12 visit per year	-1.743292
Georgia	2019	Gap 12 visit per year	1.656536
Germany	2018	Gap 12 visit per year	-65.055152
Ghana	2019	Gap 12 visit per year	-38.698712
Greece	2018	Gap 12 visit per year	2.596708
Grenada	2018	Gap 12 visit per year	-0.215296
Guatemala	2018	Gap 12 visit per year	-17.933492
Guinea	2016	Gap 12 visit per year	-13.953932
Guinea Bissau	2016	Gap 12 visit per year	-1.905728
Guyana	2018	Gap 12 visit per year	-0.878692
Haiti	2018	Gap 12 visit per year	-20.223536
Honduras	2017	Gap 12 visit per year	-10.326072
Hungary	2018	Gap 12 visit per year	-24.278208
Iceland	2019	Gap 12 visit per year	-0.060252
India	2019	Gap 12 visit per year	-1592.824176
Indonesia	2019	Gap 12 visit per year	-534.77532
Iran	2018	Gap 12 visit per year	-57.246388
Iraq	2018	Gap 12 visit per year	-62.000824
Ireland	2019	Gap 12 visit per year	-3.814276
Israel	2019	Gap 12 visit per year	6.289676
Italy	2019	Gap 12 visit per year	44.076588
Jamaica	2016	Gap 12 visit per year	-6.452348
Japan	2018	Gap 12 visit per year	-241.621348
Jordan	2017	Gap 12 visit per year	-4.713704
Kazakhstan	2014	Gap 12 visit per year	-6.469976
Kenya	2018	Gap 12 visit per year	-49.060736
Kiribati	2013	Gap 12 visit per year	-0.152452
Kuwait	2015	Gap 12 visit per year	-2.907916
Kyrgyzstan	2014	Gap 12 visit per year	-3.962356
Lao PDR	2017	Gap 12 visit per year	-6.792728
Latvia	2018	Gap 12 visit per year	-4.19044
Lebanon	2018	Gap 12 visit per year	-3.690196
Lesotho	2010	Gap 12 visit per year	-2.97252
Liberia	2015	Gap 12 visit per year	-5.794344
Libya	2017	Gap 12 visit per year	-7.63868

For peer review only - http://bmjopen.bmj.com/site/about/guidelines.xhtml

For peer review only

Lithuania	2019	Gap 12 visit per year	-4.456944
Luxembourg	2017	Gap 12 visit per year	-0.607072
Macedonia (TFYR)	2015	Gap 12 visit per year	-4.48064
Madagascar	2014	Gap 12 visit per year	-26.884716
Malawi	2018	Gap 12 visit per year	-14.54998
Malaysia	2015	Gap 12 visit per year	-39.700056
Maldives	2018	Gap 12 visit per year	-0.3104
Mali	2018	Gap 12 visit per year	-18.084192
Malta	2015	Gap 12 visit per year	-0.603604
Marshall Islands	2012	Gap 12 visit per year	-0.059688
Mauritania	2018	Gap 12 visit per year	-4.549904
Mauritius	2018	Gap 12 visit per year	-1.67742
Mexico	2018	Gap 12 visit per year	86.862468
Micronesia (Federated States of)	2009	Gap 12 visit per year	-0.116336
Moldova	2019	Gap 12 visit per year	-8.666572
Mongolia	2018	Gap 12 visit per year	-1.161292
Montenegro	2018	Gap 12 visit per year	-1.35296
Morocco	2017	Gap 12 visit per year	-55.871468
Mozambique	2019	Gap 12 visit per year	-30.450824
Myanmar	2019	Gap 12 visit per year	-91.770556
Namibia	2017	Gap 12 visit per year	-3.289076
Nauru	2015	Gap 12 visit per year	-0.008324
Nepal	2019	Gap 12 visit per year	-39.967192
Netherlands	2018	Gap 12 visit per year	-16.854104
New Zealand	2018	Gap 12 visit per year	-2.839596
Nicaragua	2018	Gap 12 visit per year	-4.645888
Niger	2016	Gap 12 visit per year	-21.996756
Nigeria	2018	Gap 12 visit per year	-191.039912
Niue	2008	Gap 12 visit per year	-0.002028
North Korea	2017	Gap 12 visit per year	-1.331344
Norway	2019	Gap 12 visit per year	-0.566624
Oman	2019	Gap 12 visit per year	-4.946888
Pakistan	2019	Gap 12 visit per year	-237.802748
Palau	2014	Gap 12 visit per year	-0.040888
Panama	2019	Gap 12 visit per year	-4.773524
Papua New Guinea	2019	Gap 12 visit per year	-10.086472
Paraguay	2018	Gap 12 visit per year	-14.114056
Peru	2018	Gap 12 visit per year	-23.676752
Philippines	2017	Gap 12 visit per year	-133.791828
Poland	2017	Gap 12 visit per year	-108.495716
Portugal	2018	Gap 12 visit per year	-6.03976
Qatar	2018	Gap 12 visit per year	-2.181688
Romania	2017	Gap 12 visit per year	-49.797584
Russian Federation	2018	Gap 12 visit per year	-200.57638
Rwanda	2019	Gap 12 visit per year	-12.483628

For peer review only

Saint Kitts and Nevis	2018	Gap 12 visit per year	-0.10264
Saint Lucia	2017	Gap 12 visit per year	-0.396056
Saint Vincent and the Grenadines	2012	Gap 12 visit per year	-0.204216
Samoa	2016	Gap 12 visit per year	-0.285436
Sao Tome and Principe	2015	Gap 12 visit per year	-0.277812
Saudi Arabia	2018	Gap 12 visit per year	-16.506996
Senegal	2019	Gap 12 visit per year	-19.427452
Serbia	2016	Gap 12 visit per year	-20.111576
Seychelles	2018	Gap 12 visit per year	-0.178296
Sierra Leone	2018	Gap 12 visit per year	-10.236548
Singapore	2016	Gap 12 visit per year	-8.192788
Slovakia	2018	Gap 12 visit per year	-9.973964
Slovenia	2018	Gap 12 visit per year	-4.985928
Solomon Islands	2016	Gap 12 visit per year	-0.538776
Somalia	2014	Gap 12 visit per year	-18.16524
South Africa	2019	Gap 12 visit per year	-103.35892
South Korea	2018	Gap 12 visit per year	-66.097544
Spain	2018	Gap 12 visit per year	-22.684308
Sri Lanka	2019	Gap 12 visit per year	-40.07678
Sudan	2017	Gap 12 visit per year	-50.675064
Suriname	2018	Gap 12 visit per year	-1.2025
Swaziland	2016	Gap 12 visit per year	-1.624148
Sweden	2017	Gap 12 visit per year	-5.88328
Switzerland	2018	Gap 12 visit per year	0.338768
Syrian Arab Republic	2016	Gap 12 visit per year	-17.568336
Tajikistan	2014	Gap 12 visit per year	-7.534148
Tanzania	2014	Gap 12 visit per year	-51.363552
Thailand	2019	Gap 12 visit per year	-135.838808
The Gambia	2019	Gap 12 visit per year	-2.435388
Timor-Leste	2019	Gap 12 visit per year	-1.248916
Togo	2019	Gap 12 visit per year	-9.785544
Tonga	2013	Gap 12 visit per year	-0.143824
Trinidad and Tobago	2019	Gap 12 visit per year	-1.12246
Tunisia	2017	Gap 12 visit per year	-17.067696
Turkey	2018	Gap 12 visit per year	-94.106384
Turkmenistan	2014	Gap 12 visit per year	-2.46956
Tuvalu	2014	Gap 12 visit per year	-0.02152
Uganda	2017	Gap 12 visit per year	-29.608584
Ukraine	2014	Gap 12 visit per year	-104.498992
United Arab Emirates	2018	Gap 12 visit per year	-13.71912
United Kingdom	2019	Gap 12 visit per year	45.376388
United States of America	2018	Gap 12 visit per year	-398.803264
Uruguay	2017	Gap 12 visit per year	-1.623344
Uzbekistan	2014	Gap 12 visit per year	-21.886224
Vanuatu	2016	Gap 12 visit per year	-0.385252

For peer review only

Venezuela	2017	Gap 12 visit per year	-38.690524
Viet Nam	2016	Gap 12 visit per year	-117.772252
Yemen	2014	Gap 12 visit per year	-17.514384
Zambia	2016	Gap 12 visit per year	-14.50634
Zimbabwe	2018	Gap 12 visit per year	-19.278044

A negative gap would mean that the *demand* exceeded the *offer*. In other words, the healthcare system would not have enough physicians to meet the needs of medical appointments per year for all patients with hypertension.

For peer review only

Estimating the gap between demand and supply of medical appointments by physicians for hypertension care: A pooled analysis in 191 countries

Journal:	*BMJ Open*
Manuscript ID	bmjopen-2021-059933.R1
Article Type:	Original research
Date Submitted by the Author:	16-Feb-2022
Complete List of Authors:	Carrillo Larco, Rodrigo M. ; Imperial College London,<br>Guzman-Vilca, Wilmer Cristobal; CRONICAS Centre of Excellence in Chronic Diseases<br>Neupane, Dinesh; Johns Hopkins University
**Primary Subject Heading**:	Epidemiology
Secondary Subject Heading:	Epidemiology
Keywords:	Epidemiology < TROPICAL MEDICINE, Public health < INFECTIOUS DISEASES, Hypertension < CARDIOLOGY

SCHOLARONE™
Manuscripts

I, the Submitting Author has the right to grant and does grant on behalf of all authors of the Work (as defined in the below author licence), an exclusive licence and/or a non-exclusive licence for contributions from authors who are: i) UK Crown employees; ii) where BMJ has agreed a CC-BY licence shall apply, and/or iii) in accordance with the terms applicable for US Federal Government officers or employees acting as part of their official duties; on a worldwide, perpetual, irrevocable, royalty-free basis to BMJ Publishing Group Ltd ("BMJ") its licensees and where the relevant Journal is co-owned by BMJ to the co-owners of the Journal, to publish the Work in this journal and any other BMJ products and to exploit all rights, as set out in our licence.

The Submitting Author accepts and understands that any supply made under these terms is made by BMJ to the Submitting Author unless you are acting as an employee on behalf of your employer or a postgraduate student of an affiliated institution which is paying any applicable article publishing charge ("APC") for Open Access articles. Where the Submitting Author wishes to make the Work available on an Open Access basis (and intends to pay the relevant APC), the terms of reuse of such Open Access shall be governed by a Creative Commons licence – details of these licences and which Creative Commons licence will apply to this Work are set out in our licence referred to above.

Other than as permitted in any relevant BMJ Author's Self Archiving Policies, I confirm this Work has not been accepted for publication elsewhere, is not being considered for publication elsewhere and does not duplicate material already published. I confirm all authors consent to publication of this Work and authorise the granting of this licence.

For peer review only

Estimating the gap between demand and supply of medical appointments by physicians for hypertension care: A pooled analysis in 191 countries

Rodrigo M. Carrillo-Larco[1,2,3]

Wilmer Cristobal Guzman-Vilca[2,4,5]

Dinesh Neupane[6]

1. Department of Epidemiology and Biostatistics, School of Public Health, Imperial College London, London, UK.
2. CRONICAS Centre of Excellence in Chronic Diseases, Universidad Peruana Cayetano Heredia, Lima, Peru.
3. Universidad Continental, Lima, Peru.
4. School of Medicine "Alberto Hurtado", Universidad Peruana Cayetano Heredia, Lima, Peru.
5. Sociedad Científica de Estudiantes de Medicina Cayetano Heredia (SOCEMCH), Universidad Peruana Cayetano Heredia, Lima, Peru.
6. Department of Epidemiology, Welch Center for Prevention, Epidemiology and Clinical Research, Johns Hopkins University, Baltimore, MD, USA.

Corresponding Author

Rodrigo M Carrillo-Larco, MD

Department of Epidemiology and Biostatistics, School of Public Health, Imperial College London, London, UK.

rcarrill@ic.ac.uk

For peer review only

ABSTRACT

Introduction: With a growing number of people with hypertension, the limited number of physicians could not provide treatment to all patients. We quantified the gap between medical appointments available and needed for hypertension care, overall and in relation to hypertension treatment cascade metrics.

Methods: Ecological descriptive analysis. We combined country-year-specific data on hypertension prevalence, awareness, treatment and control (from NCD-RisC) and number of physicians (from WHO). We estimated from 1 to 12 medical appointments per year for hypertensive patients. We assumed that physicians could see 25 patients per day, work 200 days per year, and dedicate 10% of their time to hypertension care.

Results: We studied 191 countries. Forty-one countries would not have enough physicians to provide at least 1 medical appointment per year to all the population with hypertension; these countries were low/lower-middle income and in Sub-Saharan Africa or East Asia and Pacific. Regardless of the world region, ≥50% of countries would not have enough physicians to provide ≥8 medical appointments to their population with hypertension. Countries where the demand exceeded the offer of medical appointments for hypertension care had worse hypertension diagnosis, treatment and control rates than countries where the demand did not exceed the offer. There were positive correlations between the physician density and hypertension diagnosis ($r=0.70$, $p<0.001$), treatment ($r=0.70$, $p<0.001$) and control ($r=0.59$, $p<0.001$).

Conclusions: Where physicians are the only healthcare professionals allowed to prescribe antihypertensive medications, particularly in low- and middle-income countries, the healthcare system may struggle to deliver antihypertensive treatment to hypertensive patients.

Key words: non-communicable diseases; population health; health metrics; health systems; hypertension.

For peer review only

Strengths and limitations

- We built on previous research which proposed a solid and reasonable methodology to quantify the gap between the *offer* and *demand* of medical appointments for hypertension care at the country level.
- All of our results are not for the same year. Potential country-specific users of our results are advised to understand our estimates taking into consideration the data year.
- Because of hypertension data availability, we restricted our analysis to the age group 30-79 years.
- Third, we used the number of physicians as reported by the World Health Organization, and assumed each physician sees 25 patients per day and works 200 days per year. We are not aware of empirical data to support this (or other) scenario.
- Fourth, country-level estimates often hide within country differences.

For peer review only

INTRODUCTION

Globally, high blood pressure is the leading risk factor for cardiovascular morbidity and mortality.[1] Even though there are effective treatments for hypertension,[2] these do not always reach the patients leading to low hypertension treatment and control rates.[3, 4] Reasons for poor access to antihypertensive treatment could include lack of medicines, inability to afford treatment, and not having health insurance. However, where universal health coverage is available, the obstacle to provide treatment for patients with hypertension could be the shortage of physicians,[5] particularly where physicians are the only health professionals authorized to prescribe medicines for hypertension. This may be the case in many countries, though there is no empirical data to characterize the actual organization of hypertension care in all countries. Due to the lifelong nature of antihypertensive treatment, people with hypertension will need multiple medical appointments per year whereby physicians will titrate and refill their medicines. While the number of people with hypertension grows faster than the number of physicians, and hypertension diagnosis rates increases,[4] countries will struggle to provide the medical appointments needed for patients with hypertension; that is, there will be more medical appointments needed than available for hypertension care.

The gap between the number of medical appointments needed and available for people with hypertension has been quantified showing that several countries do not have enough physicians for three, six or 12 medical appointments per year for people with hypertension.[6] Nonetheless, previous estimates were up to 2015 and the authors did not study the gap in relation to hypertension care cascade metrics: diagnosis, treatment and control. Using more recent data will provide countries with up-to-date evidence to inform their policies, interventions, and targets. Also, the correlations between the gap and hypertension care cascade metrics will inform if countries with larger gaps also have worse hypertension treatment and control rates.

Consequently, elaborating on previous evidence and overcoming the above described limitations,[6] we quantified the gap between the number of medical appointments needed by people with hypertension and the number of medical appointments available for hypertension care based on the latest available data.

For peer review only - http://bmjopen.bmj.com/site/about/guidelines.xhtml

METHODS

This is an ecological descriptive analysis. We combined data from different sources to quantify the hypertension gap regarding *demand* of medical appointments by people with hypertension and the availability of physicians to provide medical appointments for hypertension care (i.e., *offer*). In addition, we presented the gap estimates in relation to hypertension treatment cascade metrics: diagnosed, treated and controlled. Further details about the data sources and analysis are shown in Supplementary File 1.

Data sources

The prevalence of hypertension in each country amongst people aged 30-79 years was retrieved from the Non-Communicable Disease Risk Factor Collaboration (NCD-RisC);[4, 7] the data are publically available online.[7] From this data source we also extracted the proportion diagnosed, treated and controlled amongst people with hypertension (i.e., hypertension care cascade).[4] The NCD-RisC estimates are sex-stratified. In here, we reported one estimate per country (i.e., men and women combined). For this, we multiplied the sex-specific prevalence by the number of men and women, and summed these products to have the country prevalence.

The number of physicians in each country was retrieved from the World Health Organization.[8] Population data (number of people per country) were retrieved from the Institute for Health Metrics and Evaluation (IHME).[9] The world region and income group for each country was retrieved from the World Bank.[10]

The hypertension data (source: NCD-RisC[4, 7]) is available for all years until 2019, whereas the number of physicians for each country is available at different years (i.e., some countries have more recent data than others). We combined the number of physicians with the year-corresponding hypertension data (e.g., number of physician data in 2017 with hypertension data in 2017); population data was for the same year. We only used the most recent World Bank region and income classification (regardless of the physician or hypertension data year).[10]

Variables

In here, *demand* refers to the number of medical appointments needed by people with hypertension. As in a previous paper,[6] we assumed three scenarios: three, six and 12 medical appointments by year. We multiplied the absolute number of people with hypertension times

For peer review only - http://bmjopen.bmj.com/site/about/guidelines.xhtml

three, six and 12 to compute the number of medical appointments needed. In addition to these three scenarios, we considered one through 12 medical appointments per year; that is, if people with hypertension would need from one to 12 medical appointments per year.

In here, *offer* refers to the response of the health system to the appointments needed (*demand*) by people with hypertension. We assumed that a physician could see 25 patients per day and he/she works 200 days per year (25 x 200 = 5,000); we multiplied the number of physicians times 5,000 to compute the number of medical appointments available. This product was further multiplied by 0.10 because we assumed that 10% of a physician's time is dedicated to hypertension care.[6]

In here, *the gap* refers to the difference between the *offer* and the *demand*. That is, we subtracted the absolute number of medical appointments needed (*demand*) from the absolute number of medical appointments available (*offer*), for the three scenarios (three, six and 12 medical appointments) and from one through 12 medical appointments per year. These subtractions were further divided by 1,000,000 so that the *gap* is reported per 1,000,000 medical appointments. A negative gap suggests that the *demand* exceeded the *offer*; that is, there were more medical appointments needed than available for hypertension care.[6]

Statistical analysis

Results are presented as counts (absolute numbers), as proportions or percentages, as well as medians. Heatmaps and line plots are used to show at what number of medical appointments needed the gap turned negative. We used world maps to show countries with a negative gap according to the three scenarios (three, six and 12 medical appointments). We used boxplots to depict the mean of the hypertension care cascade metrics (diagnosed, treated and controlled) by the three scenarios amongst countries with positive and negative gaps. We used scatter plots to correlate the physician density (absolute number of physicians per 1,000 people) and the hypertension care cascade metrics; these plots had the Pearson correlation coefficients.

Ethics

We used data available in the public domain and at the country level. The data did not contain any personal identifiers. We did not seek approval by an ethics committee. The opinions herein

For peer review only - http://bmjopen.bmj.com/site/about/guidelines.xhtml

expressed are those of the authors alone. The funder had no role in the study design, analysis, results interpretation or conclusions. All authors approved the submitted version.

Patient and Public Involvement

It was not appropriate or possible to involve patients or the public in the design, or conduct, or reporting, or dissemination plans of our research

RESULTS

Data characteristics

There were 191 countries: 30 in East Asia and Pacific, 49 in Europe and Central Asia, 33 in Latin America and the Caribbean, 20 in Middle East and North Africa, 2 in North America, 8 in South Asia and 47 in Sub-Saharan Africa; of note, two countries were not classified into any of these groups: Cook Islands and Nauru. Fifty-eight countries were high income, 54 were upper-middle-income, 49 were lower-middle income, and 28 were low income; of note, two countries (Cook Islands and Nauru) were not classified into any of these groups. Data regarding the number of physicians were available from 2004 to 2019, with 85% available since 2015.

Underlying variables

The prevalence of hypertension ranged from 20% to 56%; the median across all countries was 37%; the median by region was 42% in Europe and Central Asia, 40% in Latin America and the Caribbean, 36% in South Asia, 35% in East Asia and Pacific, 34% in Sub-Saharan Africa, 33% in Middle East and North Africa, and 32% in North America.

The density of physicians (per 1,000 people) ranged from 0.07 to 13.77 (median = 3.26); the median physician density (per 1,000 people) according to the region was 0.36 in Sub-Saharan Africa, 1.75 in East Asia and Pacific, 1.99 in South Asia, 3.67 in Middle East and North Africa, 3.84 in Latin America and the Caribbean, 4.34 in North America, and 6.09 in Europe and Central Asia.

Gap: available (*offer*) medical appointments minus demand

Out of 191 countries in the analysis, 41 countries would have a negative gap to provide one medical appointment per year for people with hypertension (Figure 1); that is, in 41 countries, and

For peer review only - http://bmjopen.bmj.com/site/about/guidelines.xhtml

under the assumptions of our analysis, the healthcare system would not have enough physicians for all patients with hypertension to have at least one medical appointment per year.

According to their income, these 41 countries were either low income (21 countries) or lower-middle income (19 countries); one country (Botswana) was upper-middle income. According to their location, these 41 countries were in Sub-Saharan Africa (35 countries; largest gap in DR Congo), East Asia and Pacific (4 countries; largest gap in Papua New Guinea), Middle East and North Africa (1 country; Djibouti) and Latin America and the Caribbean (1 country; Haiti).

The number of countries with a negative gap (more *demand* than *offer*) increased with the number of medical appointments per year (Figure 2). Africa had the largest proportion of countries with a negative gap regardless of the number of medical appointments. Regardless of the world region, ≥50% of the countries could not offer eight or more medical appointments per year to the population with hypertension.

Gap: available (*offer*) medical appointments minus demand in fixed scenarios

For a fixed number of three medical appointments (i.e., every four months) per year, 80 (42%) countries would have a negative gap (more *demand* than *offer*); 24 of these countries were low income whereas two were high income (Figure 3). For a fixed number of six medical appointments (i.e., twice per year) per year, 132 (69%) countries would have a negative gap; 27 of these countries were low income whilst 23 were high income. For a fixed number of 12 medical appointments (i.e., monthly) per year, 179 (94%) countries would have a negative gap; 28 of these countries were low income while 50 were high income.

Gap: available medical appointments minus demand in fixed scenarios and in relation to hypertension diagnosis, treatment, and control

After stratifying the number of visits according to the three scenarios (three, six and 12 medical appointments per year), those countries with a negative gap (more *demand* than *offer*) had worse hypertension treatment cascade metrics than those countries without a negative gap (Figure 4). For example, across countries with always a negative gap, the median prevalence of diagnosed hypertension was 40% versus a median prevalence of 63% across countries without a negative gap; the corresponding estimates for hypertension treatment were 23% versus 52%, and for hypertension control were 9% versus 23%. That is, in countries where the *demand* exceeded the

For peer review only - http://bmjopen.bmj.com/site/about/guidelines.xhtml

offer, the hypertension treatment cascade metrics were worse (on average) than in countries where the *demand* did not exceed the *offer* of medical appointments for hypertension care per year.

Correlation between hypertension diagnosis, treatment and control with physician density

For all three hypertension care cascade metrics, there was a positive correlation with the physician density (Figure 5). The magnitude of the correlation was stronger for hypertension diagnosis and treatment than for hypertension control. Low income countries were most often found in the bottom left quadrant, with worse hypertension care cascade metrics and lower physician density. Conversely, high income countries were often found in the upper right quadrant, with better hypertension care cascade metrics and higher physician density.

DISCUSSION

Leveraging on high-quality and official data sources[7, 9, 10] and building on previous research,[6] we documented that 41 countries would not have enough physicians to provide at least one medical appointment per year to their population with hypertension; these countries were either low or lower-middle income and located in Sub-Saharan Africa or in East Asia and Pacific. Regardless of the world region, more than half of all countries would not have enough physicians to provide eight or more medical appointments to their population with hypertension per year. On average, countries where the *demand* exceeded the *offer* of medical appointments for people with hypertension also had worse hypertension care cascade metrics than those countries where the *demand* did not exceed the *offer*. There were positive correlations between the density of physicians and hypertension diagnosis, treatment and control rates.

The health consequences of these gaps would include fewer opportunities to access medical appointments for hypertension control, hence higher likelihood to not reach optimal blood pressure levels through pharmacological treatment. This is partially supported by the ecological correlations we reported, whereby higher physician density was positively correlated with higher rates of hypertension treatment and control. Further from not achieving hypertension control, this could lead to higher morbidity, mortality and disability due to cardiovascular diseases.

We followed the methodology developed by the authors of the only global work on this subject.[6] Despite minor changes in our methodology (e.g., updated data sources) which led to different

For peer review only - http://bmjopen.bmj.com/site/about/guidelines.xhtml

numeric results, we reached similar conclusions:[6] many low- and middle-income countries would struggle to provide medical appointments per year to their population with hypertension. This does not mean that the more medical appoints for hypertension care the better; in other words, quantity does not necessarily reflect quality. We advocate for innovative approaches whereby people with hypertension receive the best evidence-based care available, so that they receive pharmacological and non-pharmacological care to delay or avoid complications.

Vedanthan *et al.* reported a positive association between physician density and hypertension treatment rates in the unadjusted model only;[5] potential explanations for the lack of statistical significance in adjusted model include data collation year and not having estimates for some countries where there could have been a large gap. We computed correlation coefficients showing a positive correlation between physician density and hypertension treatment; arguably, this shows a similar result to their unadjusted estimates.[5] However, we advanced the literature by showing the correlation between physician density and hypertension diagnosis and control.

We built on previous research which proposed a solid and reasonable methodology to quantify the gap between the *offer* and *demand* of medical appointments for hypertension care at the country level.[6] This work substantially advanced our previous publication by using the most recent hypertension data which is comparable across all countries;[4] by focusing on hypertension (rather than raised blood pressure[6]), which accounts for aware and unaware cases; and by studying the gap between *offer* and *demand* in relation to hypertension care cascade metrics.

Notwithstanding, there are limitations we need to acknowledge. First, all of our results are not for the same year. Potential country-specific users of our results are advised to understand our estimates taking into consideration the data year (Supplementary File 2). Health organizations should also make efforts to secure consistent, comparable and recent data about the number of physicians in all countries and when possible, stratified by medical speciality. Second, because of hypertension data availability, we restricted our analysis to the age group 30-79 years.[4] Perhaps, older people may require more medical appointments because of multimorbidity and polypharmacy. If so, our results show a conservative scenario and alert that the gap between *offer* and *demand* of medical appointments for hypertension care could be larger if we had included the entire adult population. Third, we used the number of physicians as reported by the

For peer review only - http://bmjopen.bmj.com/site/about/guidelines.xhtml

World Health Organization, and assumed each physician sees 25 patients per day and works 200 days per year.[6] Although conservative and possible, we are not aware of empirical data to support this (or other) scenario. Any other scenario, either to replace our assumption or as a sensitivity analysis, would also be an educated assumption because of the lack of empirical evidence to inform the scenarios. International health organizations, through surveys or country consultations, could collect data on the actual load of the healthcare workforce and how much time is allocated to key tasks (e.g., non-communicable diseases care). Fourth, country-level estimates often hide within country differences. Our results should be interpreted in light of this limitation, and we encourage researchers and health officials to conduct similar analysis at the sub-national level to tailor local policies and interventions. Fifth, the correlations between the gap and the hypertension care cascade metrics were at the country level. This ecological analysis should be cautiously interpreted and need further verification with individual-level data. Sixth, we conducted a descriptive work to characterize the gap between medical appointments by physicians needed and available for people with hypertension worldwide. A quantification of the health consequences of this gap was beyond the scope of this work, though it is undoubtedly a relevant question that deserves attention. Finally, the most salient limitation of our work is that we assumed only physicians prescribe treatment for hypertension yet other healthcare personnel may participate in this task. Unfortunately, there are no global data to make the distinction on whether all hypertension care is provided by physicians or other personnel. In other words, there is no global data (e.g., encyclopaedia) characterizing the actual organization of hypertension care in all countries. This descriptive work aimed to quantify the gap at the country level, and providing an in-depth profile of the organization of hypertension care in 191 countries is beyond the scope of this work or any available global data. This work alerts of a large gap to meet the needs of people with hypertension, though the gap may be smaller if other healthcare personnel provide treatment for people with hypertension.

The fact that 41 countries would not have enough physicians to provide one medical appointment per year to their population with hypertension is alarming; similarly, having three medical appoints per year (i.e., one every four months) was also unfeasible in many countries.

Task shifting strategies[11, 12] could offer a potential solution for countries where the *demand* exceeded the *offer* of medical appointments for hypertension care. This could include training

For peer review only - http://bmjopen.bmj.com/site/about/guidelines.xhtml

For peer review only

nurses and other non-physician health professionals,[13-16] as well as community health workers or champions of the community,[16] to monitor blood pressure and to dispense antihypertensive treatment. Physicians could oversee these strategies and focus on difficult cases, such as patients with resistant hypertension or when treatment requires careful tailoring. Having antihypertensive treatment refilled in the local pharmacy could also help.[16, 17] Future research should quantify the impact of task shifting strategies to improve care for people with hypertension.

Countries could adopt prescription strategies that meet their resources. For example, if a country could only have four medical appointments per year for people with hypertension, they could provide medicines for three months. Patients would have enough pills until the next appointment. This would require careful consideration by the physician to identify patients who would not need frequent medical appointments, according to the patient's overall health profile.

There is strong evidence showing the value of non-physician health professionals to improve hypertension care.[11, 12, 16, 17] Selecting the best examples which could be implemented following rigorous research methods and evaluations deserves prompt attention.

The COVID-19 pandemic would impact our results and the associated implications. First, the latest physician data was for 2019. Unfortunately, many physicians have recently lost their lives to COVID-19,[18] potentially widening the herein documented gaps especially in low- and middle-income countries. Second, COVID-19 disrupted health services because they stopped their regular activities to take care of COVID-19 patients; lockdowns also disrupted regular health services.[19] Therefore, people with chronic diseases including hypertension had their usual care interrupted. This could have led to treatment discontinuation and fewer patients meeting blood pressure targets. As health services resume their regular activities, patients with chronic conditions may need more than usual care to catch up with their treatments. This would translate into more frequent medical appointments widening the gap herein documented.

Brain drain of physicians could also play a relevant role.[20] Physicians from low- and middle-income countries, where we found the largest gaps, tend to migrate to high-income countries. If this continues, the herein document gaps in low- and middle-income countries would increase.

Both of these potential drivers could be solved with more physicians, though this may not be a realistic option. To support timely and continues treatment for people with hypertension where

For peer review only - http://bmjopen.bmj.com/site/about/guidelines.xhtml

physicians are the only health professional authorized to prescribe medicines, it seems imperative to find solutions that does not require physicians only.

Many low and middle-income countries would not meet the *demand* of medical appointments needed by people with hypertension. This would lead to lower rates of hypertension treatment and control at the country level. Because hypertension is the leading risk factor for cardiovascular morbidity and mortality,[1] and has poor treatment and control rates in many low- and middle-income countries,[4] healthcare systems should secure better care for people with hypertension including the uninterrupted provision of antihypertensive medication. A synergic participation of physicians, non-physician healthcare workers and lay people (e.g., community health workers) would be needed to secure continuous treatment for (all) people with hypertension.

For peer review only - http://bmjopen.bmj.com/site/about/guidelines.xhtml

Acknowledgements

None.

Funding

RMC-L is supported by a Wellcome Trust International Training Fellowship (Wellcome Trust 214185/Z/18/Z). DN receives support from Resolve to Save Lives, an initiative of Vital Strategies, which is funded by Bloomberg Philanthropies, the Bill & Melinda Gates Foundation, and Gates Philanthropy Partners, which is funded with support from the Chan Zuckerberg Initiative.

Conflict of interest

None to declare.

Contributions

RMC-L, WCG-V and DN conceived the study. RMC-L, WCG-V and DN discussed the planning and execution of the study. WCGV collated, pooled and conducted the analysis. RMC-L conducted the analysis and drafted the first version of the manuscript. RMC-L, WCG-V and DN discussed the findings and provided critical input to the manuscript. RMC-L, WCG-V and DN approved the submitted version. RMC-L submitted the work for publication. RMC-L, WCG-V and DN addressed the comments by the external peer reviewers.

Data availability

All data sources herein analysed are in the public domain, and links to these sources have been provided in the main manuscript. We are also providing the analysis data (i.e., after pooling individual data sources) as Supplementary File 3.

For peer review only

FIGURES

Figure 1. Indicator of positive or negative gap by number of medical appointments (1 through 12) per year by country.

A negative gap indicates that there is more demand (need of medical appointment by people with hypertension) than offer (number of physicians).

Figure 2. Proportion of countries with a negative gap by number of medical appointments (1 through 12) per year by world region.

Two countries (Cook Islands and Nauru) were not included in the plot because their region was not available in the data source (World Bank).

Figure 3. World maps showing countries according to the gap level by the three possible scenarios, and signalling which countries would always have a negative gap regardless of the scenario.

Figure 4. Hypertension treatment cascade metrics by positive or negative gap stratified by the three possible scenarios.

Each dot represents a country.

Figure 5. Correlation between hypertension treatment cascade metrics and physician density.

Each dot is a country; colours indicate the income group of each country.

For peer review only

REFERENCES

1. GBD 2019 Risk Factors Collaborators. Global burden of 87 risk factors in 204 countries and territories, 1990-2019: a systematic analysis for the Global Burden of Disease Study 2019. *Lancet (London, England)* 2020; **396**(10258): 1223-49.

2. Zhou B, Perel P, Mensah GA, Ezzati M. Global epidemiology, health burden and effective interventions for elevated blood pressure and hypertension. *Nature reviews Cardiology* 2021: 1-18.

3. Attaei MW, Khatib R, McKee M, et al. Availability and affordability of blood pressure-lowering medicines and the effect on blood pressure control in high-income, middle-income, and low-income countries: an analysis of the PURE study data. *The Lancet Public health* 2017; **2**(9): e411-e9.

4. NCD Risk Factor Collaboration (NCD-RisC). Worldwide trends in hypertension prevalence and progress in treatment and control from 1990 to 2019: a pooled analysis of 1201 population-representative studies with 104 million participants. *Lancet (London, England)* 2021; **398**(10304): 957-80.

5. Vedanthan R, Ray M, Fuster V, Magenheim E. Hypertension Treatment Rates and Health Care Worker Density. *Hypertension (Dallas, Tex : 1979)* 2019; **73**(3): 594-601.

6. Neupane D, Gao Y, Feng Y, Matsushita K, Appel LJ. Estimation of the Global Gap in Clinic Visits for Hypertension Care Between Patient Need and Physician Capacity. *Hypertension (Dallas, Tex : 1979)* 2021; **78**(3): 779-86.

7. Non-Communicable Diseases Risk Factor Collaboration (NCD-RisC). [cited 2021 Sept 23]. Available from: https://ncdrisc.org/.

8. The Global Health Observatory - Medical doctors (number). [cited 2021 Sep 23]. Available from: https://www.who.int/data/gho/data/indicators/indicator-details/GHO/medical-doctors-(number).

9. Global Burden of Disease Study 2019 (GBD 2019) Population Estimates 1950-2019. [cited 2021 Sep 23]. Available from: http://ghdx.healthdata.org/record/ihme-data/gbd-2019-population-estimates-1950-2019.

For peer review only - http://bmjopen.bmj.com/site/about/guidelines.xhtml

10. World Bank Country and Leading Groups. [cited 2021 Sep 23]. Available from: https://datahelpdesk.worldbank.org/knowledgebase/articles/906519-world-bank-country-and-lending-groups.

11. Joshi R, Alim M, Kengne AP, et al. Task shifting for non-communicable disease management in low and middle income countries--a systematic review. *PloS one* 2014; **9**(8): e103754.

12. Ogedegbe G, Gyamfi J, Plange-Rhule J, et al. Task shifting interventions for cardiovascular risk reduction in low-income and middle-income countries: a systematic review of randomised controlled trials. *BMJ open* 2014; **4**(10): e005983.

13. Kavita, Thakur JS, Vijayvergiya R, Ghai S. Task shifting of cardiovascular risk assessment and communication by nurses for primary and secondary prevention of cardiovascular diseases in a tertiary health care setting of Northern India. *BMC health services research* 2020; **20**(1): 10.

14. Ogedegbe G, Plange-Rhule J, Gyamfi J, et al. Health insurance coverage with or without a nurse-led task shifting strategy for hypertension control: A pragmatic cluster randomized trial in Ghana. *PLoS medicine* 2018; **15**(5): e1002561.

15. Schwalm JD, McCready T, Lopez-Jaramillo P, et al. A community-based comprehensive intervention to reduce cardiovascular risk in hypertension (HOPE 4): a cluster-randomised controlled trial. *Lancet (London, England)* 2019; **394**(10205): 1231-42.

16. Anand TN, Joseph LM, Geetha AV, Prabhakaran D, Jeemon P. Task sharing with non-physician health-care workers for management of blood pressure in low-income and middle-income countries: a systematic review and meta-analysis. *The Lancet Global health* 2019; **7**(6): e761-e71.

17. Wagner TD, Jones MC, Salgado TM, Dixon DL. Pharmacist's role in hypertension management: a review of key randomized controlled trials. *Journal of human hypertension* 2020; **34**(7): 487-94.

18. Gouda D, Singh PM, Gouda P, Goudra B. An Overview of Health Care Worker Reported Deaths During the COVID-19 Pandemic. *Journal of the American Board of Family Medicine : JABFM* 2021; **34**(Suppl): S244-s6.

For peer review only

For peer review only - http://bmjopen.bmj.com/site/about/guidelines.xhtml

19. Skeete J, Connell K, Ordunez P, DiPette DJ. Approaches to the Management of Hypertension in Resource-Limited Settings: Strategies to Overcome the Hypertension Crisis in the Post-COVID Era. *Integrated blood pressure control* 2020; **13**: 125-33.

20. Botezat A, Ramos R. Physicians' brain drain - a gravity model of migration flows. *Globalization and Health* 2020; **16**(1): 7.

For peer review only

For peer review only - http://bmjopen.bmj.com/site/about/guidelines.xhtml

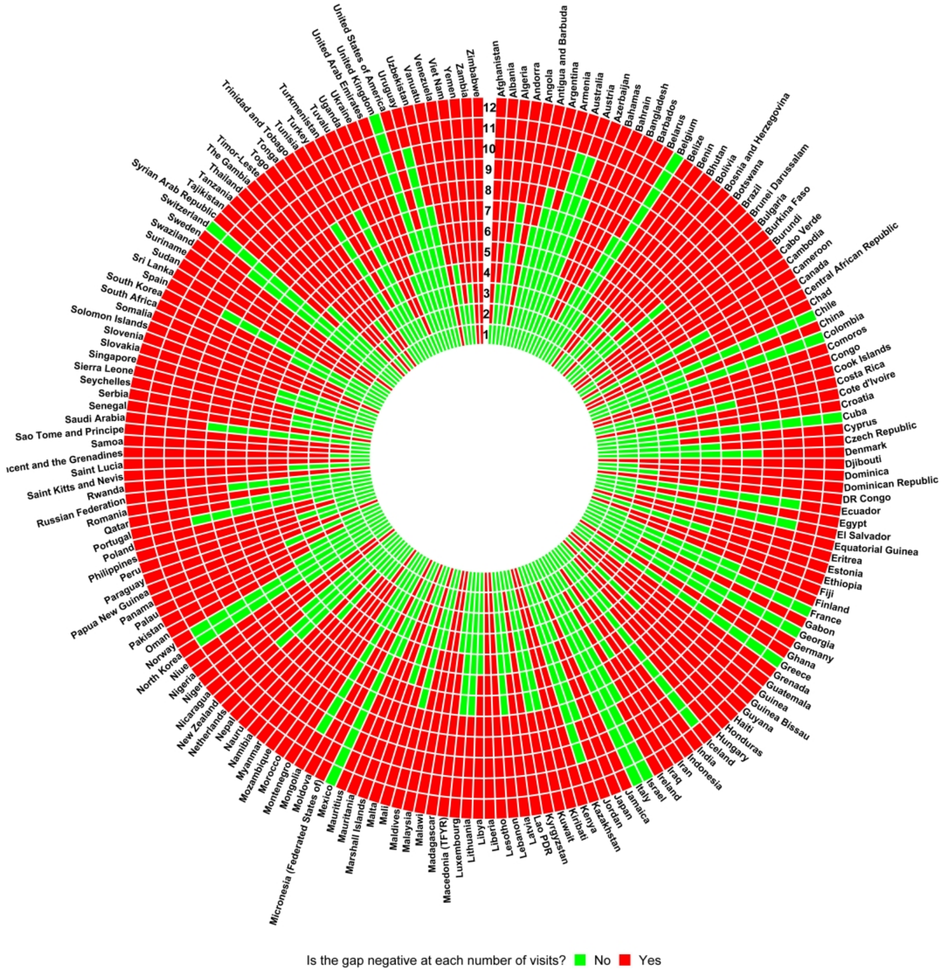

Figure 1. Indicator of positive or negative gap by number of medical appointments (1 through 12) per year by country.
A negative gap indicates that there is more demand (need of medical appointment by people with hypertension) than offer (number of physicians).

1033x1033mm (118 x 118 DPI)

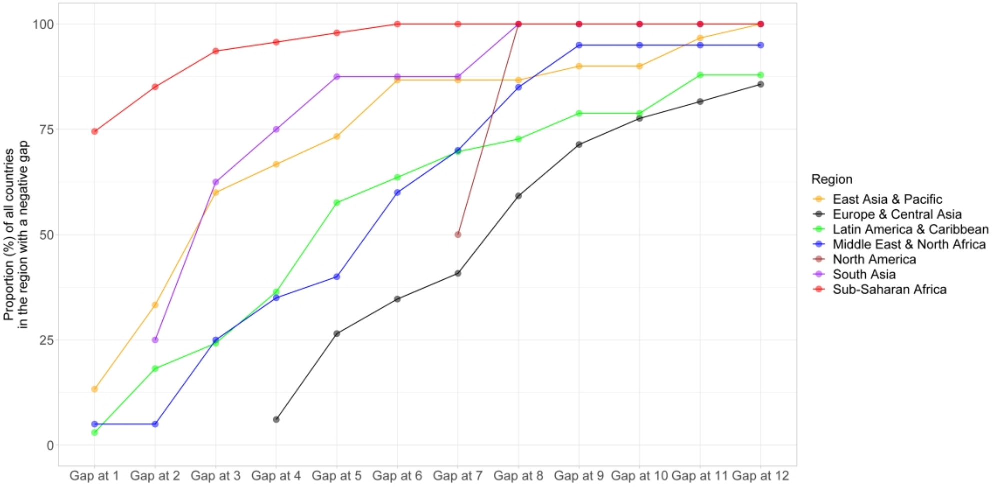

Figure 2. Proportion of countries with a negative gap by number of medical appointments (1 through 12) per year by world region.
Two countries (Cook Islands and Nauru) were not included in the plot because their region was not available in the data source (World Bank).

1033x516mm (118 x 118 DPI)

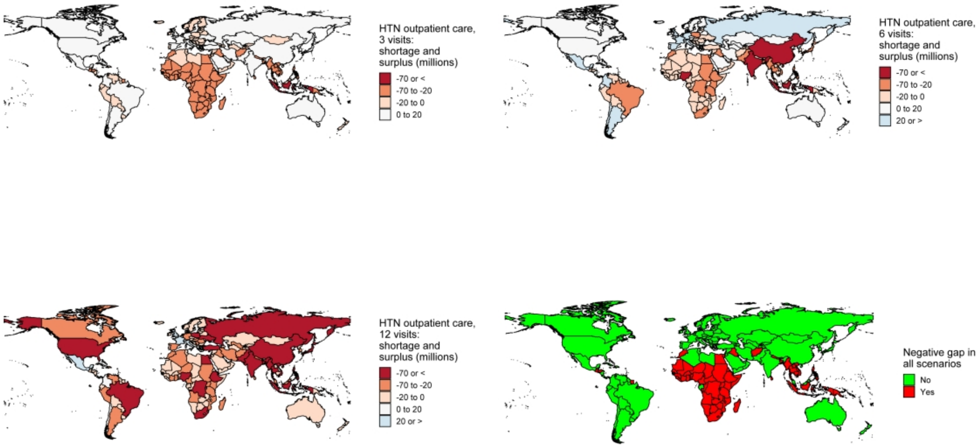

Figure 3. World maps showing countries according to the gap level by the three possible scenarios, and signalling which countries would always have a negative gap regardless of the scenario.

1033x619mm (118 x 118 DPI)

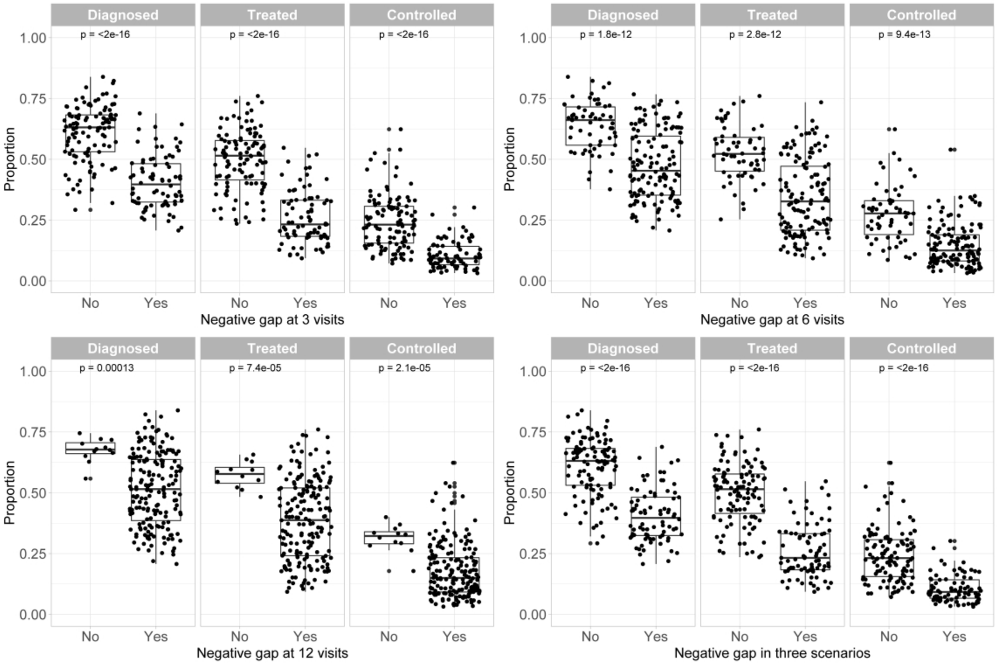

Figure 4. Hypertension treatment cascade metrics by positive or negative gap stratified by the three possible scenarios.
Each dot represents a country.

1033x688mm (118 x 118 DPI)

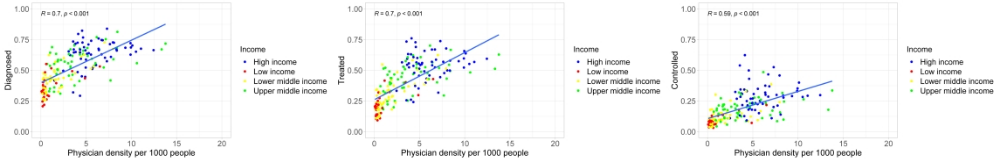

Figure 5. Correlation between hypertension treatment cascade metrics and physician density. Each dot is a country; colours indicate the income group of each country.

1033x165mm (118 x 118 DPI)

SUPPLEMENTARY MATERIAL

Estimating the gap between demand and supply of medical appointments by physicians for hypertension care: A pooled analysis in 191 countries

Corresponding Author

Rodrigo M Carrillo-Larco, MD

Department of Epidemiology and Biostatistics, School of Public Health, Imperial College London, London, UK.

r.carrillo-larco@imperial.ac.uk

For peer review only

For peer review only - http://bmjopen.bmj.com/site/about/guidelines.xhtml

EXPANDED METHODS 3

Data sources 3

Data management 4

New variables 4

References 6

For peer review only

For peer review only - http://bmjopen.bmj.com/site/about/guidelines.xhtml

EXPANDED METHODS

All analysis codes are available as Supplementary Materials.

Data sources

We combined multiple data sources.

- Hypertension[1, 2] data from the Non-Communicable Disease Risk Factor Collaboration (NCD-RisC). These data included prevalence of hypertension, and amongst people with hypertension, these data also provided the proportion of diagnosed (i.e., awareness), treated and controlled. These data are for all countries and for all years until 2019. All these hypertension estimates are sex-stratified and for the age group 30-79 years.
- The number of physicians[3] was retrieved from the World Health Organization (WHO) observatory. These data are for all countries, though the latest available data may be different by country (i.e., not all countries have data for the same years). The most recent data was for the year 2019 (not all countries have data until 2019).

Year	Number of countries with data	Proportion of all data
2004	1	0.005
2008	1	0.005
2009	1	0.005
2010	1	0.005
2011	1	0.005
2012	3	0.016
2013	3	0.016
2014	16	0.084
2015	14	0.073
2016	17	0.089
2017	32	0.168
2018	60	0.314
2019	41	0.215

For peer review only - http://bmjopen.bmj.com/site/about/guidelines.xhtml

- Population[4] data (number of inhabitants per country) was retrieved from the Institute for Health Metrics and Evaluation (IHME). We extracted the number of men and women in the age group 30-79 for all years until 2019.
- The World Bank classification[5] was used to label countries according to income (low, middle and high income) and world region. We only used the latest available data.

Data management

We downloaded each data source. In the dataset for number of physicians we only kept the most recent year per country. The hypertension data and the number of physicians data were merged matching by year and country; in other words, both data sources belonged to the same year. These data were then merged with population data matching by year, country and sex (because hypertension data were sex-stratified). Matching by country, we then merged with the World Bank data; the World Bank data would not necessarily be synchronic with the other data. Overall, hypertension, number of physicians and population data was synchronic (belonged to the same year).

New variables

The sex-specific prevalence of hypertension was multiplied by the number of men and women in each country, to compute the absolute number of men and women with hypertension. These sex-specific absolute numbers of people with hypertension were then multiplied by the proportion of diagnosed, treated and controlled; this, to compute the absolute number of people with diagnosed, treated and controlled hypertension. Because all these estimates were sex-specific, we summed them within each country to have one estimate per country (i.e., overall by country rather than sex-stratified). For diagnosis, treatment and control rates, we followed a similar procedure to move from sex-stratified estimates to one-only per country.

We computed the number of medical appointments needed by people with hypertension, hereafter referred to as *demand*. We multiplied the absolute number of people with hypertension by [1…6…12]. If people with hypertension would need 1 medical appointment per year, then there would be as many medical appointment as people with hypertension (multiplication by 1). On the other hand, if people with hypertension would need 12 medical appointments per year, then there

For peer review only - http://bmjopen.bmj.com/site/about/guidelines.xhtml

would be as many medical appointments as 12 times the number of people with hypertension (multiplication by 12). The same logic would apply for all natural numbers between 1 and 12.

We computed the number of medical appointments available for people with hypertension, hereafter referred to as *offer*. For this, we multiplied the number of physicians by 5,000; this factor was conceived assuming that a physician could see 25 patients per day and work 200 days per year (25 x 200 = 5,000). The product between the number of physicians and 5,000 was then multiplied by 0.10, because we assumed that 10% of their time was dedicated to hypertension care.

We computed the gap between the *offer* and *demand* (*offer* - *demand*), as a simple subtraction between the number of medical appointments available (*offer*) and the number of medical appointments needed by people with hypertension (*demand*). We did this subtraction 12 times, for each of the potential number of medical appointments per year (1 through 12, as described in the second paragraph of this section above).

A negative gap would mean that the *demand* exceeded the *offer*. In other words, the healthcare system would not have enough physicians to meet the needs of medical appointments per year for all patients with hypertension.

For peer review only

For peer review only - http://bmjopen.bmj.com/site/about/guidelines.xhtml

References

1. Non-Communicable Diseases Risk Factor Collaboration (NCD-RisC). [cited 2021 Sept 23]. Available from: https://ncdrisc.org/.

2. NCD Risk Factor Collaboration (NCD-RisC). Worldwide trends in hypertension prevalence and progress in treatment and control from 1990 to 2019: a pooled analysis of 1201 population-representative studies with 104 million participants. *Lancet (London, England)* 2021; **398**(10304): 957-80.

3. The Global Health Observatory - Medical doctors (number). [cited 2021 Sep 23]. Available from: https://www.who.int/data/gho/data/indicators/indicator-details/GHO/medical-doctors-(number).

4. Global Burden of Disease Study 2019 (GBD 2019) Population Estimates 1950-2019. [cited 2021 Sep 23]. Available from: http://ghdx.healthdata.org/record/ihme-data/gbd-2019-population-estimates-1950-2019.

5. World Bank Country and Leading Groups. [cited 2021 Sep 23]. Available from: https://datahelpdesk.worldbank.org/knowledgebase/articles/906519-world-bank-country-and-lending-groups.

For peer review only

For peer review only

Supplementary Table 1. Gap (expressed in 1,000,000 medical appointments) between offer of medical appointments and demand of medical appointments by people with hypertension per year, from one through twelve medical appointments per year.
A negative gap would mean that the demand exceeded the offer. In other words, the healthcare system would not have enough physicians to meet the needs of medical appointments per year for all patients with hypertension.

Year	Country	Population	Population with hypertension	Medical appointments available	Medical appointments needed for 1 visit per year	Medical appointments needed for 2 visit per year	Medical appointments needed for 3 visit per year	Medical appointments needed for 4 visit per year	Medical appointments needed for 5 visit per year	Medical appointments needed for 6 visit per year	Medical appointments needed for 7 visit per year	Medical appointments needed for 8 visit per year	Medical appointments needed for 9 visit per year	Medical appointments needed for 10 visit per year	Medical appointments needed for 11 visit per year	Medical appointments needed for 12 visit per year	Gap 1 visit per year	Gap 2 visit per year	Gap 3 visit per year	Gap 4 visit per year	Gap 5 visit per year	Gap 6 visit per year	Gap 7 visit per year	Gap 8 visit per year	Gap 9 visit per year	Gap 10 visit per year	Gap 11 visit per year	Gap 12 visit per year
2016	Afghanistan	8613793	3092548	4921000	3092548	6185096	[illegible]	[illegible]	[illegible]	[illegible]	[illegible]	[illegible]	[illegible]	[illegible]	[illegible]	[illegible]	1.828452	-1.264096	-4.356644	-7.449192	-10.54174	-13.634288	-16.726836	-19.819384	-22.911932	-26.00448	-29.097028	-32.189576
2017	Angola	7613910	2619782	3200000	2619782	5239564	[illegible]	[illegible]	[illegible]	[illegible]	[illegible]	[illegible]	[illegible]	[illegible]	[illegible]	[illegible]	0.580218	-2.039564	-4.659346	-7.279128	-9.89891	-12.518692	-15.138474	-17.758256	-20.378038	-22.99782	-25.617602	-28.237384
2019	Albania	1539508	724975	2372500	724975	1449950	[illegible]	[illegible]	[illegible]	[illegible]	[illegible]	[illegible]	[illegible]	[illegible]	[illegible]	[illegible]	1.647525	0.92255	0.197575	-0.5274	-1.252375	-1.97735	-2.702325	-3.4273	-4.152275	-4.87725	-5.602225	-6.3272
2015	Andorra	51034	17331	130000	17331	34662	[illegible]	[illegible]	[illegible]	[illegible]	[illegible]	[illegible]	[illegible]	[illegible]	[illegible]	[illegible]	0.112669	0.095338	0.078007	0.060676	0.043345	0.026014	0.008683	-0.008648	-0.025979	-0.04331	-0.060641	-0.077972
2018	United Arab Emirates	6840833	2157635	12172500	2157635	4315270	[illegible]	[illegible]	[illegible]	[illegible]	[illegible]	[illegible]	[illegible]	[illegible]	[illegible]	[illegible]	10.014865	7.85723	5.699595	3.54196	1.384325	-0.77331	-2.930945	-5.08858	-7.246215	-9.40385	-11.561485	-13.71912
2017	Argentina	21888467	10523586	87656500	10523586	21047172	[illegible]	[illegible]	[illegible]	[illegible]	[illegible]	[illegible]	[illegible]	[illegible]	[illegible]	[illegible]	77.132914	66.609328	56.085742	45.562156	35.03857	24.514984	13.991398	3.467812	-7.055774	-17.57936	-28.102946	-38.626532
2017	Armenia	1686949	833324	6482000	833324	1666648	[illegible]	[illegible]	[illegible]	[illegible]	[illegible]	[illegible]	[illegible]	[illegible]	[illegible]	[illegible]	5.648676	4.815352	3.982028	3.148704	2.31538	1.482056	0.648732	-0.184592	-1.017916	-1.85124	-2.684564	-3.517888
2017	Antigua and Barbuda	47745	20541	132000	20541	41082	[illegible]	[illegible]	[illegible]	[illegible]	[illegible]	[illegible]	[illegible]	[illegible]	[illegible]	[illegible]	0.111459	0.090918	0.070377	0.049836	0.029295	0.008754	-0.011787	-0.032328	-0.052869	-0.07341	-0.093951	-0.114492
2018	Australia	13964789	4649567	46802000	4649567	9299134	[illegible]	[illegible]	[illegible]	[illegible]	[illegible]	[illegible]	[illegible]	[illegible]	[illegible]	[illegible]	42.152433	37.502866	32.853299	28.203732	23.554165	18.904598	14.255031	9.605464	4.955897	0.30633	-4.343237	-8.992804
2018	Austria	5526517	2253482	23168500	2253482	4506964	[illegible]	[illegible]	[illegible]	[illegible]	[illegible]	[illegible]	[illegible]	[illegible]	[illegible]	[illegible]	20.915018	18.661536	16.408054	14.154572	11.90109	9.647608	7.394126	5.140644	2.887162	0.63368	-1.619802	-3.873284
2014	Azerbaijan	4724667	1843943	16378000	1843943	3687886	[illegible]	[illegible]	[illegible]	[illegible]	[illegible]	[illegible]	[illegible]	[illegible]	[illegible]	[illegible]	14.534057	12.690114	10.846171	9.002228	7.158285	5.314342	3.470399	1.626456	-0.217487	-2.06143	-3.905373	-5.749316
2017	Burundi	3040275	917562	542000	917562	1835124	[illegible]	[illegible]	[illegible]	[illegible]	[illegible]	[illegible]	[illegible]	[illegible]	[illegible]	[illegible]	-0.375562	-1.293124	-2.210686	-3.128248	-4.04581	-4.963372	-5.880934	-6.798496	-7.716058	-8.63362	-9.551182	-10.468744
2019	Belgium	6803897	2475504	34362000	2475504	4951008	[illegible]	[illegible]	[illegible]	[illegible]	[illegible]	[illegible]	[illegible]	[illegible]	[illegible]	[illegible]	31.886496	29.410992	26.935488	24.459984	21.98448	19.508976	17.033472	14.557968	12.082464	9.60696	7.131456	4.655952
2019	Benin	3376730	943066	381500	943066	1886132	[illegible]	[illegible]	[illegible]	[illegible]	[illegible]	[illegible]	[illegible]	[illegible]	[illegible]	[illegible]	-0.561566	-1.504632	-2.447698	-3.390764	-4.33383	-5.276896	-6.219962	-7.163028	-8.106094	-9.04916	-9.992226	-10.935292
2019	Burkina Faso	6255571	1677325	955000	1677325	3354650	[illegible]	[illegible]	[illegible]	[illegible]	[illegible]	[illegible]	[illegible]	[illegible]	[illegible]	[illegible]	-0.722325	-2.39965	-4.076975	-5.7543	-7.431625	-9.10895	-10.786275	-12.4636	-14.140925	-15.81825	-17.495575	-19.1729
2019	Bangladesh	69461908	18914712	51904500	18914712	37829424	[illegible]	[illegible]	[illegible]	[illegible]	[illegible]	[illegible]	[illegible]	[illegible]	[illegible]	[illegible]	32.989788	14.075076	-4.839636	-23.754348	-42.66906	-61.583772	-80.498484	-99.413196	-118.327908	-137.24262	-156.157332	-175.072044
2018	Bulgaria	4615918	2403268	14833500	2403268	4806536	[illegible]	[illegible]	[illegible]	[illegible]	[illegible]	[illegible]	[illegible]	[illegible]	[illegible]	[illegible]	12.430232	10.026964	7.623696	5.220428	2.81716	0.413892	-1.989376	-4.392644	-6.795912	-9.19918	-11.602448	-14.005716
2015	Bahrain	849562	263450	635000	263450	526900	[illegible]	[illegible]	[illegible]	[illegible]	[illegible]	[illegible]	[illegible]	[illegible]	[illegible]	[illegible]	[illegible]	[illegible]	[illegible]	[illegible]	[illegible]	[illegible]	[illegible]	[illegible]	[illegible]	[illegible]	[illegible]	[illegible]
2017	Bahamas	192702	85371	370000	85371	170742	[illegible]	[illegible]	[illegible]	[illegible]	[illegible]	[illegible]	[illegible]	[illegible]	[illegible]	[illegible]	[illegible]	[illegible]	[illegible]	[illegible]	[illegible]	[illegible]	[illegible]	[illegible]	[illegible]	[illegible]	[illegible]	[illegible]
2015	Bosnia and Herzegovina	2180561	1097313	3706500	1097313	2194626	[illegible]	[illegible]	[illegible]	[illegible]	[illegible]	[illegible]	[illegible]	[illegible]	[illegible]	[illegible]	[illegible]	[illegible]	[illegible]	[illegible]	[illegible]	[illegible]	[illegible]	[illegible]	[illegible]	[illegible]	[illegible]	[illegible]
2015	Belarus	5841148	3166327	24497500	3166327	6332654	[illegible]	[illegible]	[illegible]	[illegible]	[illegible]	[illegible]	[illegible]	[illegible]	[illegible]	[illegible]	[illegible]	[illegible]	[illegible]	[illegible]	[illegible]	[illegible]	[illegible]	[illegible]	[illegible]	[illegible]	[illegible]	[illegible]
2018	Belize	157591	54907	206500	54907	109814	[illegible]	[illegible]	[illegible]	[illegible]	[illegible]	[illegible]	[illegible]	[illegible]	[illegible]	[illegible]	[illegible]	[illegible]	[illegible]	[illegible]	[illegible]	[illegible]	[illegible]	[illegible]	[illegible]	[illegible]	[illegible]	[illegible]
2017	Bolivia	4551849	1232518	5764000	[illegible]	[illegible]	[illegible]	[illegible]	[illegible]	[illegible]	[illegible]	[illegible]	[illegible]	[illegible]	[illegible]	[illegible]	[illegible]	[illegible]	[illegible]	[illegible]	[illegible]	[illegible]	[illegible]	[illegible]	[illegible]	[illegible]	[illegible]	[illegible]
2019	Brazil	113606668	51307927	243829500	[illegible]	[illegible]	[illegible]	[illegible]	[illegible]	[illegible]	[illegible]	[illegible]	[illegible]	[illegible]	[illegible]	[illegible]	[illegible]	[illegible]	[illegible]	[illegible]	[illegible]	[illegible]	[illegible]	[illegible]	[illegible]	[illegible]	[illegible]	[illegible]
2017	Barbados	175426	81918	356000	[illegible]	[illegible]	[illegible]	[illegible]	[illegible]	[illegible]	[illegible]	[illegible]	[illegible]	[illegible]	[illegible]	[illegible]	[illegible]	[illegible]	[illegible]	[illegible]	[illegible]	[illegible]	[illegible]	[illegible]	[illegible]	[illegible]	[illegible]	[illegible]
2017	Brunei Darussalam	211764	90226	341500	[illegible]	[illegible]	[illegible]	[illegible]	[illegible]	[illegible]	[illegible]	[illegible]	[illegible]	[illegible]	[illegible]	[illegible]	[illegible]	[illegible]	[illegible]	[illegible]	[illegible]	[illegible]	[illegible]	[illegible]	[illegible]	[illegible]	[illegible]	[illegible]
2019	Bhutan	329060	134476	175500	[illegible]	[illegible]	[illegible]	[illegible]	[illegible]	[illegible]	[illegible]	[illegible]	[illegible]	[illegible]	[illegible]	[illegible]	[illegible]	[illegible]	[illegible]	[illegible]	[illegible]	[illegible]	[illegible]	[illegible]	[illegible]	[illegible]	[illegible]	[illegible]
2018	Botswana	950840	384452	326000	[illegible]	[illegible]	[illegible]	[illegible]	[illegible]	[illegible]	[illegible]	[illegible]	[illegible]	[illegible]	[illegible]	[illegible]	[illegible]	[illegible]	[illegible]	[illegible]	[illegible]	[illegible]	[illegible]	[illegible]	[illegible]	[illegible]	[illegible]	[illegible]
2015	Central African Republic	1424395	535227	162000	[illegible]	[illegible]	[illegible]	[illegible]	[illegible]	[illegible]	[illegible]	[illegible]	[illegible]	[illegible]	[illegible]	[illegible]	[illegible]	[illegible]	[illegible]	[illegible]	[illegible]	[illegible]	[illegible]	[illegible]	[illegible]	[illegible]	[illegible]	[illegible]
2019	Canada	22169650	6114292	45687500	[illegible]	[illegible]	[illegible]	[illegible]	[illegible]	[illegible]	[illegible]	[illegible]	[illegible]	[illegible]	[illegible]	[illegible]	[illegible]	[illegible]	[illegible]	[illegible]	[illegible]	[illegible]	[illegible]	[illegible]	[illegible]	[illegible]	[illegible]	[illegible]
2018	Switzerland	5415243	1510936	18470000	[illegible]	[illegible]	[illegible]	[illegible]	[illegible]	[illegible]	[illegible]	[illegible]	[illegible]	[illegible]	[illegible]	[illegible]	[illegible]	[illegible]	[illegible]	[illegible]	[illegible]	[illegible]	[illegible]	[illegible]	[illegible]	[illegible]	[illegible]	[illegible]
2018	Chile	9801706	3785928	48531000	[illegible]	[illegible]	[illegible]	[illegible]	[illegible]	[illegible]	[illegible]	[illegible]	[illegible]	[illegible]	[illegible]	[illegible]	[illegible]	[illegible]	[illegible]	[illegible]	[illegible]	[illegible]	[illegible]	[illegible]	[illegible]	[illegible]	[illegible]	[illegible]
2017	China	867488855	254563935	1414499500	[illegible]	[illegible]	[illegible]	[illegible]	[illegible]	[illegible]	[illegible]	[illegible]	[illegible]	[illegible]	[illegible]	[illegible]	[illegible]	[illegible]	[illegible]	[illegible]	[illegible]	[illegible]	[illegible]	[illegible]	[illegible]	[illegible]	[illegible]	[illegible]
2019	Cote d'Ivoire	8267360	2762364	2086500	[illegible]	[illegible]	[illegible]	[illegible]	[illegible]	[illegible]	[illegible]	[illegible]	[illegible]	[illegible]	[illegible]	[illegible]	[illegible]	[illegible]	[illegible]	[illegible]	[illegible]	[illegible]	[illegible]	[illegible]	[illegible]	[illegible]	[illegible]	[illegible]
2011	Cameroon	6171752	1961146	921000	[illegible]	[illegible]	[illegible]	[illegible]	[illegible]	[illegible]	[illegible]	[illegible]	[illegible]	[illegible]	[illegible]	[illegible]	[illegible]	[illegible]	[illegible]	[illegible]	[illegible]	[illegible]	[illegible]	[illegible]	[illegible]	[illegible]	[illegible]	[illegible]
2013	DR Congo	20215043	6218280	3209000	[illegible]	[illegible]	[illegible]	[illegible]	[illegible]	[illegible]	[illegible]	[illegible]	[illegible]	[illegible]	[illegible]	[illegible]	[illegible]	[illegible]	[illegible]	[illegible]	[illegible]	[illegible]	[illegible]	[illegible]	[illegible]	[illegible]	[illegible]	[illegible]
2016	Congo	1718024	610107	265000	[illegible]	[illegible]	[illegible]	[illegible]	[illegible]	[illegible]	[illegible]	[illegible]	[illegible]	[illegible]	[illegible]	[illegible]	[illegible]	[illegible]	[illegible]	[illegible]	[illegible]	[illegible]	[illegible]	[illegible]	[illegible]	[illegible]	[illegible]	[illegible]
2014	Cook Islands	9240	4123	12500	[illegible]	[illegible]	[illegible]	[illegible]	[illegible]	[illegible]	[illegible]	[illegible]	[illegible]	[illegible]	[illegible]	[illegible]	[illegible]	[illegible]	[illegible]	[illegible]	[illegible]	[illegible]	[illegible]	[illegible]	[illegible]	[illegible]	[illegible]	[illegible]
2018	Colombia	23090247	7206203	95452000	[illegible]	[illegible]	[illegible]	[illegible]	[illegible]	[illegible]	[illegible]	[illegible]	[illegible]	[illegible]	[illegible]	[illegible]	[illegible]	[illegible]	[illegible]	[illegible]	[illegible]	[illegible]	[illegible]	[illegible]	[illegible]	[illegible]	[illegible]	[illegible]
2012	Comoros	231709	73496	61500	[illegible]	[illegible]	[illegible]	[illegible]	[illegible]	[illegible]	[illegible]	[illegible]	[illegible]	[illegible]	[illegible]	[illegible]	[illegible]	[illegible]	[illegible]	[illegible]	[illegible]	[illegible]	[illegible]	[illegible]	[illegible]	[illegible]	[illegible]	[illegible]
2015	Cabo Verde	210122	86861	205000	[illegible]	[illegible]	[illegible]	[illegible]	[illegible]	[illegible]	[illegible]	[illegible]	[illegible]	[illegible]	[illegible]	[illegible]	[illegible]	[illegible]	[illegible]	[illegible]	[illegible]	[illegible]	[illegible]	[illegible]	[illegible]	[illegible]	[illegible]	[illegible]
2018	Costa Rica	2348905	908959	7234000	[illegible]	[illegible]	[illegible]	[illegible]	[illegible]	[illegible]	[illegible]	[illegible]	[illegible]	[illegible]	[illegible]	[illegible]	[illegible]	[illegible]	[illegible]	[illegible]	[illegible]	[illegible]	[illegible]	[illegible]	[illegible]	[illegible]	[illegible]	[illegible]
2018	Cuba	6932992	3056695	47733000	[illegible]	[illegible]	[illegible]	[illegible]	[illegible]	[illegible]	[illegible]	[illegible]	[illegible]	[illegible]	[illegible]	[illegible]	[illegible]	[illegible]	[illegible]	[illegible]	[illegible]	[illegible]	[illegible]	[illegible]	[illegible]	[illegible]	[illegible]	[illegible]
2016	Cyprus	745533	257007	1141500	[illegible]	[illegible]	[illegible]	[illegible]	[illegible]	[illegible]	[illegible]	[illegible]	[illegible]	[illegible]	[illegible]	[illegible]	[illegible]	[illegible]	[illegible]	[illegible]	[illegible]	[illegible]	[illegible]	[illegible]	[illegible]	[illegible]	[illegible]	[illegible]
2019	Czech Republic	6903791	3311794	21997000	[illegible]	[illegible]	[illegible]	[illegible]	[illegible]	[illegible]	[illegible]	[illegible]	[illegible]	[illegible]	[illegible]	[illegible]	[illegible]	[illegible]	[illegible]	[illegible]	[illegible]	[illegible]	[illegible]	[illegible]	[illegible]	[illegible]	[illegible]	[illegible]
2018	Germany	53313842	20312971	178700500	[illegible]	[illegible]	[illegible]	[illegible]	[illegible]	[illegible]	[illegible]	[illegible]	[illegible]	[illegible]	[illegible]	[illegible]	[illegible]	[illegible]	[illegible]	[illegible]	[illegible]	[illegible]	[illegible]	[illegible]	[illegible]	[illegible]	[illegible]	[illegible]
2014	Djibouti	376661	122554	100500	[illegible]	[illegible]	[illegible]	[illegible]	[illegible]	[illegible]	[illegible]	[illegible]	[illegible]	[illegible]	[illegible]	[illegible]	[illegible]	[illegible]	[illegible]	[illegible]	[illegible]	[illegible]	[illegible]	[illegible]	[illegible]	[illegible]	[illegible]	[illegible]
2018	Dominica	35704	17654	39500	[illegible]	[illegible]	[illegible]	[illegible]	[illegible]	[illegible]	[illegible]	[illegible]	[illegible]	[illegible]	[illegible]	[illegible]	[illegible]	[illegible]	[illegible]	[illegible]	[illegible]	[illegible]	[illegible]	[illegible]	[illegible]	[illegible]	[illegible]	[illegible]
2018	Denmark	3433052	1498338	12150500	[illegible]	[illegible]	[illegible]	[illegible]	[illegible]	[illegible]	[illegible]	[illegible]	[illegible]	[illegible]	[illegible]	[illegible]	[illegible]	[illegible]	[illegible]	[illegible]	[illegible]	[illegible]	[illegible]	[illegible]	[illegible]	[illegible]	[illegible]	[illegible]
2019	Dominican Republic	4783422	2316705	7795500	[illegible]	[illegible]	[illegible]	[illegible]	[illegible]	[illegible]	[illegible]	[illegible]	[illegible]	[illegible]	[illegible]	[illegible]	[illegible]	[illegible]	[illegible]	[illegible]	[illegible]	[illegible]	[illegible]	[illegible]	[illegible]	[illegible]	[illegible]	[illegible]
2018	Algeria	19175359	6439470	36302000	[illegible]	[illegible]	[illegible]	[illegible]	[illegible]	[illegible]	[illegible]	[illegible]	[illegible]	[illegible]	[illegible]	[illegible]	[illegible]	[illegible]	[illegible]	[illegible]	[illegible]	[illegible]	[illegible]	[illegible]	[illegible]	[illegible]	[illegible]	[illegible]
2017	Ecuador	7209337	1856376	18631500	[illegible]	[illegible]	[illegible]	[illegible]	[illegible]	[illegible]	[illegible]	[illegible]	[illegible]	[illegible]	[illegible]	[illegible]	[illegible]	[illegible]	[illegible]	[illegible]	[illegible]	[illegible]	[illegible]	[illegible]	[illegible]	[illegible]	[illegible]	[illegible]
2019	Egypt	39639126	14135799	37461500	[illegible]	[illegible]	[illegible]	[illegible]	[illegible]	[illegible]	[illegible]	[illegible]	[illegible]	[illegible]	[illegible]	[illegible]	[illegible]	[illegible]	[illegible]	[illegible]	[illegible]	[illegible]	[illegible]	[illegible]	[illegible]	[illegible]	[illegible]	[illegible]
2004	Eritrea	1272263	314866	107500	[illegible]	[illegible]	[illegible]	[illegible]	[illegible]	[illegible]	[illegible]	[illegible]	[illegible]	[illegible]	[illegible]	[illegible]	[illegible]	[illegible]	[illegible]	[illegible]	[illegible]	[illegible]	[illegible]	[illegible]	[illegible]	[illegible]	[illegible]	[illegible]
2018	Spain	29532679	9730609	94083000	[illegible]	[illegible]	[illegible]	[illegible]	[illegible]	[illegible]	[illegible]	[illegible]	[illegible]	[illegible]	[illegible]	[illegible]	[illegible]	[illegible]	[illegible]	[illegible]	[illegible]	[illegible]	[illegible]	[illegible]	[illegible]	[illegible]	[illegible]	[illegible]
2017	Estonia	805570	366048	2284500	[illegible]	[illegible]	[illegible]	[illegible]	[illegible]	[illegible]	[illegible]	[illegible]	[illegible]	[illegible]	[illegible]	[illegible]	[illegible]	[illegible]	[illegible]	[illegible]	[illegible]	[illegible]	[illegible]	[illegible]	[illegible]	[illegible]	[illegible]	[illegible]
2018	Ethiopia	28319856	7141408	4197500	[illegible]	[illegible]	[illegible]	[illegible]	[illegible]	[illegible]	[illegible]	[illegible]	[illegible]	[illegible]	[illegible]	[illegible]	[illegible]	[illegible]	[illegible]	[illegible]	[illegible]	[illegible]	[illegible]	[illegible]	[illegible]	[illegible]	[illegible]	[illegible]
2018	Finland	3362706	1495735	12813500	[illegible]	[illegible]	[illegible]	[illegible]	[illegible]	[illegible]	[illegible]	[illegible]	[illegible]	[illegible]	[illegible]	[illegible]	[illegible]	[illegible]	[illegible]	[illegible]	[illegible]	[illegible]	[illegible]	[illegible]	[illegible]	[illegible]	[illegible]	[illegible]
2015	Fiji	402917	142813	373500	[illegible]	[illegible]	[illegible]	[illegible]	[illegible]	[illegible]	[illegible]	[illegible]	[illegible]	[illegible]	[illegible]	[illegible]	[illegible]	[illegible]	[illegible]	[illegible]	[illegible]	[illegible]	[illegible]	[illegible]	[illegible]	[illegible]	[illegible]	[illegible]
2018	France	38408460	14171949	212337000	[illegible]	[illegible]	[illegible]	[illegible]	[illegible]	[illegible]	[illegible]	[illegible]	[illegible]	[illegible]	[illegible]	[illegible]	[illegible]	[illegible]	[illegible]	[illegible]	[illegible]	[illegible]	[illegible]	[illegible]	[illegible]	[illegible]	[illegible]	[illegible]
2009	Micronesia (Federated States of)	37125	10528	10000	[illegible]	[illegible]	[illegible]	[illegible]	[illegible]	[illegible]	[illegible]	[illegible]	[illegible]	[illegible]	[illegible]	[illegible]	[illegible]	[illegible]	[illegible]	[illegible]	[illegible]	[illegible]	[illegible]	[illegible]	[illegible]	[illegible]	[illegible]	[illegible]
2017	Gabon	620029	203941	704000	[illegible]	[illegible]	[illegible]	[illegible]	[illegible]	[illegible]	[illegible]	[illegible]	[illegible]	[illegible]	[illegible]	[illegible]	[illegible]	[illegible]	[illegible]	[illegible]	[illegible]	[illegible]	[illegible]	[illegible]	[illegible]	[illegible]	[illegible]	[illegible]
2019	United Kingdom	39689815	12603926	196623500	[illegible]	[illegible]	[illegible]	[illegible]	[illegible]	[illegible]	[illegible]	[illegible]	[illegible]	[illegible]	[illegible]	[illegible]	[illegible]	[illegible]	[illegible]	[illegible]	[illegible]	[illegible]	[illegible]	[illegible]	[illegible]	[illegible]	[illegible]	[illegible]
2019	Georgia	2121215	1040747	14145500	[illegible]	[illegible]	[illegible]	[illegible]	[illegible]	[illegible]	[illegible]	[illegible]	[illegible]	[illegible]	[illegible]	[illegible]	[illegible]	[illegible]	[illegible]	[illegible]	[illegible]	[illegible]	[illegible]	[illegible]	[illegible]	[illegible]	[illegible]	[illegible]
2019	Ghana	10909379	3359726	1618000	[illegible]	[illegible]	[illegible]	[illegible]	[illegible]	[illegible]	[illegible]	[illegible]	[illegible]	[illegible]	[illegible]	[illegible]	[illegible]	[illegible]	[illegible]	[illegible]	[illegible]	[illegible]	[illegible]	[illegible]	[illegible]	[illegible]	[illegible]	[illegible]
2016	Guinea	3165456	1203536	488500	[illegible]	[illegible]	[illegible]	[illegible]	[illegible]	[illegible]	[illegible]	[illegible]	[illegible]	[illegible]	[illegible]	[illegible]	[illegible]	[illegible]	[illegible]	[illegible]	[illegible]	[illegible]	[illegible]	[illegible]	[illegible]	[illegible]	[illegible]	[illegible]
2019	Gambia	644674	213449	126000	[illegible]	[illegible]	[illegible]	[illegible]	[illegible]	[illegible]	[illegible]	[illegible]	[illegible]	[illegible]	[illegible]	[illegible]	[illegible]	[illegible]	[illegible]	[illegible]	[illegible]	[illegible]	[illegible]	[illegible]	[illegible]	[illegible]	[illegible]	[illegible]
2016	Guinea Bissau	491431	168269	113500	[illegible]	[illegible]	[illegible]	[illegible]	[illegible]	[illegible]	[illegible]	[illegible]	[illegible]	[illegible]	[illegible]	[illegible]	[illegible]	[illegible]	[illegible]	[illegible]	[illegible]	[illegible]	[illegible]	[illegible]	[illegible]	[illegible]	[illegible]	[illegible]
2017	Equatorial Guinea	344471	110183	253500	[illegible]	[illegible]	[illegible]	[illegible]	[illegible]	[illegible]	[illegible]	[illegible]	[illegible]	[illegible]	[illegible]	[illegible]	[illegible]	[illegible]	[illegible]	[illegible]	[illegible]	[illegible]	[illegible]	[illegible]	[illegible]	[illegible]	[illegible]	[illegible]
2018	Greece	6588661	2513316	32756500	[illegible]	[illegible]	[illegible]	[illegible]	[illegible]	[illegible]	[illegible]	[illegible]	[illegible]	[illegible]	[illegible]	[illegible]	[illegible]	[illegible]	[illegible]	[illegible]	[illegible]	[illegible]	[illegible]	[illegible]	[illegible]	[illegible]	[illegible]	[illegible]
2018	Grenada	51775	24608	80000	[illegible]	[illegible]	[illegible]	[illegible]	[illegible]	[illegible]	[illegible]	[illegible]	[illegible]	[illegible]	[illegible]	[illegible]	[illegible]	[illegible]	[illegible]	[illegible]	[illegible]	[illegible]	[illegible]	[illegible]	[illegible]	[illegible]	[illegible]	[illegible]
2018	Guatemala	6104079	1749541	3061000	[illegible]	[illegible]	[illegible]	[illegible]	[illegible]	[illegible]	[illegible]	[illegible]	[illegible]	[illegible]	[illegible]	[illegible]	[illegible]	[illegible]	[illegible]	[illegible]	[illegible]	[illegible]	[illegible]	[illegible]	[illegible]	[illegible]	[illegible]	[illegible]
2018	Guyana	328853	132266	708500	[illegible]	[illegible]	[illegible]	[illegible]	[illegible]	[illegible]	[illegible]	[illegible]	[illegible]	[illegible]	[illegible]	[illegible]	[illegible]	[illegible]	[illegible]	[illegible]	[illegible]	[illegible]	[illegible]	[illegible]	[illegible]	[illegible]	[illegible]	[illegible]
2017	Honduras	3280306	981881	1456500	[illegible]	[illegible]	[illegible]	[illegible]	[illegible]	[illegible]	[illegible]	[illegible]	[illegible]	[illegible]	[illegible]	[illegible]	[illegible]	[illegible]	[illegible]	[illegible]	[illegible]	[illegible]	[illegible]	[illegible]	[illegible]	[illegible]	[illegible]	[illegible]
2016	Croatia	2712539	1504051	6312000	[illegible]	[illegible]	[illegible]	[illegible]	[illegible]	[illegible]	[illegible]	[illegible]	[illegible]	[illegible]	[illegible]	[illegible]	[illegible]	[illegible]	[illegible]	[illegible]	[illegible]	[illegible]	[illegible]	[illegible]	[illegible]	[illegible]	[illegible]	[illegible]
2018	Haiti	4477302	1793878	1303000	[illegible]	[illegible]	[illegible]	[illegible]	[illegible]	[illegible]	[illegible]	[illegible]	[illegible]	[illegible]	[illegible]	[illegible]	[illegible]	[illegible]	[illegible]	[illegible]	[illegible]	[illegible]	[illegible]	[illegible]	[illegible]	[illegible]	[illegible]	[illegible]
2018	Hungary	6255462	3401434	16539000	[illegible]	[illegible]	[illegible]	[illegible]	[illegible]	[illegible]	[illegible]	[illegible]	[illegible]	[illegible]	[illegible]	[illegible]	[illegible]	[illegible]	[illegible]	[illegible]	[illegible]	[illegible]	[illegible]	[illegible]	[illegible]	[illegible]	[illegible]	[illegible]
2019	Indonesia	127028727	49808860	62931000	[illegible]	[illegible]	[illegible]	[illegible]	[illegible]	[illegible]	[illegible]	[illegible]	[illegible]	[illegible]	[illegible]	[illegible]	[illegible]	[illegible]	[illegible]	[illegible]	[illegible]	[illegible]	[illegible]	[illegible]	[illegible]	[illegible]	[illegible]	[illegible]
2019	India	617122977	185575848	634086000	[illegible]	[illegible]	[illegible]	[illegible]	[illegible]	[illegible]	[illegible]	[illegible]	[illegible]	[illegible]	[illegible]	[illegible]	[illegible]	[illegible]	[illegible]	[illegible]	[illegible]	[illegible]	[illegible]	[illegible]	[illegible]	[illegible]	[illegible]	[illegible]
2019	Ireland	2831948	999773	8183000	[illegible]	[illegible]	[illegible]	[illegible]	[illegible]	[illegible]	[illegible]	[illegible]	[illegible]	[illegible]	[illegible]	[illegible]	[illegible]	[illegible]	[illegible]	[illegible]	[illegible]	[illegible]	[illegible]	[illegible]	[illegible]	[illegible]	[illegible]	[illegible]
2018	Iran	43072254	10170699	64802000	[illegible]	[illegible]	[illegible]	[illegible]	[illegible]	[illegible]	[illegible]	[illegible]	[illegible]	[illegible]	[illegible]	[illegible]	[illegible]	[illegible]	[illegible]	[illegible]	[illegible]	[illegible]	[illegible]	[illegible]	[illegible]	[illegible]	[illegible]	[illegible]
2018	Iraq	14696978	6300402	13604000	[illegible]	[illegible]	[illegible]	[illegible]	[illegible]	[illegible]	[illegible]	[illegible]	[illegible]	[illegible]	[illegible]	[illegible]	[illegible]	[illegible]	[illegible]	[illegible]	[illegible]	[illegible]	[illegible]	[illegible]	[illegible]	[illegible]	[illegible]	[illegible]
2019	Iceland	192199	63521	702000	[illegible]	[illegible]	[illegible]	[illegible]	[illegible]	[illegible]	[illegible]	[illegible]	[illegible]	[illegible]	[illegible]	[illegible]	[illegible]	[illegible]	[illegible]	[illegible]	[illegible]	[illegible]	[illegible]	[illegible]	[illegible]	[illegible]	[illegible]	[illegible]
2019	Israel	4433019	1416402	23286500	[illegible]	[illegible]	[illegible]	[illegible]	[illegible]	[illegible]	[illegible]	[illegible]	[illegible]	[illegible]	[illegible]	[illegible]	[illegible]	[illegible]	[illegible]	[illegible]	[illegible]	[illegible]	[illegible]	[illegible]	[illegible]	[illegible]	[illegible]	[illegible]
2019	Italy	38988185	16543201	242595000	[illegible]	[illegible]	[illegible]	[illegible]	[illegible]	[illegible]	[illegible]	[illegible]	[illegible]	[illegible]	[illegible]	[illegible]	[illegible]	[illegible]	[illegible]	[illegible]	[illegible]	[illegible]	[illegible]	[illegible]	[illegible]	[illegible]	[illegible]	[illegible]
2016	Jamaica	1291219	592779	661000	[illegible]	[illegible]	[illegible]	[illegible]	[illegible]	[illegible]	[illegible]	[illegible]	[illegible]	[illegible]	[illegible]	[illegible]	[illegible]	[illegible]	[illegible]	[illegible]	[illegible]	[illegible]	[illegible]	[illegible]	[illegible]	[illegible]	[illegible]	[illegible]
2017	Jordan	4033280	1340267	11369500	[illegible]	[illegible]	[illegible]	[illegible]	[illegible]	[illegible]	[illegible]	[illegible]	[illegible]	[illegible]	[illegible]	[illegible]	[illegible]	[illegible]	[illegible]	[illegible]	[illegible]	[illegible]	[illegible]	[illegible]	[illegible]	[illegible]	[illegible]	[illegible]
2018	Japan	82597155	33277029	157703000	[illegible]	[illegible]	[illegible]	[illegible]	[illegible]	[illegible]	[illegible]	[illegible]	[illegible]	[illegible]	[illegible]	[illegible]	[illegible]	[illegible]	[illegible]	[illegible]	[illegible]	[illegible]	[illegible]	[illegible]	[illegible]	[illegible]	[illegible]	[illegible]
2014	Kazakhstan	8153922	3408498	34432000	[illegible]	[illegible]	[illegible]	[illegible]	[illegible]	[illegible]	[illegible]	[illegible]	[illegible]	[illegible]	[illegible]	[illegible]	[illegible]	[illegible]	[illegible]	[illegible]	[illegible]	[illegible]	[illegible]	[illegible]	[illegible]	[illegible]	[illegible]	[illegible]
2018	Kenya	15289748	4423478	4021000	[illegible]	[illegible]	[illegible]	[illegible]	[illegible]	[illegible]	[illegible]	[illegible]	[illegible]	[illegible]	[illegible]	[illegible]	[illegible]	[illegible]	[illegible]	[illegible]	[illegible]	[illegible]	[illegible]	[illegible]	[illegible]	[illegible]	[illegible]	[illegible]
2014	Kyrgyzstan	2333084	869113	6467000	[illegible]	[illegible]	[illegible]	[illegible]	[illegible]	[illegible]	[illegible]	[illegible]	[illegible]	[illegible]	[illegible]	[illegible]	[illegible]	[illegible]	[illegible]	[illegible]	[illegible]	[illegible]	[illegible]	[illegible]	[illegible]	[illegible]	[illegible]	[illegible]
2014	Cambodia	5862246	1285328	1472000	[illegible]	[illegible]	[illegible]	[illegible]	[illegible]	[illegible]	[illegible]	[illegible]	[illegible]	[illegible]	[illegible]	[illegible]	[illegible]	[illegible]	[illegible]	[illegible]	[illegible]	[illegible]	[illegible]	[illegible]	[illegible]	[illegible]	[illegible]	[illegible]
2013	Kiribati	39042	13621	11000	[illegible]	[illegible]	[illegible]	[illegible]	[illegible]	[illegible]	[illegible]	[illegible]	[illegible]	[illegible]	[illegible]	[illegible]	[illegible]	[illegible]	[illegible]	[illegible]	[illegible]	[illegible]	[illegible]	[illegible]	[illegible]	[illegible]	[illegible]	[illegible]
2018	Saint Kitts and Nevis	32820	14595	72500	[illegible]	[illegible]	[illegible]	[illegible]	[illegible]	[illegible]	[illegible]	[illegible]	[illegible]	[illegible]	[illegible]	[illegible]	[illegible]	[illegible]	[illegible]	[illegible]	[illegible]	[illegible]	[illegible]	[illegible]	[illegible]	[illegible]	[illegible]	[illegible]
2018	South Korea	34629516	10642712	61615000	[illegible]	[illegible]	[illegible]	[illegible]	[illegible]	[illegible]	[illegible]	[illegible]	[illegible]	[illegible]	[illegible]	[illegible]	[illegible]	[illegible]	[illegible]	[illegible]	[illegible]	[illegible]	[illegible]	[illegible]	[illegible]	[illegible]	[illegible]	[illegible]
2015	Kuwait	2049332	665243	5075000	[illegible]	[illegible]	[illegible]	[illegible]	[illegible]	[illegible]	[illegible]	[illegible]	[illegible]	[illegible]	[illegible]	[illegible]	[illegible]	[illegible]	[illegible]	[illegible]	[illegible]	[illegible]	[illegible]	[illegible]	[illegible]	[illegible]	[illegible]	[illegible]
2017	Lao PDR	2628189	674019	1295500	[illegible]	[illegible]	[illegible]	[illegible]	[illegible]	[illegible]	[illegible]	[illegible]	[illegible]	[illegible]	[illegible]	[illegible]	[illegible]	[illegible]	[illegible]	[illegible]	[illegible]	[illegible]	[illegible]	[illegible]	[illegible]	[illegible]	[illegible]	[illegible]
2018	Lebanon	2451477	908808	7215500	[illegible]	[illegible]	[illegible]	[illegible]	[illegible]	[illegible]	[illegible]	[illegible]	[illegible]	[illegible]	[illegible]	[illegible]	[illegible]	[illegible]	[illegible]	[illegible]	[illegible]	[illegible]	[illegible]	[illegible]	[illegible]	[illegible]	[illegible]	[illegible]
2015	Liberia	1350821	489862	84000	[illegible]	[illegible]	[illegible]	[illegible]	[illegible]	[illegible]	[illegible]	[illegible]	[illegible]	[illegible]	[illegible]	[illegible]	[illegible]	[illegible]	[illegible]	[illegible]	[illegible]	[illegible]	[illegible]	[illegible]	[illegible]	[illegible]	[illegible]	[illegible]
2017	Libya	3237692	1209765	6878500	[illegible]	[illegible]	[illegible]	[illegible]	[illegible]	[illegible]	[illegible]	[illegible]	[illegible]	[illegible]	[illegible]	[illegible]	[illegible]	[illegible]	[illegible]	[illegible]	[illegible]	[illegible]	[illegible]	[illegible]	[illegible]	[illegible]	[illegible]	[illegible]
2017	Saint Lucia	94136	37838	58000	[illegible]	[illegible]	[illegible]	[illegible]	[illegible]	[illegible]	[illegible]	[illegible]	[illegible]	[illegible]	[illegible]	[illegible]	[illegible]	[illegible]	[illegible]	[illegible]	[illegible]	[illegible]	[illegible]	[illegible]	[illegible]	[illegible]	[illegible]	[illegible]
2019	Sri Lanka	11517772	4364940	12302500	[illegible]	[illegible]	[illegible]	[illegible]	[illegible]	[illegible]	[illegible]	[illegible]	[illegible]	[illegible]	[illegible]	[illegible]	[illegible]	[illegible]	[illegible]	[illegible]	[illegible]	[illegible]	[illegible]	[illegible]	[illegible]	[illegible]	[illegible]	[illegible]
2010	Lesotho	658809	253460	69000	[illegible]	[illegible]	[illegible]	[illegible]	[illegible]	[illegible]	[illegible]	[illegible]	[illegible]	[illegible]	[illegible]	[illegible]	[illegible]	[illegible]	[illegible]	[illegible]	[illegible]	[illegible]	[illegible]	[illegible]	[illegible]	[illegible]	[illegible]	[illegible]
2019	Lithuania	1723342	950912	6954000	[illegible]	[illegible]	[illegible]	[illegible]	[illegible]	[illegible]	[illegible]	[illegible]	[illegible]	[illegible]	[illegible]	[illegible]	[illegible]	[illegible]	[illegible]	[illegible]	[illegible]	[illegible]	[illegible]	[illegible]	[illegible]	[illegible]	[illegible]	[illegible]
2017	Luxembourg	357925	124756	890000	[illegible]	[illegible]	[illegible]	[illegible]	[illegible]	[illegible]	[illegible]	[illegible]	[illegible]	[illegible]	[illegible]	[illegible]	[illegible]	[illegible]	[illegible]	[illegible]	[illegible]	[illegible]	[illegible]	[illegible]	[illegible]	[illegible]	[illegible]	[illegible]
2018	Latvia	1212840	614495	3183500	[illegible]	[illegible]	[illegible]	[illegible]	[illegible]	[illegible]	[illegible]	[illegible]	[illegible]	[illegible]	[illegible]	[illegible]	[illegible]	[illegible]	[illegible]	[illegible]	[illegible]	[illegible]	[illegible]	[illegible]	[illegible]	[illegible]	[illegible]	[illegible]
2017	Morocco	16314453	5739414	13001500	[illegible]	[illegible]	[illegible]	[illegible]	[illegible]	[illegible]	[illegible]	[illegible]	[illegible]	[illegible]	[illegible]	[illegible]	[illegible]	[illegible]	[illegible]	[illegible]	[illegible]	[illegible]	[illegible]	[illegible]	[illegible]	[illegible]	[illegible]	[illegible]
2019	Moldova	2314472	1153256	5172500	[illegible]	[illegible]	[illegible]	[illegible]	[illegible]	[illegible]	[illegible]	[illegible]	[illegible]	[illegible]	[illegible]	[illegible]	[illegible]	[illegible]	[illegible]	[illegible]	[illegible]	[illegible]	[illegible]	[illegible]	[illegible]	[illegible]	[illegible]	[illegible]
2014	Madagascar	6798364	2418518	2137500	[illegible]	[illegible]	[illegible]	[illegible]	[illegible]	[illegible]	[illegible]	[illegible]	[illegible]	[illegible]	[illegible]	[illegible]	[illegible]	[illegible]	[illegible]	[illegible]	[illegible]	[illegible]	[illegible]	[illegible]	[illegible]	[illegible]	[illegible]	[illegible]
2018	Maldives	237121	62575	440500	[illegible]	[illegible]	[illegible]	[illegible]	[illegible]	[illegible]	[illegible]	[illegible]	[illegible]	[illegible]	[illegible]	[illegible]	[illegible]	[illegible]	[illegible]	[illegible]	[illegible]	[illegible]	[illegible]	[illegible]	[illegible]	[illegible]	[illegible]	[illegible]
2018	Mexico	57714057	18260211	305985000	[illegible]	[illegible]	[illegible]	[illegible]	[illegible]	[illegible]	[illegible]	[illegible]	[illegible]	[illegible]	[illegible]	[illegible]	[illegible]	[illegible]	[illegible]	[illegible]	[illegible]	[illegible]	[illegible]	[illegible]	[illegible]	[illegible]	[illegible]	[illegible]
2012	Marshall Islands	20024	5974	12000	[illegible]	[illegible]	[illegible]	[illegible]	[illegible]	[illegible]	[illegible]	[illegible]	[illegible]	[illegible]	[illegible]	[illegible]	[illegible]	[illegible]	[illegible]	[illegible]	[illegible]	[illegible]	[illegible]	[illegible]	[illegible]	[illegible]	[illegible]	[illegible]
2015	Macedonia (TFYR)	1279988	622345	2987500	[illegible]	[illegible]	[illegible]	[illegible]	[illegible]	[illegible]	[illegible]	[illegible]	[illegible]	[illegible]	[illegible]	[illegible]	[illegible]	[illegible]	[illegible]	[illegible]	[illegible]	[illegible]	[illegible]	[illegible]	[illegible]	[illegible]	[illegible]	[illegible]
2018	Mali	5338919	1609266	1227000	[illegible]	[illegible]	[illegible]	[illegible]	[illegible]	[illegible]	[illegible]	[illegible]	[illegible]	[illegible]	[illegible]	[illegible]	[illegible]	[illegible]	[illegible]	[illegible]	[illegible]	[illegible]	[illegible]	[illegible]	[illegible]	[illegible]	[illegible]	[illegible]
2015	Malta	270090	101967	620000	[illegible]	[illegible]	[illegible]	[illegible]	[illegible]	[illegible]	[illegible]	[illegible]	[illegible]	[illegible]	[illegible]	[illegible]	[illegible]	[illegible]	[illegible]	[illegible]	[illegible]	[illegible]	[illegible]	[illegible]	[illegible]	[illegible]	[illegible]	[illegible]
2019	Myanmar	25099237	9306963	19913000	[illegible]	[illegible]	[illegible]	[illegible]	[illegible]	[illegible]	[illegible]	[illegible]	[illegible]	[illegible]	[illegible]	[illegible]	[illegible]	[illegible]	[illegible]	[illegible]	[illegible]	[illegible]	[illegible]	[illegible]	[illegible]	[illegible]	[illegible]	[illegible]
2018	Montenegro	369430	184830	865000	[illegible]	[illegible]	[illegible]	[illegible]	[illegible]	[illegible]	[illegible]	[illegible]	[illegible]	[illegible]	[illegible]	[illegible]	[illegible]	[illegible]	[illegible]	[illegible]	[illegible]	[illegible]	[illegible]	[illegible]	[illegible]	[illegible]	[illegible]	[illegible]
2018	Mongolia	1537917	605566	6105500	[illegible]	[illegible]	[illegible]	[illegible]	[illegible]	[illegible]	[illegible]	[illegible]	[illegible]	[illegible]	[illegible]	[illegible]	[illegible]	[illegible]	[illegible]	[illegible]	[illegible]	[illegible]	[illegible]	[illegible]	[illegible]	[illegible]	[illegible]	[illegible]
2019	Mozambique	7546168	2644652	1285000	[illegible]	[illegible]	[illegible]	[illegible]	[illegible]	[illegible]	[illegible]	[illegible]	[illegible]	[illegible]	[illegible]	[illegible]	[illegible]	[illegible]	[illegible]	[illegible]	[illegible]	[illegible]	[illegible]	[illegible]	[illegible]	[illegible]	[illegible]	[illegible]
2018	Mauritania	1209778	413367	410500	[illegible]	[illegible]	[illegible]	[illegible]	[illegible]	[illegible]	[illegible]	[illegible]	[illegible]	[illegible]	[illegible]	[illegible]	[illegible]	[illegible]	[illegible]	[illegible]	[illegible]	[illegible]	[illegible]	[illegible]	[illegible]	[illegible]	[illegible]	[illegible]
2018	Mauritius	741413	273535	1605000	[illegible]	[illegible]	[illegible]	[illegible]	[illegible]	[illegible]	[illegible]	[illegible]	[illegible]	[illegible]	[illegible]	[illegible]	[illegible]	[illegible]	[illegible]	[illegible]	[illegible]	[illegible]	[illegible]	[illegible]	[illegible]	[illegible]	[illegible]	[illegible]
2018	Malawi	4897139	1239540	324500	[illegible]	[illegible]	[illegible]	[illegible]	[illegible]	[illegible]	[illegible]	[illegible]	[illegible]	[illegible]	[illegible]	[illegible]	[illegible]	[illegible]	[illegible]	[illegible]	[illegible]	[illegible]	[illegible]	[illegible]	[illegible]	[illegible]	[illegible]	[illegible]
2015	Malaysia	13329165	5245463	23245500	[illegible]	[illegible]	[illegible]	[illegible]	[illegible]	[illegible]	[illegible]	[illegible]	[illegible]	[illegible]	[illegible]	[illegible]	[illegible]	[illegible]	[illegible]	[illegible]	[illegible]	[illegible]	[illegible]	[illegible]	[illegible]	[illegible]	[illegible]	[illegible]
2017	Namibia	816526	333298	710500	[illegible]	[illegible]	[illegible]	[illegible]	[illegible]	[illegible]	[illegible]	[illegible]	[illegible]	[illegible]	[illegible]	[illegible]	[illegible]	[illegible]	[illegible]	[illegible]	[illegible]	[illegible]	[illegible]	[illegible]	[illegible]	[illegible]	[illegible]	[illegible]
2016	Niger	4718993	1870563	450000	[illegible]	[illegible]	[illegible]	[illegible]	[illegible]	[illegible]	[illegible]	[illegible]	[illegible]	[illegible]	[illegible]	[illegible]	[illegible]	[illegible]	[illegible]	[illegible]	[illegible]	[illegible]	[illegible]	[illegible]	[illegible]	[illegible]	[illegible]	[illegible]
2018	Nigeria	58706722	19025951	37271500	[illegible]	[illegible]	[illegible]	[illegible]	[illegible]	[illegible]	[illegible]	[illegible]	[illegible]	[illegible]	[illegible]	[illegible]	[illegible]	[illegible]	[illegible]	[illegible]	[illegible]	[illegible]	[illegible]	[illegible]	[illegible]	[illegible]	[illegible]	[illegible]
2018	Nicaragua	2546215	835574	5381000	[illegible]	[illegible]	[illegible]	[illegible]	[illegible]	[illegible]	[illegible]	[illegible]	[illegible]	[illegible]	[illegible]	[illegible]	[illegible]	[illegible]	[illegible]	[illegible]	[illegible]	[illegible]	[illegible]	[illegible]	[illegible]	[illegible]	[illegible]	[illegible]
2008	Niue	796	294	1500	[illegible]	[illegible]	[illegible]	[illegible]	[illegible]	[illegible]	[illegible]	[illegible]	[illegible]	[illegible]	[illegible]	[illegible]	[illegible]	[illegible]	[illegible]	[illegible]	[illegible]	[illegible]	[illegible]	[illegible]	[illegible]	[illegible]	[illegible]	[illegible]
2018	Netherlands	10378301	4039217	31616500	[illegible]	[illegible]	[illegible]	[illegible]	[illegible]	[illegible]	[illegible]	[illegible]	[illegible]	[illegible]	[illegible]	[illegible]	[illegible]	[illegible]	[illegible]	[illegible]	[illegible]	[illegible]	[illegible]	[illegible]	[illegible]	[illegible]	[illegible]	[illegible]
2019	Norway	3145249	1142052	13138000	[illegible]	[illegible]	[illegible]	[illegible]	[illegible]	[illegible]	[illegible]	[illegible]	[illegible]	[illegible]	[illegible]	[illegible]	[illegible]	[illegible]	[illegible]	[illegible]	[illegible]	[illegible]	[illegible]	[illegible]	[illegible]	[illegible]	[illegible]	[illegible]
2019	Nepal	11981271	4295016	11573000	[illegible]	[illegible]	[illegible]	[illegible]	[illegible]	[illegible]	[illegible]	[illegible]	[illegible]	[illegible]	[illegible]	[illegible]	[illegible]	[illegible]	[illegible]	[illegible]	[illegible]	[illegible]	[illegible]	[illegible]	[illegible]	[illegible]	[illegible]	[illegible]
2015	Nauru	3451	1277	7000	[illegible]	[illegible]	[illegible]	[illegible]	[illegible]	[illegible]	[illegible]	[illegible]	[illegible]	[illegible]	[illegible]	[illegible]	[illegible]	[illegible]	[illegible]	[illegible]	[illegible]	[illegible]	[illegible]	[illegible]	[illegible]	[illegible]	[illegible]	[illegible]
2018	New Zealand	2532803	912633	8112000	[illegible]	[illegible]	[illegible]	[illegible]	[illegible]	[illegible]	[illegible]	[illegible]	[illegible]	[illegible]	[illegible]	[illegible]	[illegible]	[illegible]	[illegible]	[illegible]	[illegible]	[illegible]	[illegible]	[illegible]	[illegible]	[illegible]	[illegible]	[illegible]
2019	Oman	2221166	812324	4801000	[illegible]	[illegible]	[illegible]	[illegible]	[illegible]	[illegible]	[illegible]	[illegible]	[illegible]	[illegible]	[illegible]	[illegible]	[illegible]	[illegible]	[illegible]	[illegible]	[illegible]	[illegible]	[illegible]	[illegible]	[illegible]	[illegible]	[illegible]	[illegible]
2019	Pakistan	72438491	29904354	121049500	[illegible]	[illegible]	[illegible]	[illegible]	[illegible]	[illegible]	[illegible]	[illegible]	[illegible]	[illegible]	[illegible]	[illegible]	[illegible]	[illegible]	[illegible]	[illegible]	[illegible]	[illegible]	[illegible]	[illegible]	[illegible]	[illegible]	[illegible]	[illegible]
2019	Panama	1910026	686627	3466000	[illegible]	[illegible]	[illegible]	[illegible]	[illegible]	[illegible]	[illegible]	[illegible]	[illegible]	[illegible]	[illegible]	[illegible]	[illegible]	[illegible]	[illegible]	[illegible]	[illegible]	[illegible]	[illegible]	[illegible]	[illegible]	[illegible]	[illegible]	[illegible]
2018	Peru	15296499	3066021	13115500	[illegible]	[illegible]	[illegible]	[illegible]	[illegible]	[illegible]	[illegible]	[illegible]	[illegible]	[illegible]	[illegible]	[illegible]	[illegible]	[illegible]	[illegible]	[illegible]	[illegible]	[illegible]	[illegible]	[illegible]	[illegible]	[illegible]	[illegible]	[illegible]
2017	Philippines	42996771	13780194	31570500	[illegible]	[illegible]	[illegible]	[illegible]	[illegible]	[illegible]	[illegible]	[illegible]	[illegible]	[illegible]	[illegible]	[illegible]	[illegible]	[illegible]	[illegible]	[illegible]	[illegible]	[illegible]	[illegible]	[illegible]	[illegible]	[illegible]	[illegible]	[illegible]
2014	Palau	10133	4449	12500	[illegible]	[illegible]	[illegible]	[illegible]	[illegible]	[illegible]	[illegible]	[illegible]	[illegible]	[illegible]	[illegible]	[illegible]	[illegible]	[illegible]	[illegible]	[illegible]	[illegible]	[illegible]	[illegible]	[illegible]	[illegible]	[illegible]	[illegible]	[illegible]
2019	Papua New Guinea	3430644	864706	290000	[illegible]	[illegible]	[illegible]	[illegible]	[illegible]	[illegible]	[illegible]	[illegible]	[illegible]	[illegible]	[illegible]	[illegible]	[illegible]	[illegible]	[illegible]	[illegible]	[illegible]	[illegible]	[illegible]	[illegible]	[illegible]	[illegible]	[illegible]	[illegible]
2017	Poland	24151986	12803143	45142000	[illegible]	[illegible]	[illegible]	[illegible]	[illegible]	[illegible]	[illegible]	[illegible]	[illegible]	[illegible]	[illegible]	[illegible]	[illegible]	[illegible]	[illegible]	[illegible]	[illegible]	[illegible]	[illegible]	[illegible]	[illegible]	[illegible]	[illegible]	[illegible]
2017	North Korea	14393603	4013737	46833500	[illegible]	[illegible]	[illegible]	[illegible]	[illegible]	[illegible]	[illegible]	[illegible]	[illegible]	[illegible]	[illegible]	[illegible]	[illegible]	[illegible]	[illegible]	[illegible]	[illegible]	[illegible]	[illegible]	[illegible]	[illegible]	[illegible]	[illegible]	[illegible]
2018	Portugal	6851889	2773230	27239000	[illegible]	[illegible]	[illegible]	[illegible]	[illegible]	[illegible]	[illegible]	[illegible]	[illegible]	[illegible]	[illegible]	[illegible]	[illegible]	[illegible]	[illegible]	[illegible]	[illegible]	[illegible]	[illegible]	[illegible]	[illegible]	[illegible]	[illegible]	[illegible]
2018	Paraguay	2878509	1568713	4710500	[illegible]	[illegible]	[illegible]	[illegible]	[illegible]	[illegible]	[illegible]	[illegible]	[illegible]	[illegible]	[illegible]	[illegible]	[illegible]	[illegible]	[illegible]	[illegible]	[illegible]	[illegible]	[illegible]	[illegible]	[illegible]	[illegible]	[illegible]	[illegible]
2018	Qatar	1529843	469849	3456500	[illegible]	[illegible]	[illegible]	[illegible]	[illegible]	[illegible]	[illegible]	[illegible]	[illegible]	[illegible]	[illegible]	[illegible]	[illegible]	[illegible]	[illegible]	[illegible]	[illegible]	[illegible]	[illegible]	[illegible]	[illegible]	[illegible]	[illegible]	[illegible]
2017	Romania	12308621	6590757	29291500	[illegible]	[illegible]	[illegible]	[illegible]	[illegible]	[illegible]	[illegible]	[illegible]	[illegible]	[illegible]	[illegible]	[illegible]	[illegible]	[illegible]	[illegible]	[illegible]	[illegible]	[illegible]	[illegible]	[illegible]	[illegible]	[illegible]	[illegible]	[illegible]
2018	Russian Federation	90358764	43672740	323496500	[illegible]	[illegible]	[illegible]	[illegible]	[illegible]	[illegible]	[illegible]	[illegible]	[illegible]	[illegible]	[illegible]	[illegible]	[illegible]	[illegible]	[illegible]	[illegible]	[illegible]	[illegible]	[illegible]	[illegible]	[illegible]	[illegible]	[illegible]	[illegible]
2019	Rwanda	4167889	1102469	746000	[illegible]	[illegible]	[illegible]	[illegible]	[illegible]	[illegible]	[illegible]	[illegible]	[illegible]	[illegible]	[illegible]	[illegible]	[illegible]	[illegible]	[illegible]	[illegible]	[illegible]	[illegible]	[illegible]	[illegible]	[illegible]	[illegible]	[illegible]	[illegible]
2018	Saudi Arabia	17984232	5043208	44011500	[illegible]	[illegible]	[illegible]	[illegible]	[illegible]	[illegible]	[illegible]	[illegible]	[illegible]	[illegible]	[illegible]	[illegible]	[illegible]	[illegible]	[illegible]	[illegible]	[illegible]	[illegible]	[illegible]	[illegible]	[illegible]	[illegible]	[illegible]	[illegible]
2017	Sudan	12055899	4668047	5341500	[illegible]	[illegible]	[illegible]	[illegible]	[illegible]	[illegible]	[illegible]	[illegible]	[illegible]	[illegible]	[illegible]	[illegible]	[illegible]	[illegible]	[illegible]	[illegible]	[illegible]	[illegible]	[illegible]	[illegible]	[illegible]	[illegible]	[illegible]	[illegible]
2019	Senegal	4637778	1678746	717500	[illegible]	[illegible]	[illegible]	[illegible]	[illegible]	[illegible]	[illegible]	[illegible]	[illegible]	[illegible]	[illegible]	[illegible]	[illegible]	[illegible]	[illegible]	[illegible]	[illegible]	[illegible]	[illegible]	[illegible]	[illegible]	[illegible]	[illegible]	[illegible]
2016	Singapore	3563630	1223024	6483500	[illegible]	[illegible]	[illegible]	[illegible]	[illegible]	[illegible]	[illegible]	[illegible]	[illegible]	[illegible]	[illegible]	[illegible]	[illegible]	[illegible]	[illegible]	[illegible]	[illegible]	[illegible]	[illegible]	[illegible]	[illegible]	[illegible]	[illegible]	[illegible]
2016	Solomon Islands	203974	49898	60000	[illegible]	[illegible]	[illegible]	[illegible]	[illegible]	[illegible]	[illegible]	[illegible]	[illegible]	[illegible]	[illegible]	[illegible]	[illegible]	[illegible]	[illegible]	[illegible]	[illegible]	[illegible]	[illegible]	[illegible]	[illegible]	[illegible]	[illegible]	[illegible]
2018	Sierra Leone	2361147	876629	283000	[illegible]	[illegible]	[illegible]	[illegible]	[illegible]	[illegible]	[illegible]	[illegible]	[illegible]	[illegible]	[illegible]	[illegible]	[illegible]	[illegible]	[illegible]	[illegible]	[illegible]	[illegible]	[illegible]	[illegible]	[illegible]	[illegible]	[illegible]	[illegible]
2018	El Salvador	2620644	859903	9213500	[illegible]	[illegible]	[illegible]	[illegible]	[illegible]	[illegible]	[illegible]	[illegible]	[illegible]	[illegible]	[illegible]	[illegible]	[illegible]	[illegible]	[illegible]	[illegible]	[illegible]	[illegible]	[illegible]	[illegible]	[illegible]	[illegible]	[illegible]	[illegible]
2014	Somalia	4369645	1526645	154500	[illegible]	[illegible]	[illegible]	[illegible]	[illegible]	[illegible]	[illegible]	[illegible]	[illegible]	[illegible]	[illegible]	[illegible]	[illegible]	[illegible]	[illegible]	[illegible]	[illegible]	[illegible]	[illegible]	[illegible]	[illegible]	[illegible]	[illegible]	[illegible]
2016	Serbia	5371965	2824423	13781500	[illegible]	[illegible]	[illegible]	[illegible]	[illegible]	[illegible]	[illegible]	[illegible]	[illegible]	[illegible]	[illegible]	[illegible]	[illegible]	[illegible]	[illegible]	[illegible]	[illegible]	[illegible]	[illegible]	[illegible]	[illegible]	[illegible]	[illegible]	[illegible]
2015	Sao Tome and Principe	61889	25776	31500	[illegible]	[illegible]	[illegible]	[illegible]	[illegible]	[illegible]	[illegible]	[illegible]	[illegible]	[illegible]	[illegible]	[illegible]	[illegible]	[illegible]	[illegible]	[illegible]	[illegible]	[illegible]	[illegible]	[illegible]	[illegible]	[illegible]	[illegible]	[illegible]
2018	Suriname	281877	119875	236000	[illegible]	[illegible]	[illegible]	[illegible]	[illegible]	[illegible]	[illegible]	[illegible]	[illegible]	[illegible]	[illegible]	[illegible]	[illegible]	[illegible]	[illegible]	[illegible]	[illegible]	[illegible]	[illegible]	[illegible]	[illegible]	[illegible]	[illegible]	[illegible]
2018	Slovakia	3451727	1630247	9589000	[illegible]	[illegible]	[illegible]	[illegible]	[illegible]	[illegible]	[illegible]	[illegible]	[illegible]	[illegible]	[illegible]	[illegible]	[illegible]	[illegible]	[illegible]	[illegible]	[illegible]	[illegible]	[illegible]	[illegible]	[illegible]	[illegible]	[illegible]	[illegible]
2018	Slovenia	1339762	690119	3295500	[illegible]	[illegible]	[illegible]	[illegible]	[illegible]	[illegible]	[illegible]	[illegible]	[illegible]	[illegible]	[illegible]	[illegible]	[illegible]	[illegible]	[illegible]	[illegible]	[illegible]	[illegible]	[illegible]	[illegible]	[illegible]	[illegible]	[illegible]	[illegible]
2017	Sweden	5887758	2277690	21449000	[illegible]	[illegible]	[illegible]	[illegible]	[illegible]	[illegible]	[illegible]	[illegible]	[illegible]	[illegible]	[illegible]	[illegible]	[illegible]	[illegible]	[illegible]	[illegible]	[illegible]	[illegible]	[illegible]	[illegible]	[illegible]	[illegible]	[illegible]	[illegible]
2016	Swaziland	358212	139804	53500	[illegible]	[illegible]	[illegible]	[illegible]	[illegible]	[illegible]	[illegible]	[illegible]	[illegible]	[illegible]	[illegible]	[illegible]	[illegible]	[illegible]	[illegible]	[illegible]	[illegible]	[illegible]	[illegible]	[illegible]	[illegible]	[illegible]	[illegible]	[illegible]
2018	Seychelles	55484	24858	120000	[illegible]	[illegible]	[illegible]	[illegible]	[illegible]	[illegible]	[illegible]	[illegible]	[illegible]	[illegible]	[illegible]	[illegible]	[illegible]	[illegible]	[illegible]	[illegible]	[illegible]	[illegible]	[illegible]	[illegible]	[illegible]	[illegible]	[illegible]	[illegible]
2016	Syrian Arab Republic	6512253	2400903	11242500	[illegible]	[illegible]	[illegible]	[illegible]	[illegible]	[illegible]	[illegible]	[illegible]	[illegible]	[illegible]	[illegible]	[illegible]	[illegible]	[illegible]	[illegible]	[illegible]	[illegible]	[illegible]	[illegible]	[illegible]	[illegible]	[illegible]	[illegible]	[illegible]
2019	Chad	3808907	1289329	432500	[illegible]	[illegible]	[illegible]	[illegible]	[illegible]	[illegible]	[illegible]	[illegible]	[illegible]	[illegible]	[illegible]	[illegible]	[illegible]	[illegible]	[illegible]	[illegible]	[illegible]	[illegible]	[illegible]	[illegible]	[illegible]	[illegible]	[illegible]	[illegible]
2019	Togo	2601663	841587	313500	[illegible]	[illegible]	[illegible]	[illegible]	[illegible]	[illegible]	[illegible]	[illegible]	[illegible]	[illegible]	[illegible]	[illegible]	[illegible]	[illegible]	[illegible]	[illegible]	[illegible]	[illegible]	[illegible]	[illegible]	[illegible]	[illegible]	[illegible]	[illegible]
2019	Thailand	43538238	13985484	31987000	[illegible]	[illegible]	[illegible]	[illegible]	[illegible]	[illegible]	[illegible]	[illegible]	[illegible]	[illegible]	[illegible]	[illegible]	[illegible]	[illegible]	[illegible]	[illegible]	[illegible]	[illegible]	[illegible]	[illegible]	[illegible]	[illegible]	[illegible]	[illegible]
2014	Tajikistan	2919150	1220304	7109500	[illegible]	[illegible]	[illegible]	[illegible]	[illegible]	[illegible]	[illegible]	[illegible]	[illegible]	[illegible]	[illegible]	[illegible]	[illegible]	[illegible]	[illegible]	[illegible]	[illegible]	[illegible]	[illegible]	[illegible]	[illegible]	[illegible]	[illegible]	[illegible]
2014	Turkmenistan	2016231	712505	6080500	[illegible]	[illegible]	[illegible]	[illegible]	[illegible]	[illegible]	[illegible]	[illegible]	[illegible]	[illegible]	[illegible]	[illegible]	[illegible]	[illegible]	[illegible]	[illegible]	[illegible]	[illegible]	[illegible]	[illegible]	[illegible]	[illegible]	[illegible]	[illegible]
2019	Timor-Leste	425155	145493	497000	[illegible]	[illegible]	[illegible]	[illegible]	[illegible]	[illegible]	[illegible]	[illegible]	[illegible]	[illegible]	[illegible]	[illegible]	[illegible]	[illegible]	[illegible]	[illegible]	[illegible]	[illegible]	[illegible]	[illegible]	[illegible]	[illegible]	[illegible]	[illegible]
2013	Tonga	37986	14277	27500	[illegible]	[illegible]	[illegible]	[illegible]	[illegible]	[illegible]	[illegible]	[illegible]	[illegible]	[illegible]	[illegible]	[illegible]	[illegible]	[illegible]	[illegible]	[illegible]	[illegible]	[illegible]	[illegible]	[illegible]	[illegible]	[illegible]	[illegible]	[illegible]
2019	Trinidad and Tobago	807051	353705	3122000	[illegible]	[illegible]	[illegible]	[illegible]	[illegible]	[illegible]	[illegible]	[illegible]	[illegible]	[illegible]	[illegible]	[illegible]	[illegible]	[illegible]	[illegible]	[illegible]	[illegible]	[illegible]	[illegible]	[illegible]	[illegible]	[illegible]	[illegible]	[illegible]
2017	Tunisia	5967321	2042808	7446000	[illegible]	[illegible]	[illegible]	[illegible]	[illegible]	[illegible]	[illegible]	[illegible]	[illegible]	[illegible]	[illegible]	[illegible]	[illegible]	[illegible]	[illegible]	[illegible]	[illegible]	[illegible]	[illegible]	[illegible]	[illegible]	[illegible]	[illegible]	[illegible]
2018	Turkey	42850967	14040157	74375500	[illegible]	[illegible]	[illegible]	[illegible]	[illegible]	[illegible]	[illegible]	[illegible]	[illegible]	[illegible]	[illegible]	[illegible]	[illegible]	[illegible]	[illegible]	[illegible]	[illegible]	[illegible]	[illegible]	[illegible]	[illegible]	[illegible]	[illegible]	[illegible]
2014	Tuvalu	4664	2210	5000	[illegible]	[illegible]	[illegible]	[illegible]	[illegible]	[illegible]	[illegible]	[illegible]	[illegible]	[illegible]	[illegible]	[illegible]	[illegible]	[illegible]	[illegible]	[illegible]	[illegible]	[illegible]	[illegible]	[illegible]	[illegible]	[illegible]	[illegible]	[illegible]
2014	Tanzania	13920099	4405921	1507500	[illegible]	[illegible]	[illegible]	[illegible]	[illegible]	[illegible]	[illegible]	[illegible]	[illegible]	[illegible]	[illegible]	[illegible]	[illegible]	[illegible]	[illegible]	[illegible]	[illegible]	[illegible]	[illegible]	[illegible]	[illegible]	[illegible]	[illegible]	[illegible]
2017	Uganda	9344885	2755632	3459000	[illegible]	[illegible]	[illegible]	[illegible]	[illegible]	[illegible]	[illegible]	[illegible]	[illegible]	[illegible]	[illegible]	[illegible]	[illegible]	[illegible]	[illegible]	[illegible]	[illegible]	[illegible]	[illegible]	[illegible]	[illegible]	[illegible]	[illegible]	[illegible]
2014	Ukraine	28253557	14332666	67493000	[illegible]	[illegible]	[illegible]	[illegible]	[illegible]	[illegible]	[illegible]	[illegible]	[illegible]	[illegible]	[illegible]	[illegible]	[illegible]	[illegible]	[illegible]	[illegible]	[illegible]	[illegible]	[illegible]	[illegible]	[illegible]	[illegible]	[illegible]	[illegible]
2017	Uruguay	1819630	842612	8488000	[illegible]	[illegible]	[illegible]	[illegible]	[illegible]	[illegible]	[illegible]	[illegible]	[illegible]	[illegible]	[illegible]	[illegible]	[illegible]	[illegible]	[illegible]	[illegible]	[illegible]	[illegible]	[illegible]	[illegible]	[illegible]	[illegible]	[illegible]	[illegible]
2018	United States of America	187060457	68718647	425820500	[illegible]	[illegible]	[illegible]	[illegible]	[illegible]	[illegible]	[illegible]	[illegible]	[illegible]	[illegible]	[illegible]	[illegible]	[illegible]	[illegible]	[illegible]	[illegible]	[illegible]	[illegible]	[illegible]	[illegible]	[illegible]	[illegible]	[illegible]	[illegible]
2014	Uzbekistan	12340949	4833727	36118500	[illegible]	[illegible]	[illegible]	[illegible]	[illegible]	[illegible]	[illegible]	[illegible]	[illegible]	[illegible]	[illegible]	[illegible]	[illegible]	[illegible]	[illegible]	[illegible]	[illegible]	[illegible]	[illegible]	[illegible]	[illegible]	[illegible]	[illegible]	[illegible]
2012	Saint Vincent and the Grenadines	53991	21268	51000	[illegible]	[illegible]	[illegible]	[illegible]	[illegible]	[illegible]	[illegible]	[illegible]	[illegible]	[illegible]	[illegible]	[illegible]	[illegible]	[illegible]	[illegible]	[illegible]	[illegible]	[illegible]	[illegible]	[illegible]	[illegible]	[illegible]	[illegible]	[illegible]
2017	Venezuela	13768254	5343627	25433000	[illegible]	[illegible]	[illegible]	[illegible]	[illegible]	[illegible]	[illegible]	[illegible]	[illegible]	[illegible]	[illegible]	[illegible]	[illegible]	[illegible]	[illegible]	[illegible]	[illegible]	[illegible]	[illegible]	[illegible]	[illegible]	[illegible]	[illegible]	[illegible]
2016	Viet Nam	47137148	13045146	38769500	[illegible]	[illegible]	[illegible]	[illegible]	[illegible]	[illegible]	[illegible]	[illegible]	[illegible]	[illegible]	[illegible]	[illegible]	[illegible]	[illegible]	[illegible]	[illegible]	[illegible]	[illegible]	[illegible]	[illegible]	[illegible]	[illegible]	[illegible]	[illegible]
2016	Vanuatu	95071	34021	23000	[illegible]	[illegible]	[illegible]	[illegible]	[illegible]	[illegible]	[illegible]	[illegible]	[illegible]	[illegible]	[illegible]	[illegible]	[illegible]	[illegible]	[illegible]	[illegible]	[illegible]	[illegible]	[illegible]	[illegible]	[illegible]	[illegible]	[illegible]	[illegible]
2016	Samoa	73870	26578	33500	[illegible]	[illegible]	[illegible]	[illegible]	[illegible]	[illegible]	[illegible]	[illegible]	[illegible]	[illegible]	[illegible]	[illegible]	[illegible]	[illegible]	[illegible]	[illegible]	[illegible]	[illegible]	[illegible]	[illegible]	[illegible]	[illegible]	[illegible]	[illegible]
2014	Yemen	8029239	2024532	6780000	[illegible]	[illegible]	[illegible]	[illegible]	[illegible]	[illegible]	[illegible]	[illegible]	[illegible]	[illegible]	[illegible]	[illegible]	[illegible]	[illegible]	[illegible]	[illegible]	[illegible]	[illegible]	[illegible]	[illegible]	[illegible]	[illegible]	[illegible]	[illegible]
2019	South Africa	25508950	10546285	23196500	[illegible]	[illegible]	[illegible]	[illegible]	[illegible]	[illegible]	[illegible]	[illegible]	[illegible]	[illegible]	[illegible]	[illegible]	[illegible]	[illegible]	[illegible]	[illegible]	[illegible]	[illegible]	[illegible]	[illegible]	[illegible]	[illegible]	[illegible]	[illegible]
2016	Zambia	4466817	1271945	757000	[illegible]	[illegible]	[illegible]	[illegible]	[illegible]	[illegible]	[illegible]	[illegible]	[illegible]	[illegible]	[illegible]	[illegible]	[illegible]	[illegible]	[illegible]	[illegible]	[illegible]	[illegible]	[illegible]	[illegible]	[illegible]	[illegible]	[illegible]	[illegible]
2018	Zimbabwe	4629411	1732587	1513000	[illegible]	[illegible]	[illegible]	[illegible]	[illegible]	[illegible]	[illegible]	[illegible]	[illegible]	[illegible]	[illegible]	[illegible]	[illegible]	[illegible]	[illegible]	[illegible]	[illegible]	[illegible]	[illegible]	[illegible]	[illegible]	[illegible]	[illegible]	[illegible]

For peer review only

SpatialDim'	Period	Sex	ParentLoca	FactValueN	Prevalence	Prevalence	Prevalence	Proportion (
AFG	2016	Men	Eastern Me	9842	########	########	########	########
AFG	2016	Women	Eastern Me	9842	########	########	########	########
AGO	2017	Men	Africa	6400	########	########	########	########
AGO	2017	Women	Africa	6400	########	########	########	########
ALB	2019	Men	Europe	4745	########	########	########	########
ALB	2019	Women	Europe	4745	########	########	########	########
AND	2015	Men	Europe	260	########	########	########	########
AND	2015	Women	Europe	260	########	########	########	########
ARE	2018	Men	Eastern Me	24345	########	########	########	########
ARE	2018	Women	Eastern Me	24345	########	########	########	########
ARG	2017	Men	Americas	175313	########	########	########	########
ARG	2017	Women	Americas	175313	########	########	########	########
ARM	2017	Men	Europe	12964	########	########	########	########
ARM	2017	Women	Europe	12964	########	########	########	########
ATG	2017	Men	Americas	264	########	########	########	########
ATG	2017	Women	Americas	264	########	########	########	########
AUS	2018	Men	Western Pa	93604	########	########	########	########
AUS	2018	Women	Western Pa	93604	########	########	########	########
AUT	2018	Men	Europe	46337	########	########	########	########
AUT	2018	Women	Europe	46337	########	########	########	########
AZE	2014	Men	Europe	32756	########	########	########	########
AZE	2014	Women	Europe	32756	########	########	########	########
BDI	2017	Men	Africa	1084	########	########	########	########
BDI	2017	Women	Africa	1084	########	########	########	########
BEL	2019	Men	Europe	68724	########	########	########	########
BEL	2019	Women	Europe	68724	########	########	########	########
BEN	2019	Men	Africa	763	########	########	########	########
BEN	2019	Women	Africa	763	########	########	########	########
BFA	2019	Men	Africa	1910	########	########	########	########
BFA	2019	Women	Africa	1910	########	########	########	########
BGD	2019	Men	South-East	103809	########	########	########	########
BGD	2019	Women	South-East	103809	########	########	########	########
BGR	2018	Men	Europe	29667	########	########	########	########
BGR	2018	Women	Europe	29667	########	########	########	########
BHR	2015	Men	Eastern Me	1270	########	########	########	########
BHR	2015	Women	Eastern Me	1270	########	########	########	########
BHS	2017	Men	Americas	740	########	########	########	########
BHS	2017	Women	Americas	740	########	########	########	########
BIH	2015	Men	Europe	7413	########	########	########	########
BIH	2015	Women	Europe	7413	########	########	########	########
BLR	2015	Men	Europe	48995	########	########	########	########
BLR	2015	Women	Europe	48995	########	########	########	########
BLZ	2018	Men	Americas	413	########	########	########	########
BLZ	2018	Women	Americas	413	########	########	########	########
BOL	2017	Men	Americas	11528	########	########	########	########
BOL	2017	Women	Americas	11528	########	########	########	########
BRA	2019	Men	Americas	487659	########	########	########	########
BRA	2019	Women	Americas	487659	########	########	########	########
BRB	2017	Men	Americas	712	########	########	########	########
BRB	2017	Women	Americas	712	########	########	########	########
BRN	2017	Men	Western Pa	683	########	########	########	########
BRN	2017	Women	Western Pa	683	########	########	########	########
BTN	2019	Men	South-East	351	########	########	########	########
BTN	2019	Women	South-East	351	########	########	########	########
BWA	2018	Men	Africa	652	########	########	########	########

BWA	2018	Women	Africa	652	########	########	########	########
CAF	2015	Men	Africa	324	########	########	########	########
CAF	2015	Women	Africa	324	########	########	########	########
CAN	2019	Men	Americas	91375	########	########	########	########
CAN	2019	Women	Americas	91375	########	########	########	########
CHE	2018	Men	Europe	36940	########	########	########	########
CHE	2018	Women	Europe	36940	########	########	########	########
CHL	2018	Men	Americas	97062	########	########	########	########
CHL	2018	Women	Americas	97062	########	########	########	########
CHN	2017	Men	Western Pa	2828999	########	########	########	########
CHN	2017	Women	Western Pa	2828999	########	########	########	########
CIV	2019	Men	Africa	4173	########	########	########	########
CIV	2019	Women	Africa	4173	########	########	########	########
CMR	2011	Men	Africa	1842	########	########	########	########
CMR	2011	Women	Africa	1842	########	########	########	########
COD	2013	Men	Africa	6418	########	########	########	########
COD	2013	Women	Africa	6418	########	########	########	########
COG	2016	Men	Africa	530	########	########	########	########
COG	2016	Women	Africa	530	########	########	########	########
COK	2014	Men	Western Pa	25	########	########	########	########
COK	2014	Women	Western Pa	25	########	########	########	########
COL	2018	Men	Americas	190904	########	########	########	########
COL	2018	Women	Americas	190904	########	########	########	########
COM	2012	Men	Africa	123	########	########	########	########
COM	2012	Women	Africa	123	########	########	########	########
CPV	2015	Men	Africa	410	########	########	########	########
CPV	2015	Women	Africa	410	########	########	########	########
CRI	2018	Men	Americas	14468	########	########	########	########
CRI	2018	Women	Americas	14468	########	########	########	########
CUB	2018	Men	Americas	95466	########	########	########	########
CUB	2018	Women	Americas	95466	########	########	########	########
CYP	2016	Men	Europe	2283	########	########	########	########
CYP	2016	Women	Europe	2283	########	########	########	########
CZE	2019	Men	Europe	43994	########	########	########	########
CZE	2019	Women	Europe	43994	########	########	########	########
DEU	2018	Men	Europe	357401	########	########	########	########
DEU	2018	Women	Europe	357401	########	########	########	########
DJI	2014	Men	Eastern Me	201	########	########	########	########
DJI	2014	Women	Eastern Me	201	########	########	########	########
DMA	2018	Men	Americas	79	########	########	########	########
DMA	2018	Women	Americas	79	########	########	########	########
DNK	2018	Men	Europe	24301	########	########	########	########
DNK	2018	Women	Europe	24301	########	########	########	########
DOM	2019	Men	Americas	15591	########	########	########	########
DOM	2019	Women	Americas	15591	########	########	########	########
DZA	2018	Men	Africa	72604	########	########	########	########
DZA	2018	Women	Africa	72604	########	########	########	########
ECU	2017	Men	Americas	37263	########	########	########	########
ECU	2017	Women	Americas	37263	########	########	########	########
EGY	2019	Men	Eastern Me	74923	########	########	########	########
EGY	2019	Women	Eastern Me	74923	########	########	########	########
ERI	2004	Men	Africa	215	########	########	########	########
ERI	2004	Women	Africa	215	########	########	########	########
ESP	2018	Men	Europe	188166	########	########	########	########
ESP	2018	Women	Europe	188166	########	########	########	########
EST	2017	Men	Europe	4569	########	########	########	########

For peer review only

EST	2017	Women	Europe	4569	########	########	########	########
ETH	2018	Men	Africa	8395	########	########	########	########
ETH	2018	Women	Africa	8395	########	########	########	########
FIN	2018	Men	Europe	25627	########	########	########	########
FIN	2018	Women	Europe	25627	########	########	########	########
FJI	2015	Men	Western Pa	747	########	########	########	########
FJI	2015	Women	Western Pa	747	########	########	########	########
FRA	2018	Men	Europe	424674	########	########	########	########
FRA	2018	Women	Europe	424674	########	########	########	########
FSM	2009	Men	Western Pa	20	########	########	########	########
FSM	2009	Women	Western Pa	20	########	########	########	########
GAB	2017	Men	Africa	1408	########	########	########	########
GAB	2017	Women	Africa	1408	########	########	########	########
GBR	2019	Men	Europe	393247	########	########	########	########
GBR	2019	Women	Europe	393247	########	########	########	########
GEO	2019	Men	Europe	28291	########	########	########	########
GEO	2019	Women	Europe	28291	########	########	########	########
GHA	2019	Men	Africa	3236	########	########	########	########
GHA	2019	Women	Africa	3236	########	########	########	########
GIN	2016	Men	Africa	977	########	########	########	########
GIN	2016	Women	Africa	977	########	########	########	########
GMB	2019	Men	Africa	252	########	########	########	########
GMB	2019	Women	Africa	252	########	########	########	########
GNB	2016	Men	Africa	227	########	########	########	########
GNB	2016	Women	Africa	227	########	########	########	########
GNQ	2017	Men	Africa	507	########	########	########	########
GNQ	2017	Women	Africa	507	########	########	########	########
GRC	2018	Men	Europe	65513	########	########	########	########
GRC	2018	Women	Europe	65513	########	########	########	########
GRD	2018	Men	Americas	160	########	########	########	########
GRD	2018	Women	Americas	160	########	########	########	########
GTM	2018	Men	Americas	6122	########	########	########	########
GTM	2018	Women	Americas	6122	########	########	########	########
GUY	2018	Men	Americas	1417	########	########	########	########
GUY	2018	Women	Americas	1417	########	########	########	########
HND	2017	Men	Americas	2913	########	########	########	########
HND	2017	Women	Americas	2913	########	########	########	########
HRV	2016	Men	Europe	12624	########	########	########	########
HRV	2016	Women	Europe	12624	########	########	########	########
HTI	2018	Men	Americas	2606	########	########	########	########
HTI	2018	Women	Americas	2606	########	########	########	########
HUN	2018	Men	Europe	33078	########	########	########	########
HUN	2018	Women	Europe	33078	########	########	########	########
IDN	2019	Men	South-East	125862	########	########	########	########
IDN	2019	Women	South-East	125862	########	########	########	########
IND	2019	Men	South-East	1268172	########	########	########	########
IND	2019	Women	South-East	1268172	########	########	########	########
IRL	2019	Men	Europe	16366	########	########	########	########
IRL	2019	Women	Europe	16366	########	########	########	########
IRN	2018	Men	Eastern Me	129604	########	########	########	########
IRN	2018	Women	Eastern Me	129604	########	########	########	########
IRQ	2018	Men	Eastern Me	27208	########	########	########	########
IRQ	2018	Women	Eastern Me	27208	########	########	########	########
ISL	2019	Men	Europe	1404	########	########	########	########
ISL	2019	Women	Europe	1404	########	########	########	########
ISR	2019	Men	Europe	46573	########	########	########	########

For peer review only

ISR	2019	Women	Europe	46573	########	########	########	########
ITA	2019	Men	Europe	485190	########	########	########	########
ITA	2019	Women	Europe	485190	########	########	########	########
JAM	2016	Men	Americas	1322	########	########	########	########
JAM	2016	Women	Americas	1322	########	########	########	########
JOR	2017	Men	Eastern Me	22739	########	########	########	########
JOR	2017	Women	Eastern Me	22739	########	########	########	########
JPN	2018	Men	Western Pa	315406	########	########	########	########
JPN	2018	Women	Western Pa	315406	########	########	########	########
KAZ	2014	Men	Europe	68864	########	########	########	########
KAZ	2014	Women	Europe	68864	########	########	########	########
KEN	2018	Men	Africa	8042	########	########	########	########
KEN	2018	Women	Africa	8042	########	########	########	########
KGZ	2014	Men	Europe	12934	########	########	########	########
KGZ	2014	Women	Europe	12934	########	########	########	########
KHM	2014	Men	Western Pa	2944	########	########	########	########
KHM	2014	Women	Western Pa	2944	########	########	########	########
KIR	2013	Men	Western Pa	22	########	########	########	########
KIR	2013	Women	Western Pa	22	########	########	########	########
KNA	2018	Men	Americas	145	########	########	########	########
KNA	2018	Women	Americas	145	########	########	########	########
KOR	2018	Men	Western Pa	123230	########	########	########	########
KOR	2018	Women	Western Pa	123230	########	########	########	########
KWT	2015	Men	Eastern Me	10150	########	########	########	########
KWT	2015	Women	Eastern Me	10150	########	########	########	########
LAO	2017	Men	Western Pa	2591	########	########	########	########
LAO	2017	Women	Western Pa	2591	########	########	########	########
LBN	2018	Men	Eastern Me	14431	########	########	########	########
LBN	2018	Women	Eastern Me	14431	########	########	########	########
LBR	2015	Men	Africa	168	########	########	########	########
LBR	2015	Women	Africa	168	########	########	########	########
LBY	2017	Men	Eastern Me	13757	########	########	########	########
LBY	2017	Women	Eastern Me	13757	########	########	########	########
LCA	2017	Men	Americas	116	########	########	########	########
LCA	2017	Women	Americas	116	########	########	########	########
LKA	2019	Men	South-East	24605	########	########	########	########
LKA	2019	Women	South-East	24605	########	########	########	########
LSO	2010	Men	Africa	138	########	########	########	########
LSO	2010	Women	Africa	138	########	########	########	########
LTU	2019	Men	Europe	13908	########	########	########	########
LTU	2019	Women	Europe	13908	########	########	########	########
LUX	2017	Men	Europe	1780	########	########	########	########
LUX	2017	Women	Europe	1780	########	########	########	########
LVA	2018	Men	Europe	6367	########	########	########	########
LVA	2018	Women	Europe	6367	########	########	########	########
MAR	2017	Men	Eastern Me	26003	########	########	########	########
MAR	2017	Women	Eastern Me	26003	########	########	########	########
MDA	2019	Men	Europe	10345	########	########	########	########
MDA	2019	Women	Europe	10345	########	########	########	########
MDG	2014	Men	Africa	4275	########	########	########	########
MDG	2014	Women	Africa	4275	########	########	########	########
MDV	2018	Men	South-East	881	########	########	########	########
MDV	2018	Women	South-East	881	########	########	########	########
MEX	2018	Men	Americas	611970	########	########	########	########
MEX	2018	Women	Americas	611970	########	########	########	########
MHL	2012	Men	Western Pa	24	########	########	########	########

For peer review only

MHL	2012	Women	Western Pa	24	########	########	########	########
MKD	2015	Men	Europe	5975	########	########	########	########
MKD	2015	Women	Europe	5975	########	########	########	########
MLI	2018	Men	Africa	2454	########	########	########	########
MLI	2018	Women	Africa	2454	########	########	########	########
MLT	2015	Men	Europe	1240	########	########	########	########
MLT	2015	Women	Europe	1240	########	########	########	########
MMR	2019	Men	South-East	39826	########	########	########	########
MMR	2019	Women	South-East	39826	########	########	########	########
MNE	2018	Men	Europe	1730	########	########	########	########
MNE	2018	Women	Europe	1730	########	########	########	########
MNG	2018	Men	Western Pa	12211	########	########	########	########
MNG	2018	Women	Western Pa	12211	########	########	########	########
MOZ	2019	Men	Africa	2570	########	########	########	########
MOZ	2019	Women	Africa	2570	########	########	########	########
MRT	2018	Men	Africa	821	########	########	########	########
MRT	2018	Women	Africa	821	########	########	########	########
MUS	2018	Men	Africa	3210	########	########	########	########
MUS	2018	Women	Africa	3210	########	########	########	########
MWI	2018	Men	Africa	649	########	########	########	########
MWI	2018	Women	Africa	649	########	########	########	########
MYS	2015	Men	Western Pa	46491	########	########	########	########
MYS	2015	Women	Western Pa	46491	########	########	########	########
NAM	2017	Men	Africa	1421	########	########	########	########
NAM	2017	Women	Africa	1421	########	########	########	########
NER	2016	Men	Africa	900	########	########	########	########
NER	2016	Women	Africa	900	########	########	########	########
NGA	2018	Men	Africa	74543	########	########	########	########
NGA	2018	Women	Africa	74543	########	########	########	########
NIC	2018	Men	Americas	10762	########	########	########	########
NIC	2018	Women	Americas	10762	########	########	########	########
NIU	2008	Men	Western Pa	3	########	########	########	########
NIU	2008	Women	Western Pa	3	########	########	########	########
NLD	2018	Men	Europe	63233	########	########	########	########
NLD	2018	Women	Europe	63233	########	########	########	########
NOR	2019	Men	Europe	26276	########	########	########	########
NOR	2019	Women	Europe	26276	########	########	########	########
NPL	2019	Men	South-East	23146	########	########	########	########
NPL	2019	Women	South-East	23146	########	########	########	########
NRU	2015	Men	Western Pa	14	########	########	########	########
NRU	2015	Women	Western Pa	14	########	########	########	########
NZL	2018	Men	Western Pa	16224	########	########	########	########
NZL	2018	Women	Western Pa	16224	########	########	########	########
OMN	2019	Men	Eastern Me	9602	########	########	########	########
OMN	2019	Women	Eastern Me	9602	########	########	########	########
PAK	2019	Men	Eastern Me	242099	########	########	########	########
PAK	2019	Women	Eastern Me	242099	########	########	########	########
PAN	2019	Men	Americas	6932	########	########	########	########
PAN	2019	Women	Americas	6932	########	########	########	########
PER	2018	Men	Americas	26231	########	########	########	########
PER	2018	Women	Americas	26231	########	########	########	########
PHL	2017	Men	Western Pa	63141	########	########	########	########
PHL	2017	Women	Western Pa	63141	########	########	########	########
PLW	2014	Men	Western Pa	25	########	########	########	########
PLW	2014	Women	Western Pa	25	########	########	########	########
PNG	2019	Men	Western Pa	580	########	########	########	########

For peer review only

PNG	2019	Women	Western Pa	580	########	########	########	########
POL	2017	Men	Europe	90284	########	########	########	########
POL	2017	Women	Europe	90284	########	########	########	########
PRK	2017	Men	South-East	93667	########	########	########	########
PRK	2017	Women	South-East	93667	########	########	########	########
PRT	2018	Men	Europe	54478	########	########	########	########
PRT	2018	Women	Europe	54478	########	########	########	########
PRY	2018	Men	Americas	9421	########	########	########	########
PRY	2018	Women	Americas	9421	########	########	########	########
QAT	2018	Men	Eastern Me	6913	########	########	########	########
QAT	2018	Women	Eastern Me	6913	########	########	########	########
ROU	2017	Men	Europe	58583	########	########	########	########
ROU	2017	Women	Europe	58583	########	########	########	########
RUS	2018	Men	Europe	646993	########	########	########	########
RUS	2018	Women	Europe	646993	########	########	########	########
RWA	2019	Men	Africa	1492	########	########	########	########
RWA	2019	Women	Africa	1492	########	########	########	########
SAU	2018	Men	Eastern Me	88023	########	########	########	########
SAU	2018	Women	Eastern Me	88023	########	########	########	########
SDN	2017	Men	Eastern Me	10683	########	########	########	########
SDN	2017	Women	Eastern Me	10683	########	########	########	########
SEN	2019	Men	Africa	1435	########	########	########	########
SEN	2019	Women	Africa	1435	########	########	########	########
SGP	2016	Men	Western Pa	12967	########	########	########	########
SGP	2016	Women	Western Pa	12967	########	########	########	########
SLB	2016	Men	Western Pa	120	########	########	########	########
SLB	2016	Women	Western Pa	120	########	########	########	########
SLE	2018	Men	Africa	566	########	########	########	########
SLE	2018	Women	Africa	566	########	########	########	########
SLV	2018	Men	Americas	18427	########	########	########	########
SLV	2018	Women	Americas	18427	########	########	########	########
SOM	2014	Men	Eastern Me	309	########	########	########	########
SOM	2014	Women	Eastern Me	309	########	########	########	########
SRB	2016	Men	Europe	27563	########	########	########	########
SRB	2016	Women	Europe	27563	########	########	########	########
STP	2015	Men	Africa	63	########	########	########	########
STP	2015	Women	Africa	63	########	########	########	########
SUR	2018	Men	Americas	472	########	########	########	########
SUR	2018	Women	Americas	472	########	########	########	########
SVK	2018	Men	Europe	19178	########	########	########	########
SVK	2018	Women	Europe	19178	########	########	########	########
SVN	2018	Men	Europe	6591	########	########	########	########
SVN	2018	Women	Europe	6591	########	########	########	########
SWE	2017	Men	Europe	42898	########	########	########	########
SWE	2017	Women	Europe	42898	########	########	########	########
SWZ	2016	Men	Africa	107	########	########	########	########
SWZ	2016	Women	Africa	107	########	########	########	########
SYC	2018	Men	Africa	240	########	########	########	########
SYC	2018	Women	Africa	240	########	########	########	########
SYR	2016	Men	Eastern Me	22485	########	########	########	########
SYR	2016	Women	Eastern Me	22485	########	########	########	########
TCD	2019	Men	Africa	865	########	########	########	########
TCD	2019	Women	Africa	865	########	########	########	########
TGO	2019	Men	Africa	627	########	########	########	########
TGO	2019	Women	Africa	627	########	########	########	########
THA	2019	Men	South-East	63974	########	########	########	########

For peer review only

THA	2019	Women	South-East	63974	########	########	########	########
TJK	2014	Men	Europe	14219	########	########	########	########
TJK	2014	Women	Europe	14219	########	########	########	########
TKM	2014	Men	Europe	12161	########	########	########	########
TKM	2014	Women	Europe	12161	########	########	########	########
TLS	2019	Men	South-East	994	########	########	########	########
TLS	2019	Women	South-East	994	########	########	########	########
TON	2013	Men	Western Pa	55	########	########	########	########
TON	2013	Women	Western Pa	55	########	########	########	########
TTO	2019	Men	Americas	6244	########	########	########	########
TTO	2019	Women	Americas	6244	########	########	########	########
TUN	2017	Men	Eastern Me	14892	########	########	########	########
TUN	2017	Women	Eastern Me	14892	########	########	########	########
TUR	2018	Men	Europe	148751	########	########	########	########
TUR	2018	Women	Europe	148751	########	########	########	########
TUV	2014	Men	Western Pa	10	########	########	########	########
TUV	2014	Women	Western Pa	10	########	########	########	########
TZA	2014	Men	Africa	3015	########	########	########	########
TZA	2014	Women	Africa	3015	########	########	########	########
UGA	2017	Men	Africa	6918	########	########	########	########
UGA	2017	Women	Africa	6918	########	########	########	########
UKR	2014	Men	Europe	134986	########	########	########	########
UKR	2014	Women	Europe	134986	########	########	########	########
URY	2017	Men	Americas	16976	########	########	########	########
URY	2017	Women	Americas	16976	########	########	########	########
USA	2018	Men	Americas	851641	########	########	########	########
USA	2018	Women	Americas	851641	########	########	########	########
UZB	2014	Men	Europe	72237	########	########	########	########
UZB	2014	Women	Europe	72237	########	########	########	########
VCT	2012	Men	Americas	102	########	########	########	########
VCT	2012	Women	Americas	102	########	########	########	########
VEN	2017	Men	Americas	50866	########	########	########	########
VEN	2017	Women	Americas	50866	########	########	########	########
VNM	2016	Men	Western Pa	77539	########	########	########	########
VNM	2016	Women	Western Pa	77539	########	########	########	########
VUT	2016	Men	Western Pa	46	########	########	########	########
VUT	2016	Women	Western Pa	46	########	########	########	########
WSM	2016	Men	Western Pa	67	########	########	########	########
WSM	2016	Women	Western Pa	67	########	########	########	########
YEM	2014	Men	Eastern Me	13560	########	########	########	########
YEM	2014	Women	Eastern Me	13560	########	########	########	########
ZAF	2019	Men	Africa	46393	########	########	########	########
ZAF	2019	Women	Africa	46393	########	########	########	########
ZMB	2016	Men	Africa	1514	########	########	########	########
ZMB	2016	Women	Africa	1514	########	########	########	########
ZWE	2018	Men	Africa	3026	########	########	########	########
ZWE	2018	Women	Africa	3026	########	########	########	########

For peer review only

Proportion	Proportion	Proportion	Proportion	Proportion	Proportion	Proportion	Proportion	Proportion
########	########	########	########	########	########	########	########	########
########	########	########	########	########	########	########	########	########
########	########	########	########	########	########	########	########	########
########	########	########	########	########	########	########	########	########
########	########	########	########	########	########	########	########	########
########	########	########	########	########	########	########	########	########
########	########	########	########	########	########	########	########	########
########	########	########	########	########	########	########	########	########
########	########	########	########	########	########	########	########	########
########	########	########	########	########	########	########	########	########
########	########	########	########	########	########	########	########	########
########	########	########	########	########	########	########	########	########
########	########	########	########	########	########	########	########	########
########	########	########	########	########	########	########	########	########
########	########	########	########	########	########	########	########	########
########	########	########	########	########	########	########	########	########
########	########	########	########	########	########	########	########	########
########	########	########	########	########	########	########	########	########
########	########	########	########	########	########	########	########	########
########	########	########	########	########	########	########	########	########
########	########	########	########	########	########	########	########	########
########	########	########	########	########	########	########	########	########
########	########	########	########	########	########	########	########	########
########	########	########	########	########	########	########	########	########
########	########	########	########	########	########	########	########	########
########	########	########	########	########	########	########	########	########
########	########	########	########	########	########	########	########	########
########	########	########	########	########	########	########	########	########
########	########	########	########	########	########	########	########	########
########	########	########	########	########	########	########	########	########
########	########	########	########	########	########	########	########	########
########	########	########	########	########	########	########	########	########
########	########	########	########	########	########	########	########	########
########	########	########	########	########	########	########	########	########
########	########	########	########	########	########	########	########	########
########	########	########	########	########	########	########	########	########
########	########	########	########	########	########	########	########	########
########	########	########	########	########	########	########	########	########
########	########	########	########	########	########	########	########	########
########	########	########	########	########	########	########	########	########
########	########	########	########	########	########	########	########	########
########	########	########	########	########	########	########	########	########
########	########	########	########	########	########	########	########	########
########	########	########	########	########	########	########	########	########
########	########	########	########	########	########	########	########	########
########	########	########	########	########	########	########	########	########
########	########	########	########	########	########	########	########	########
########	########	########	########	########	########	########	########	########
########	########	########	########	########	########	########	########	########
########	########	########	########	########	########	########	########	########
########	########	########	########	########	########	########	########	########
########	########	########	########	########	########	########	########	########
########	########	########	########	########	########	########	########	########
########	########	########	########	########	########	########	########	########
########	########	########	########	########	########	########	########	########
########	########	########	########	########	########	########	########	########
########	########	########	########	########	########	########	########	########

For peer review only

########	########	########	########	########	########	########	########	########
########	########	########	########	########	########	########	########	########
########	########	########	########	########	########	########	########	########
########	########	########	########	########	########	########	########	########
########	########	########	########	########	########	########	########	########
########	########	########	########	########	########	########	########	########
########	########	########	########	########	########	########	########	########
########	########	########	########	########	########	########	########	########
########	########	########	########	########	########	########	########	########
########	########	########	########	########	########	########	########	########
########	########	########	########	########	########	########	########	########
########	########	########	########	########	########	########	########	########
########	########	########	########	########	########	########	########	########
########	########	########	########	########	########	########	########	########
########	########	########	########	########	########	########	########	########
########	########	########	########	########	########	########	########	########
########	########	########	########	########	########	########	########	########
########	########	########	########	########	########	########	########	########
########	########	########	########	########	########	########	########	########
########	########	########	########	########	########	########	########	########
########	########	########	########	########	########	########	########	########
########	########	########	########	########	########	########	########	########
########	########	########	########	########	########	########	########	########
########	########	########	########	########	########	########	########	########
########	########	########	########	########	########	########	########	########
########	########	########	########	########	########	########	########	########
########	########	########	########	########	########	########	########	########
########	########	########	########	########	########	########	########	########
########	########	########	########	########	########	########	########	########
########	########	########	########	########	########	########	########	########
########	########	########	########	########	########	########	########	########
########	########	########	########	########	########	########	########	########
########	########	########	########	########	########	########	########	########
########	########	########	########	########	########	########	########	########
########	########	########	########	########	########	########	########	########
########	########	########	########	########	########	########	########	########
########	########	########	########	########	########	########	########	########
########	########	########	########	########	########	########	########	########
########	########	########	########	########	########	########	########	########
########	########	########	########	########	########	########	########	########
########	########	########	########	########	########	########	########	########
########	########	########	########	########	########	########	########	########
########	########	########	########	########	########	########	########	########
########	########	########	########	########	########	########	########	########
########	########	########	########	########	########	########	########	########
########	########	########	########	########	########	########	########	########
########	########	########	########	########	########	########	########	########
########	########	########	########	########	########	########	########	########
########	########	########	########	########	########	########	########	########
########	########	########	########	########	########	########	########	########
########	########	########	########	########	########	########	########	########
########	########	########	########	########	########	########	########	########
########	########	########	########	########	########	########	########	########
########	########	########	########	########	########	########	########	########
########	########	########	########	########	########	########	########	########
########	########	########	########	########	########	########	########	########
########	########	########	########	########	########	########	########	########
########	########	########	########	########	########	########	########	########

For peer review only

########	########	########	########	########	########	########	########	########
########	########	########	########	########	########	########	########	########
########	########	########	########	########	########	########	########	########
########	########	########	########	########	########	########	########	########
########	########	########	########	########	########	########	########	########
########	########	########	########	########	########	########	########	########
########	########	########	########	########	########	########	########	########
########	########	########	########	########	########	########	########	########
########	########	########	########	########	########	########	########	########
########	########	########	########	########	########	########	########	########
########	########	########	########	########	########	########	########	########
########	########	########	########	########	########	########	########	########
########	########	########	########	########	########	########	########	########
########	########	########	########	########	########	########	########	########
########	########	########	########	########	########	########	########	########
########	########	########	########	########	########	########	########	########
########	########	########	########	########	########	########	########	########
########	########	########	########	########	########	########	########	########
########	########	########	########	########	########	########	########	########
########	########	########	########	########	########	########	########	########
########	########	########	########	########	########	########	########	########
########	########	########	########	########	########	########	########	########
########	########	########	########	########	########	########	########	########
########	########	########	########	########	########	########	########	########
########	########	########	########	########	########	########	########	########
########	########	########	########	########	########	########	########	########
########	########	########	########	########	########	########	########	########
########	########	########	########	########	########	########	########	########
########	########	########	########	########	########	########	########	########
########	########	########	########	########	########	########	########	########
########	########	########	########	########	########	########	########	########
########	########	########	########	########	########	########	########	########
########	########	########	########	########	########	########	########	########
########	########	########	########	########	########	########	########	########
########	########	########	########	########	########	########	########	########
########	########	########	########	########	########	########	########	########
########	########	########	########	########	########	########	########	########
########	########	########	########	########	########	########	########	########
########	########	########	########	########	########	########	########	########
########	########	########	########	########	########	########	########	########
########	########	########	########	########	########	########	########	########
########	########	########	########	########	########	########	########	########
########	########	########	########	########	########	########	########	########
########	########	########	########	########	########	########	########	########
########	########	########	########	########	########	########	########	########
########	########	########	########	########	########	########	########	########
########	########	########	########	########	########	########	########	########
########	########	########	########	########	########	########	########	########
########	########	########	########	########	########	########	########	########
########	########	########	########	########	########	########	########	########
########	########	########	########	########	########	########	########	########
########	########	########	########	########	########	########	########	########
########	########	########	########	########	########	########	########	########
########	########	########	########	########	########	########	########	########
########	########	########	########	########	########	########	########	########
########	########	########	########	########	########	########	########	########
########	########	########	########	########	########	########	########	########
########	########	########	########	########	########	########	########	########

For peer review only

########	########	########	########	########	########	########	########	########
########	########	########	########	########	########	########	########	########
########	########	########	########	########	########	########	########	########
########	########	########	########	########	########	########	########	########
########	########	########	########	########	########	########	########	########
########	########	########	########	########	########	########	########	########
########	########	########	########	########	########	########	########	########
########	########	########	########	########	########	########	########	########
########	########	########	########	########	########	########	########	########
########	########	########	########	########	########	########	########	########
########	########	########	########	########	########	########	########	########
########	########	########	########	########	########	########	########	########
########	########	########	########	########	########	########	########	########
########	########	########	########	########	########	########	########	########
########	########	########	########	########	########	########	########	########
########	########	########	########	########	########	########	########	########
########	########	########	########	########	########	########	########	########
########	########	########	########	########	########	########	########	########
########	########	########	########	########	########	########	########	########
########	########	########	########	########	########	########	########	########
########	########	########	########	########	########	########	########	########
########	########	########	########	########	########	########	########	########
########	########	########	########	########	########	########	########	########
########	########	########	########	########	########	########	########	########
########	########	########	########	########	########	########	########	########
########	########	########	########	########	########	########	########	########
########	########	########	########	########	########	########	########	########
########	########	########	########	########	########	########	########	########
########	########	########	########	########	########	########	########	########
########	########	########	########	########	########	########	########	########
########	########	########	########	########	########	########	########	########
########	########	########	########	########	########	########	########	########
########	########	########	########	########	########	########	########	########
########	########	########	########	########	########	########	########	########
########	########	########	########	########	########	########	########	########
########	########	########	########	########	########	########	########	########
########	########	########	########	########	########	########	########	########
########	########	########	########	########	########	########	########	########
########	########	########	########	########	########	########	########	########
########	########	########	########	########	########	########	########	########
########	########	########	########	########	########	########	########	########
########	########	########	########	########	########	########	########	########
########	########	########	########	########	########	########	########	########
########	########	########	########	########	########	########	########	########
########	########	########	########	########	########	########	########	########
########	########	########	########	########	########	########	########	########
########	########	########	########	########	########	########	########	########
########	########	########	########	########	########	########	########	########
########	########	########	########	########	########	########	########	########
########	########	########	########	########	########	########	########	########
########	########	########	########	########	########	########	########	########
########	########	########	########	########	########	########	########	########
########	########	########	########	########	########	########	########	########
########	########	########	########	########	########	########	########	########
########	########	########	########	########	########	########	########	########
########	########	########	########	########	########	########	########	########
########	########	########	########	########	########	########	########	########
########	########	########	########	########	########	########	########	########

For peer review only

########	########	########	########	########	########	########	########	########
########	########	########	########	########	########	########	########	########
########	########	########	########	########	########	########	########	########
########	########	########	########	########	########	########	########	########
########	########	########	########	########	########	########	########	########
########	########	########	########	########	########	########	########	########
########	########	########	########	########	########	########	########	########
########	########	########	########	########	########	########	########	########
########	########	########	########	########	########	########	########	########
########	########	########	########	########	########	########	########	########
########	########	########	########	########	########	########	########	########
########	########	########	########	########	########	########	########	########
########	########	########	########	########	########	########	########	########
########	########	########	########	########	########	########	########	########
########	########	########	########	########	########	########	########	########
########	########	########	########	########	########	########	########	########
########	########	########	########	########	########	########	########	########
########	########	########	########	########	########	########	########	########
########	########	########	########	########	########	########	########	########
########	########	########	########	########	########	########	########	########
########	########	########	########	########	########	########	########	########
########	########	########	########	########	########	########	########	########
########	########	########	########	########	########	########	########	########
########	########	########	########	########	########	########	########	########
########	########	########	########	########	########	########	########	########
########	########	########	########	########	########	########	########	########
########	########	########	########	########	########	########	########	########
########	########	########	########	########	########	########	########	########
########	########	########	########	########	########	########	########	########
########	########	########	########	########	########	########	########	########
########	########	########	########	########	########	########	########	########
########	########	########	########	########	########	########	########	########
########	########	########	########	########	########	########	########	########
########	########	########	########	########	########	########	########	########
########	########	########	########	########	########	########	########	########
########	########	########	########	########	########	########	########	########
########	########	########	########	########	########	########	########	########
########	########	########	########	########	########	########	########	########
########	########	########	########	########	########	########	########	########
########	########	########	########	########	########	########	########	########
########	########	########	########	########	########	########	########	########
########	########	########	########	########	########	########	########	########
########	########	########	########	########	########	########	########	########
########	########	########	########	########	########	########	########	########
########	########	########	########	########	########	########	########	########
########	########	########	########	########	########	########	########	########
########	########	########	########	########	########	########	########	########
########	########	########	########	########	########	########	########	########
########	########	########	########	########	########	########	########	########
########	########	########	########	########	########	########	########	########
########	########	########	########	########	########	########	########	########
########	########	########	########	########	########	########	########	########
########	########	########	########	########	########	########	########	########
########	########	########	########	########	########	########	########	########
########	########	########	########	########	########	########	########	########
########	########	########	########	########	########	########	########	########
########	########	########	########	########	########	########	########	########
########	########	########	########	########	########	########	########	########

For peer review only

########	########	########	########	########	########	########	########	########
########	########	########	########	########	########	########	########	########
########	########	########	########	########	########	########	########	########
########	########	########	########	########	########	########	########	########
########	########	########	########	########	########	########	########	########
########	########	########	########	########	########	########	########	########
########	########	########	########	########	########	########	########	########
########	########	########	########	########	########	########	########	########
########	########	########	########	########	########	########	########	########
########	########	########	########	########	########	########	########	########
########	########	########	########	########	########	########	########	########
########	########	########	########	########	########	########	########	########
########	########	########	########	########	########	########	########	########
########	########	########	########	########	########	########	########	########
########	########	########	########	########	########	########	########	########
########	########	########	########	########	########	########	########	########
########	########	########	########	########	########	########	########	########
########	########	########	########	########	########	########	########	########
########	########	########	########	########	########	########	########	########
########	########	########	########	########	########	########	########	########
########	########	########	########	########	########	########	########	########
########	########	########	########	########	########	########	########	########
########	########	########	########	########	########	########	########	########
########	########	########	########	########	########	########	########	########
########	########	########	########	########	########	########	########	########
########	########	########	########	########	########	########	########	########
########	########	########	########	########	########	########	########	########
########	########	########	########	########	########	########	########	########
########	########	########	########	########	########	########	########	########
########	########	########	########	########	########	########	########	########
########	########	########	########	########	########	########	########	########
########	########	########	########	########	########	########	########	########
########	########	########	########	########	########	########	########	########
########	########	########	########	########	########	########	########	########
########	########	########	########	########	########	########	########	########
########	########	########	########	########	########	########	########	########
########	########	########	########	########	########	########	########	########
########	########	########	########	########	########	########	########	########
########	########	########	########	########	########	########	########	########
########	########	########	########	########	########	########	########	########
########	########	########	########	########	########	########	########	########
########	########	########	########	########	########	########	########	########
########	########	########	########	########	########	########	########	########
########	########	########	########	########	########	########	########	########
########	########	########	########	########	########	########	########	########
########	########	########	########	########	########	########	########	########
########	########	########	########	########	########	########	########	########
########	########	########	########	########	########	########	########	########
########	########	########	########	########	########	########	########	########
########	########	########	########	########	########	########	########	########
########	########	########	########	########	########	########	########	########
########	########	########	########	########	########	########	########	########
########	########	########	########	########	########	########	########	########
########	########	########	########	########	########	########	########	########
########	########	########	########	########	########	########	########	########
########	########	########	########	########	########	########	########	########
########	########	########	########	########	########	########	########	########
########	########	########	########	########	########	########	########	########

For peer review only

########	########	########	########	########	########	########	########	########
########	########	########	########	########	########	########	########	########
########	########	########	########	########	########	########	########	########
########	########	########	########	########	########	########	########	########
########	########	########	########	########	########	########	########	########
########	########	########	########	########	########	########	########	########
########	########	########	########	########	########	########	########	########
########	########	########	########	########	########	########	########	########
########	########	########	########	########	########	########	########	########
########	########	########	########	########	########	########	########	########
########	########	########	########	########	########	########	########	########
########	########	########	########	########	########	########	########	########
########	########	########	########	########	########	########	########	########
########	########	########	########	########	########	########	########	########
########	########	########	########	########	########	########	########	########
########	########	########	########	########	########	########	########	########
########	########	########	########	########	########	########	########	########
########	########	########	########	########	########	########	########	########
########	########	########	########	########	########	########	########	########
########	########	########	########	########	########	########	########	########
########	########	########	########	########	########	########	########	########
########	########	########	########	########	########	########	########	########
########	########	########	########	########	########	########	########	########
########	########	########	########	########	########	########	########	########
########	########	########	########	########	########	########	########	########
########	########	########	########	########	########	########	########	########
########	########	########	########	########	########	########	########	########
########	########	########	########	########	########	########	########	########
########	########	########	########	########	########	########	########	########
########	########	########	########	########	########	########	########	########
########	########	########	########	########	########	########	########	########
########	########	########	########	########	########	########	########	########
########	########	########	########	########	########	########	########	########
########	########	########	########	########	########	########	########	########
########	########	########	########	########	########	########	########	########
########	########	########	########	########	########	########	########	########
########	########	########	########	########	########	########	########	########
########	########	########	########	########	########	########	########	########
########	########	########	########	########	########	########	########	########
########	########	########	########	########	########	########	########	########
########	########	########	########	########	########	########	########	########
########	########	########	########	########	########	########	########	########
########	########	########	########	########	########	########	########	########
########	########	########	########	########	########	########	########	########
########	########	########	########	########	########	########	########	########
########	########	########	########	########	########	########	########	########
########	########	########	########	########	########	########	########	########

For peer review only

Proportion	Proportion	country	pop
########	########	Afghanistar	4374875
########	########	Afghanistar	4238918
########	########	Angola	3578447
########	########	Angola	4035463
########	########	Albania	753661
########	########	Albania	785847
########	########	Andorra	26121
########	########	Andorra	24913
########	########	United Aral	5358519
########	########	United Aral	1482314
########	########	Argentina	10472550
########	########	Argentina	11415917
########	########	Armenia	776633
########	########	Armenia	910316
########	########	Antigua and	22686
########	########	Antigua and	25059
########	########	Australia	6834112
########	########	Australia	7130677
########	########	Austria	2711593
########	########	Austria	2814924
########	########	Azerbaijan	2267064
########	########	Azerbaijan	2457603
########	########	Burundi	1579397
########	########	Burundi	1460878
########	########	Belgium	3362353
########	########	Belgium	3441544
########	########	Benin	1618415
########	########	Benin	1758315
########	########	Burkina Fas	2873406
########	########	Burkina Fas	3382165
########	########	Bangladesl	34397899
########	########	Bangladesl	35064009
########	########	Bulgaria	2226843
########	########	Bulgaria	2389075
########	########	Bahrain	579523
########	########	Bahrain	270039
########	########	Bahamas	91445
########	########	Bahamas	101257
########	########	Bosnia and	1053198
########	########	Bosnia and	1127363
########	########	Belarus	2631774
########	########	Belarus	3209374
########	########	Belize	78125
########	########	Belize	79466
########	########	Bolivia	2239567
########	########	Bolivia	2312282
########	########	Brazil	54239088
########	########	Brazil	59367580
########	########	Barbados	82705
########	########	Barbados	92721
########	########	Brunei Dar	110240
########	########	Brunei Dar	101524
########	########	Bhutan	173110
########	########	Bhutan	155950
########	########	Botswana	458457

For peer review only

########	########	Botswana	492383
########	########	Central Afri	698380
########	########	Central Afri	726015
########	########	Canada	10816059
########	########	Canada	11353591
########	########	Switzerlanc	2703056
########	########	Switzerlanc	2712187
########	########	Chile	4717435
########	########	Chile	5084271
########	########	China	4.37E+08
########	########	China	4.3E+08
########	########	Cote d'Ivoir	4361485
########	########	Cote d'Ivoir	3905875
########	########	Cameroon	3053294
########	########	Cameroon	3118458
########	########	DR Congo	9946640
########	########	DR Congo	10268403
########	########	Congo	859180
########	########	Congo	858844
########	########	Cook Islanc	4507
########	########	Cook Islanc	4733
########	########	Colombia	10925514
########	########	Colombia	12164733
########	########	Comoros	113347
########	########	Comoros	118362
########	########	Cabo Verde	103754
########	########	Cabo Verde	106368
########	########	Costa Rica	1111630
########	########	Costa Rica	1237275
########	########	Cuba	3404209
########	########	Cuba	3528783
########	########	Cyprus	360790
########	########	Cyprus	384743
########	########	Czech Rep	3400577
########	########	Czech Rep	3503214
########	########	Germany	26427152
########	########	Germany	26886690
########	########	Djibouti	200037
########	########	Djibouti	176624
########	########	Dominica	18375
########	########	Dominica	17329
########	########	Denmark	1706501
########	########	Denmark	1726551
########	########	Dominican	2396279
########	########	Dominican	2387143
########	########	Algeria	9630862
########	########	Algeria	9544497
########	########	Ecuador	3517434
########	########	Ecuador	3691903
########	########	Egypt	20701270
########	########	Egypt	18937856
########	########	Eritrea	607346
########	########	Eritrea	664917
########	########	Spain	14519891
########	########	Spain	15012788
########	########	Estonia	372864

For peer review only

########	########	Estonia	432706
########	########	Ethiopia	14098289
########	########	Ethiopia	14221567
########	########	Finland	1664873
########	########	Finland	1697833
########	########	Fiji	203408
########	########	Fiji	199509
########	########	France	18577439
########	########	France	19831021
########	########	Micronesia	18425
########	########	Micronesia	18700
########	########	Gabon	301687
########	########	Gabon	318342
########	########	United King	19573142
########	########	United King	20116673
########	########	Georgia	982269
########	########	Georgia	1138946
########	########	Ghana	5061012
########	########	Ghana	5848367
########	########	Guinea	1498509
########	########	Guinea	1666947
########	########	Gambia	315472
########	########	Gambia	329202
########	########	Guinea Bis	228996
########	########	Guinea Bis	262435
########	########	Equatorial (	161909
########	########	Equatorial (	182562
########	########	Greece	3194397
########	########	Greece	3394264
########	########	Grenada	26429
########	########	Grenada	25346
########	########	Guatemala	2769247
########	########	Guatemala	3334832
########	########	Guyana	159753
########	########	Guyana	169100
########	########	Honduras	1536610
########	########	Honduras	1743696
########	########	Croatia	1307436
########	########	Croatia	1405103
########	########	Haiti	2089510
########	########	Haiti	2387792
########	########	Hungary	2947365
########	########	Hungary	3308097
########	########	Indonesia	63617376
########	########	Indonesia	63411351
########	########	India	3.12E+08
########	########	India	3.05E+08
########	########	Ireland	1388743
########	########	Ireland	1443205
########	########	Iran	21709285
########	########	Iran	21362969
########	########	Iraq	7492486
########	########	Iraq	7204492
########	########	Iceland	97340
########	########	Iceland	94859
########	########	Israel	2169397

For peer review only

########	########	Israel	2263622
########	########	Italy	18975888
########	########	Italy	20012297
########	########	Jamaica	633038
########	########	Jamaica	658181
########	########	Jordan	2186597
########	########	Jordan	1846683
########	########	Japan	40765331
########	########	Japan	41831824
########	########	Kazakhstar	3773051
########	########	Kazakhstar	4380871
########	########	Kenya	7534093
########	########	Kenya	7755655
########	########	Kyrgyzstan	1111881
########	########	Kyrgyzstan	1221203
########	########	Cambodia	2696527
########	########	Cambodia	3165719
########	########	Kiribati	18297
########	########	Kiribati	20745
########	########	Saint Kitts a	16639
########	########	Saint Kitts a	16181
########	########	South Kore	17445980
########	########	South Kore	17183536
########	########	Kuwait	1193460
########	########	Kuwait	855872
########	########	Lao PDR	1309250
########	########	Lao PDR	1318939
########	########	Lebanon	1137874
########	########	Lebanon	1313603
########	########	Liberia	689971
########	########	Liberia	660850
########	########	Libya	1688759
########	########	Libya	1548933
########	########	Saint Lucia	46509
########	########	Saint Lucia	47627
########	########	Sri Lanka	5428978
########	########	Sri Lanka	6088794
########	########	Lesotho	309758
########	########	Lesotho	349051
########	########	Lithuania	777836
########	########	Lithuania	945506
########	########	Luxembour	180759
########	########	Luxembour	177166
########	########	Latvia	546675
########	########	Latvia	666165
########	########	Morocco	8099191
########	########	Morocco	8215262
########	########	Moldova	1078314
########	########	Moldova	1236158
########	########	Madagasca	3346902
########	########	Madagasca	3451462
########	########	Maldives	147468
########	########	Maldives	89653
########	########	Mexico	27449102
########	########	Mexico	30264955
########	########	Marshall Isl	10287

For peer review only

########	########	Marshall Isl	9737
########	########	Macedonia	643578
########	########	Macedonia	636410
########	########	Mali	2628361
########	########	Mali	2710558
########	########	Malta	134791
########	########	Malta	135299
########	########	Myanmar	11601347
########	########	Myanmar	13497890
########	########	Montenegro	178882
########	########	Montenegro	190548
########	########	Mongolia	738402
########	########	Mongolia	799515
########	########	Mozambiqu	3520747
########	########	Mozambiqu	4025421
########	########	Mauritania	585331
########	########	Mauritania	624447
########	########	Mauritius	364114
########	########	Mauritius	377299
########	########	Malawi	2346357
########	########	Malawi	2550782
########	########	Malaysia	6885827
########	########	Malaysia	6443338
########	########	Namibia	382255
########	########	Namibia	434271
########	########	Niger	2289168
########	########	Niger	2429825
########	########	Nigeria	27622842
########	########	Nigeria	31083880
########	########	Nicaragua	1207472
########	########	Nicaragua	1338743
########	########	Niue	396
########	########	Niue	400
########	########	Netherlands	5156417
########	########	Netherlands	5221884
########	########	Norway	1595625
########	########	Norway	1549624
########	########	Nepal	5460110
########	########	Nepal	6521161
########	########	Nauru	1689
########	########	Nauru	1762
########	########	New Zealar	1208077
########	########	New Zealar	1324726
########	########	Oman	1545994
########	########	Oman	675172
########	########	Pakistan	36843681
########	########	Pakistan	35594810
########	########	Panama	952915
########	########	Panama	957111
########	########	Peru	7448500
########	########	Peru	7847999
########	########	Philippines	21493672
########	########	Philippines	21503099
########	########	Palau	5450
########	########	Palau	4683
########	########	Papua New	1757711

For peer review only

########	########	Papua New	1672933
########	########	Poland	11624749
########	########	Poland	12527237
########	########	North Korea	7063065
########	########	North Korea	7330538
########	########	Portugal	3232847
########	########	Portugal	3619042
########	########	Paraguay	1445219
########	########	Paraguay	1433290
########	########	Qatar	1191956
########	########	Qatar	337887
########	########	Romania	5940958
########	########	Romania	6367663
########	########	Russian Fe	40775809
########	########	Russian Fe	49582955
########	########	Rwanda	1942367
########	########	Rwanda	2225522
########	########	Saudi Arab	11045939
########	########	Saudi Arab	6938293
########	########	Sudan	5985695
########	########	Sudan	6070204
########	########	Senegal	2231386
########	########	Senegal	2406392
########	########	Singapore	1898845
########	########	Singapore	1664785
########	########	Solomon Is	102928
########	########	Solomon Is	101046
########	########	Sierra Leor	1178706
########	########	Sierra Leor	1182441
########	########	El Salvador	1131627
########	########	El Salvador	1489017
########	########	Somalia	2135916
########	########	Somalia	2233729
########	########	Serbia	2611341
########	########	Serbia	2760624
########	########	Sao Tome	30801
########	########	Sao Tome	31088
########	########	Suriname	137105
########	########	Suriname	144772
########	########	Slovakia	1672651
########	########	Slovakia	1779076
########	########	Slovenia	672651
########	########	Slovenia	667111
########	########	Sweden	2957850
########	########	Sweden	2929908
########	########	Swaziland	165071
########	########	Swaziland	193141
########	########	Seychelles	29591
########	########	Seychelles	25893
########	########	Syrian Arab	3181953
########	########	Syrian Arab	3330300
########	########	Chad	1887442
########	########	Chad	1921465
########	########	Togo	1188662
########	########	Togo	1413001
########	########	Thailand	20948002

For peer review only

########	########	Thailand	22590236
########	########	Tajikistan	1449446
########	########	Tajikistan	1469704
########	########	Turkmenist	993734
########	########	Turkmenist	1022497
########	########	Timor-Leste	213341
########	########	Timor-Leste	211814
########	########	Tonga	18491
########	########	Tonga	19495
########	########	Trinidad an	404536
########	########	Trinidad an	402515
########	########	Tunisia	2930076
########	########	Tunisia	3037245
########	########	Turkey	21403049
########	########	Turkey	21447918
########	########	Tuvalu	2314
########	########	Tuvalu	2350
########	########	Tanzania	6728870
########	########	Tanzania	7191229
########	########	Uganda	4375324
########	########	Uganda	4969561
########	########	Ukraine	12570043
########	########	Ukraine	15683514
########	########	Uruguay	855959
########	########	Uruguay	963671
########	########	United Stat	91036793
########	########	United Stat	96023664
########	########	Uzbekistan	5949212
########	########	Uzbekistan	6391737
########	########	Saint Vince	27756
########	########	Saint Vince	26235
########	########	Venezuela	6702906
########	########	Venezuela	7065348
########	########	Viet Nam	22772979
########	########	Viet Nam	24364169
########	########	Vanuatu	48410
########	########	Vanuatu	46661
########	########	Samoa	38016
########	########	Samoa	35854
########	########	Yemen	4018019
########	########	Yemen	4011220
########	########	South Afric	12154965
########	########	South Afric	13353985
########	########	Zambia	2241923
########	########	Zambia	2224894
########	########	Zimbabwe	2125081
########	########	Zimbabwe	2504330

For peer review only

STROBE Statement—checklist of items that should be included in reports of observational studies

	Item No.	Recommendation	Page No.	Relevant text from manuscript
**Title and abstract**	1	(*a*) Indicate the study's design with a commonly used term in the title or the abstract	1	
		(*b*) Provide in the abstract an informative and balanced summary of what was done and what was found	2	
		**Introduction**		
Background/rationale	2	Explain the scientific background and rationale for the investigation being reported	4	
Objectives	3	State specific objectives, including any prespecified hypotheses	4	…we quantified the gap…
		**Methods**		
Study design	4	Present key elements of study design early in the paper	4	This is an ecological descriptive…
Setting	5	Describe the setting, locations, and relevant dates, including periods of recruitment, exposure, follow-up, and data collection	4-6	
Participants	6	(*a*) *Cohort study*—Give the eligibility criteria, and the sources and methods of selection of participants. Describe methods of follow-up<br>*Case-control study*—Give the eligibility criteria, and the sources and methods of case ascertainment and control selection. Give the rationale for the choice of cases and controls<br>*Cross-sectional study*—Give the eligibility criteria, and the sources and methods of selection of participants	5	Data sources
		(*b*) *Cohort study*—For matched studies, give matching criteria and number of exposed and unexposed<br>*Case-control study*—For matched studies, give matching criteria and the number of controls per case		
Variables	7	Clearly define all outcomes, exposures, predictors, potential confounders, and effect modifiers. Give diagnostic criteria, if applicable	5	Variables
Data sources/ measurement	8*	For each variable of interest, give sources of data and details of methods of assessment (measurement). Describe comparability of assessment methods if there is more than one group	5-6	Data sources and variables
Bias	9	Describe any efforts to address potential sources of bias	NA	

For peer review only

Study size	10	Explain how the study size was arrived at	NA	
Quantitative variables	11	Explain how quantitative variables were handled in the analyses. If applicable, describe which groupings were chosen and why	5	Variables
Statistical methods	12	(*a*) Describe all statistical methods, including those used to control for confounding	6	Statistical analysis
		(*b*) Describe any methods used to examine subgroups and interactions	NA	
		(*c*) Explain how missing data were addressed	NA	
		(*d*) *Cohort study*—If applicable, explain how loss to follow-up was addressed<br>*Case-control study*—If applicable, explain how matching of cases and controls was addressed<br>*Cross-sectional study*—If applicable, describe analytical methods taking account of sampling strategy	NA	
		(*e*) Describe any sensitivity analyses	NA	
**Results**				
Participants	13*	(a) Report numbers of individuals at each stage of study—eg numbers potentially eligible, examined for eligibility, confirmed eligible, included in the study, completing follow-up, and analysed	7	Data characteristics
		(b) Give reasons for non-participation at each stage	NA	
		(c) Consider use of a flow diagram	NA	
Descriptive data	14*	(a) Give characteristics of study participants (eg demographic, clinical, social) and information on exposures and potential confounders	7	Data characteristics
		(b) Indicate number of participants with missing data for each variable of interest	NA	
		(c) *Cohort study*—Summarise follow-up time (eg, average and total amount)	NA	
Outcome data	15*	*Cohort study*—Report numbers of outcome events or summary measures over time	NA	
		Case-control study—Report numbers in each exposure category, or summary measures of exposure	NA	
		Cross-sectional study—Report numbers of outcome events or summary measures	7	Underlying variables
Main results	16	(*a*) Give unadjusted estimates and, if applicable, confounder-adjusted estimates and their precision (eg, 95% confidence interval). Make clear which confounders were adjusted for and why they were included	7-9	
		(*b*) Report category boundaries when continuous variables were categorized	NA	
		(*c*) If relevant, consider translating estimates of relative risk into absolute risk for a	NA	

For peer review only

		meaningful time period		
Other analyses	17	Report other analyses done—eg analyses of subgroups and interactions, and sensitivity analyses	NA	
**Discussion**				
Key results	18	Summarise key results with reference to study objectives	9	Leveraging on high-quality data…
Limitations	19	Discuss limitations of the study, taking into account sources of potential bias or imprecision. Discuss both direction and magnitude of any potential bias	10	…there are limitations we need…
Interpretation	20	Give a cautious overall interpretation of results considering objectives, limitations, multiplicity of analyses, results from similar studies, and other relevant evidence	10-12	
Generalisability	21	Discuss the generalisability (external validity) of the study results	NA	
**Other information**				
Funding	22	Give the source of funding and the role of the funders for the present study and, if applicable, for the original study on which the present article is based	13	Funding

*Give information separately for cases and controls in case-control studies and, if applicable, for exposed and unexposed groups in cohort and cross-sectional studies.

Note: An Explanation and Elaboration article discusses each checklist item and gives methodological background and published examples of transparent reporting. The STROBE checklist is best used in conjunction with this article (freely available on the Web sites of PLoS Medicine at http://www.plosmedicine.org/, Annals of Internal Medicine at http://www.annals.org/, and Epidemiology at http://www.epidem.com/). Information on the STROBE Initiative is available at www.strobe-statement.org.

For peer review only

For peer review only - http://bmjopen.bmj.com/site/about/guidelines.xhtml
